# Anatomy and evolution of telomeric and subtelomeric regions in the human protozoan parasite *Trypanosoma cruzi*

**DOI:** 10.1186/1471-2164-13-229

**Published:** 2012-06-08

**Authors:** Roberto R Moraes Barros, Marjorie M Marini, Cristiane Regina Antônio, Danielle R Cortez, Andrea M Miyake, Fábio M Lima, Jeronimo C Ruiz, Daniella C Bartholomeu, Miguel A Chiurillo, José Luis Ramirez, José Franco da Silveira

**Affiliations:** 1 Departamento de Microbiologia, Imunologia e Parasitologia Escola Paulista de Medicina, UNIFESP, São Paulo, SP, Brazil; 2 Centro de Pesquisas René Rachou, FIOCRUZ-MG, Belo Horizonte, MG, Brazil; 3 Departamento de Parasitologia, ICB, UFMG, Belo Horizonte, MG, Brazil; 4 Decanato de Ciencias de la Salud, Universidad Centroccidental Lisandro Alvarado (UCLA), Barquisimeto, Venezuela; 5 Fundación Instituto de Estudios Avanzados – IDEA, Caracas, Venezuela

## Abstract

**Background:**

The subtelomeres of many protozoa are highly enriched in genes with roles in niche adaptation. *T*. *cruzi* trypomastigotes express surface proteins from Trans-Sialidase (TS) and Dispersed Gene Family-1 (DGF-1) superfamilies which are implicated in host cell invasion. Single populations of *T*. *cruzi* may express different antigenic forms of TSs. Analysis of TS genes located at the telomeres suggests that chromosome ends could have been the sites where new TS variants were generated. The aim of this study is to characterize telomeric and subtelomeric regions of *T*. *cruzi* available in TriTrypDB and connect the sequences of telomeres to *T*. *cruzi* working draft sequence.

**Results:**

We first identified contigs carrying the telomeric repeat (TTAGGG). Of 49 contigs identified, 45 have telomeric repeats at one end, whereas in four contigs the repeats are located internally. All contigs display a conserved telomeric junction sequence adjacent to the hexamer repeats which represents a signature of *T*. *cruzi* chromosome ends. We found that 40 telomeric contigs are located on *T*. *cruzi* chromosome-sized scaffolds. In addition, we were able to map several telomeric ends to the chromosomal bands separated by pulsed-field gel electrophoresis.

The subtelomeric sequence structure varies widely, mainly as a result of large differences in the relative abundance and organization of genes encoding surface proteins (TS and DGF-1), retrotransposon hot spot genes (RHS), retrotransposon elements, RNA-helicase and N-acetyltransferase genes. While the subtelomeric regions are enriched in pseudogenes, they also contain complete gene sequences matching both known and unknown expressed genes, indicating that these regions do not consist of nonfunctional DNA but are instead functional parts of the expressed genome. The size of the subtelomeric regions varies from 5 to 182 kb; the smaller of these regions could have been generated by a recent chromosome breakage and telomere healing event.

**Conclusions:**

The lack of synteny in the subtelomeric regions suggests that genes located in these regions are subject to recombination, which increases their variability, even among homologous chromosomes. The presence of typical subtelomeric genes can increase the chance of homologous recombination mechanisms or microhomology**-**mediated end joining, which may use these regions for the pairing and recombination of free ends.

## Background

*Trypanosoma cruzi,* the etiologic agent of Chagas disease, is a protozoan parasite that affects approximately 10 million people in Latin America*.* Trypomastigotes, the infective form of *T. cruzi,* express many surface proteins related to cell invasion and evasion of host immune response. Despite their genetic variability, surface antigens can be grouped into large gene families, such as the Trans-Sialidase (TS) superfamily and Dispersed Gene Family-1 (DGF-1) [1,2].

Although the nuclear genome of *T. cruzi* (clone CL Brener) has been sequenced [3], the large number of repetitive elements and members of multigenic families hinders the correct assembly of the parasite chromosomes. Another complicating factor in the sequence assembling process is the hybrid nature of clone CL Brener. The reference strain used in the *T. cruzi* genome sequence project clone CL Brener has a hybrid origin [4,5] and the two divergent haplotypes were named Esmeraldo-like and non-Esmeraldo-like based on a low-coverage sequence produced from the Esmeraldo strain [3]. The strain is a hybrid composed of two haplotypes, one derived from group II (Esmeraldo-like) and another from group III (non-Esmeraldo-like). Analysis of the clone CL Brener annotated dataset revealed that about 50% of sequences were found at least twice in the assembly, suggesting that they likely represent the two different haplotypes in the *T. cruzi* CL Brener genome [3]. Comparison of contigs with reads from the Esmeraldo genome, which is a member of one of the progenitor subgroups (II), allowed the two haplotypes to be identified. There is a high level of gene synteny between the two haplotypes, and the average sequence divergence between the two haplotypes is 5.4% [3].

Recently, Weatherly and coworkers [6] organized contigs and scaffolds of clone CL Brener into pairs of homologous chromosomes using *Leishmania major* and *Trypanosoma brucei* syntenic maps and BAC end sequences from *T. cruzi* genomic libraries. This effort resulted in the assembly of 41 *in silico* chromosome pairs that vary in size from 78 kb to 2.3 Mb, but their sizes can be underestimated due to the high allelic variation and the presence of repetitive sequences in the *T. cruzi* genome ([6] and http://tritrypdb.org). Therefore, some of these chromosomes may actually be part of a single chromosome [7]. Furthermore, the karyotype of clone CL Brener is composed of 20 chromosomal bands with sizes ranging from 3.27 to 0.51 Mb [7,8], which indicates that in most cases the lengths of the *in silico* chromosomes do not reflect the actual chromosomal lengths.

Eukaryotic chromosomes are characterized by the presence of free ends called telomeres. These are specialized DNA-protein complexes whose function is to stabilize chromosome ends, protecting them from nucleases and the cellular recombination machinery. *T. cruzi* telomeres are composed of a single-strand region ending in 5'-GGGTTAGGG-3' followed by tandemly arranged 9–50 double-stranded hexameric repeats (5'-TTAGGG-3') [9,10]. After the telomeric repeats, toward the centromere, there is a species-specific 189 bp sequence known as the telomeric junction [9,11]. The subtelomeric region expands between this junction and the first internal (interstitial) chromosome-specific gene. Subtelomeric regions appear to be more prone to DNA lesions and, consequently, to DNA repair and recombination. In some protozoan parasites (*T. brucei, Plasmodium falciparum* and *Giardia lamblia*), subtelomeric regions play an important role in mechanisms of antigenic variation [11-13].

We previously reported the isolation of *T. cruzi* subtelomeric regions [14] and showed that they are enriched in (pseudo)genes from the TS, DGF-1 and retrotransposon hot spot protein (RHS) families. The abundance of surface protein genes in the subtelomeric regions suggests that these regions may have acted as a site for DNA recombination, expansion and the generation of new variants of surface proteins. Members of the TS gene family display great sequence diversity and encode many surface proteins related to cell invasion, virulence, and evasion from the host immune system [2,15-17]. It has been speculated that the preferred telomeric location of the TS genes could be connected to the generation of variants via non-homologous recombination [9,18]. Kawashita and coworkers [19] proposed that members of the DGF-1 family might be associated with the ability of *T. cruzi* to bind to extracellular matrix proteins and speculated about mechanisms that could generate localized diversity in these molecules in the absence of selection. However, it remains to be established whether the telomeric location of DGF-1 genes interferes with the generation of DGF-1 variants.

In the *T. cruzi* Genome Project and TriTryp databases, contigs containing telomeric repeats have been annotated by standard automated procedures, however to define the chromosome structure of *T. cruzi*, a more accurate and detailed analysis of subtelomeric pseudogenes is needed. Filling sequence gaps should have a high priority in the completion of the *T. cruzi* Genome Project. In this work we present a detailed and individual analysis of the 49 subtelomeric regions identified in TriTrypDB. Telomeric contigs are also compared to determine the variability between them. This analysis allowed *T. cruzi* chromosome ends to be classified by the presence of TS, DGF-1 and RHS genes/pseudogenes. Homologous subtelomeric regions were also submitted to synteny analysis, which highlighted the variability in these regions even in homologous chromosomes. Finally, these telomeric contigs were mapped for the first time by hybridization with chromosome-specific markers in *T. cruzi* chromosomal bands obtained using Pulsed-Field Gel Electrophoresis (PFGE). The data presented here contribute to a greater understanding of *T. cruzi* subtelomeric regions and provide evidence of potential recombination events between chromosome ends that can generate new variants of surface antigens.

## Results

### Terminology

*Telomeres*: the tandem repeats of the hexanucleotide TTAGGG that form the chromosome tips; *telomeric junction*: a conserved sequence located adjacent to the telomere, characteristic of *T. cruzi* chromosome ends; *subtelomere* or *subtelomeric region*: the region between the hexamer repeats and the first interstitial gene; *chromosome end*: the chromosome region that comprises the telomere and subtelomere, including the telomeric junction. *Clone CL Brener*: the reference strain used in the *T. cruzi* genome sequence project. This strain has been classified into group VI and is a hybrid of group II (haplotype Esmeraldo-like) and group III (haplotype non-Esmeraldo-like). *TcChr S*: chromosome-sized scaffolds of clone CL Brener derived from the Esmeraldo-like parental haplotype. *TcChr P*: chromosome-sized scaffolds of clone CL Brener derived from the non-Esmeraldo-like parental haplotype.

### Identification and characterization of *T. cruzi* chromosome ends

We identified 49 contig sequences harboring the chromosome ends of clone CL Brener. To facilitate the description of results, chromosome ends will henceforth be referred to as telomeric contigs and abbreviated as Tel 1 to Tel 49 (Table 1 and Additional file 1). They are of different sizes, ranging from 5 to 200 kb, with telomeres ranging from 27 to 875 bp (4.5 to 145.8 hexamer repeats). As previously described by Chiurillo and coworkers [9], all the contigs displayed the conserved telomeric junction adjacent to the hexamer repeats that shares at least 70% nucleotide sequence identity between contigs (Table 1). Recently*, T. cruzi* contigs and scaffolds were assembled in 41 platforms tentatively named as *T. cruzi* chromosomes and abbreviated as TcChr [6]. This designation seemed to us inaccurate since some of these chromosomes may indeed be part of a single chromosome. For this reason, we have chosen to refer to them as chromosome-sized scaffolds and keep the TcChr abbreviation. TcChrs were assigned to the Esmeraldo and non-Esmeraldo haplotypes and designated TcChr S and TcChr P, respectively [6]. To integrate the telomeric contigs into the TcChrs, we performed a search of the TriTryp database (http://tritrypdb.org/tritrypdb/) for telomeres using the accession number (locus_id) of the gene adjacent to the telomeric repeat. Table 1 and Additional file 1 show the relationship between the telomeric contigs and TcChrs. Forty telomeric contigs were assigned to TcChrs, whereas the remaining contigs could not be fitted into chromosomal assemblies. In general, our results confirm the assembly proposed by Weatherly and coworkers [6].

**Table 1:** Summary of telomeric and subtelomeric assemblies

**Group**	**Telomeric assemble**	**Chromosome (contig)**	**Hexameric repeat (bp)**	**Junction (bp)**	**Distance to first gene (bp)^1^**	**First gene**	**Subtelomeric region size (bp)**
I	Tel1	TcChr9-P	269	185	687	RHS protein, putative	57748
	Tel2	TcChr11-S	167	185	2687	surface protease GP63, putative	125799
	Tel3	TcChr19-S	545	185	1612	TS, putative	145064
	Tel4	TcChr22-P	503	186	656	RHS protein (pseudogene), putative	108269
	Tel5	TcChr25-P	263	189	750	RNA helicase (pseudogene), putative	68130
	Tel6	TcChr28-P	257	180	840	RHS protein, putative	182470
	Tel7	TcChr38-S	353	188	705	RHS protein (pseudogene), putative	41697
II	Tel8	TcChr31-P	263	190	677	RHS protein (pseudogene), putative	55070
	Tel9	TcChr35-S	360	190	788	RHS protein, putative	66114
III	Tel10	TcChr13-P	257	186	587	SIRE	23401
	Tel11	TcChr35-P	528	190	824	RHS protein, putative	29523
IV	Tel12	TcChr6-S	27	191	659	RHS protein (pseudogene), putative	39993
	Tel13	TcChr26-P	29	148	891	RHS protein, putative	66326
	Tel14	TcChr33-S	557	188	629	RHS protein, putative	23143
	Tel15	TcChr34-S	281	186	1733	TS, putative	53258
	Tel16	TcChr35-P	691	151	-	hypothetical protein^3^	92020
	Tel17	TcChr37-S	272	188	829	RHS protein, putative	81949
	Tel18	TcChr40-P	101	184	721	RHS protein (pseudogene), putative	71586
V	Tel19	TcChr17-S	161	187	1744	TS, putative	31196
	Tel20	TcChr23-S	143	190	1010	RHS protein (pseudogene), putative	76042
	Tel21	TcChr25-S	857	184	655	RHS protein, putative	37757
	Tel22	TcChr36-P	289	191	960	RHS protein (pseudogene), putative	19098
	Tel23	TcChr39-P	179	187	694	RHS protein (pseudogene), putative	20226
VI	Tel24	TcChr11-P	545	193	414	VIPER/SIRE	23217
	Tel25	TcChr22-S	377	190	631	RHS protein (pseudogene), putative	20668
	Tel26	TcChr27-P	365	195	407	SIRE	8962
	Tel27	TcChr36-S	431	184	1555	TS, putative	28019
VII	Tel28	TcChr15-P	183	183	865	RHS protein, putative	8937
VIII	Tel29	TcChr13-S	350	190	707	RHS protein, putative	11765
	Tel30	TcChr39-S	407	189	913	RHS protein, putative	20968
IX	Tel31	Tcruzi_7430	809	145	751	RHS protein (pseudogene), putative	4543
X	Tel32	TcChr20-S	533	186	1658	TS, putative	5507
XI	Tel33^4^	Tcruzi_8424	330	193	665	RHS protein (pseudogene), putative	46301
	Tel34	TcChr25-S	420	182	672	RHS protein (pseudogene), putative	38166
	Tel35	TcChr35-S	366	186	797	RHS protein, putative	18678
	Tel36^4^	Tcruzi_149	71	189	684	RHS protein, putative	1068
	Tel37^4^	Tcruzi_2522	65	189	640	RHS protein, putative	1233
	Tel38^4^	Tcruzi_4706	109	186	545	RHS protein (pseudogene), putative	3829
	Tel39^4^	Tcruzi_6314	875	182	634	RHS protein (pseudogene), putative	1904
	Tel40^4^	Tcruzi_6749	281	184	673	RHS protein (pseudogene), putative	4104
	Tel41^4^	Tcruzi_7734	125	179	568	RHS protein (pseudogene), putative	6479
	Tel42	TcChr21-P	210	178	1745	TS, putative	34174
	Tel43	TcChr34-P	479	182	1667	TS (pseudogene), putative	5464
	Tel44^4^	Tcruzi_6797	61	185	691	RHS protein (pseudogene), putative	28339
	Tel45	TcChr40-S	180	189	837	RHS protein, putative	14078
	Tel46	TcChr19-P	185	185	836	RHS protein (pseudogene), putative	gap region
	Tel47	TcChr21-S	186	186	1641	TS, putative	66938
	Tel48^2^	TcChr27-S	193, 191	193, 191	836; 711	RHS protein, putative; RHS protein (pseudogene), putative	22552
	Tel49	TcChr31-P	187	187	642	RHS protein, putative	44331

^1^ Distance between the telomeric junction and the first gene in base pairs

^2^ This chromosomal end has two telomeric repeats followed by the telomeric junction

^3^ The first gene after the telomeric repeats is partially inserted inside the telomeric junction

^4^ Contigs harboring telomeric repeats not assembled in chromosome sized scaffolds. The subtelomeric region size indicated in the table corresponds to the whole unassigned contig sequence

**Figure 1:**
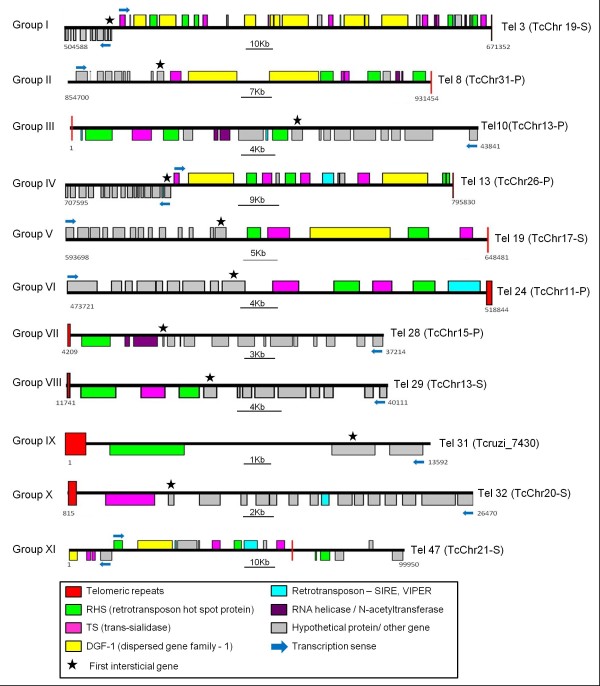
**Organization and gene content of Trypanosoma cruzi chromosome ends.** Schematic representation of *T*. *cruzi* chromosome ends (Tels) showing their distribution in eleven distinct groups according to the subtelomeric gene content. One representative member (Tel) of each group has been shown. Each color box indicates a subtelomeric gene (TriTrypDB - http://tritrypdb.org/tritrypdb/), and the red boxes denote the telomeric repeats (TTAGGG)n. The gray boxes represent interstitial genes, including genes encoding hypothetical proteins; a complete list of these genes can be found in Additional file 1. The maps are to scale and the genomic coordinates are indicated at the beginning and end of each map. Blue arrows indicate the transcription sense. Each chromosome end assembly is oriented 5’ to 3’ according to the TriTrypDB annotation.

Figure 1 and Additional file 2 summarize the present status of sequence completion for each chromosome end. Telomeric contigs were connected to chromosome-sized scaffolds. Each chromosome end assembly was oriented 5’ to 3’ according to the TriTrypDB. For this reason, in several chromosome ends the telomere is at the beginning (nucleotide position 1) whereas in others it is at the end (the last nucleotide). Details of the sequence assemblies for each chromosome end are provided in Table 1 and Additional files 1 and 2.

To define the subtelomeric regions, we scanned a 250 kb region starting from the telomeric repeats and observed all gene annotations (Table 1 and Additional file 1). The size of these regions varied widely from 5 to 182 kb among individual chromosome ends. The first annotated gene we identified was located immediately after the telomeric repeats (Table 1). In 34 of the 49 telomeric contigs, the first gene was RHS, in 8 it was TS, in 3 it was a retrotransposon and in 3 contigs the first gene was RNA helicase, gp63 or a hypothetical protein. The first gene of Tel 48 could not be determined because it contains an additional block of telomeric repeats located internally, both blocks followed by the telomeric junction. The average distance between the telomeric junction and the first telomeric gene was 918 bp and varied significantly depending on the first gene (Table 1). The sequences located closer to or farther from the telomeric junction were retrotransposons SIRE and VIPER (~400 bp) and surface protein gp63 (2,687 bp), respectively. RHS and TS were located approximately 700 and 1,600 bp, respectively, from the telomeric junction. Despite its abundance in the subtelomeric region, DGF-1 did not appear as the first gene after the junction in any of the telomeric contigs (Table 1).

**Figure 2:**
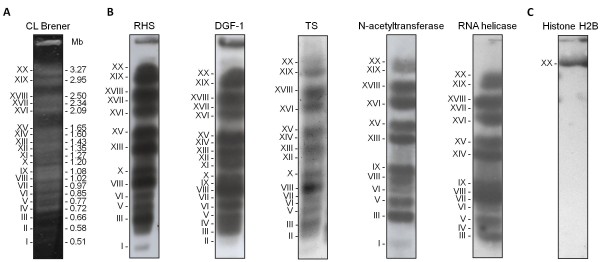
**Hybridization of subtelomeric genes with*T. cruzi*chromosomal bands. A.** CL Brener molecular karyotype obtained after separation of chromosomal bands by PFGE (1.1% agarose gel stained with ethidium bromide). The size of each chromosomal band (Mb) is indicated on the right. In accordance with the nomenclature proposed by Cano and coworkers [8], the chromosomal bands are designated by Roman numerals, starting with the smallest band. **B.** Hybridization of the chromosomal bands with subtelomeric genes: TS, trans-sialidase; RHS, retrotransposon hot spot; DGF-1, dispersed gene family-1; **C.** Hybridization of the chromosomal bands with a single copy gene control (Histone H2B).

RHS, TS, DGF-1, retrotransposons, ATP-dependent DEAD/H RNA helicase and N-acetyltransferase are the most abundant sequences in the subtelomeric region. Approximately 34% and 19% of all RNA helicase and RHS sequences in the genome, respectively, were found in the subtelomeric regions (Table 2), and the other families were also well represented. Interestingly, less than 1% of mucin and mucin-associated proteins (MASPs) were found in these regions (Table 2 and Additional file 1). Southern blot hybridization of chromosomal bands separated by PFGE with probes derived from RHS, TS, DGF-1, RNA helicase and N-acetyltransferase confirmed the presence of these sequences in most of the chromosomal bands (Figure 2).

**Table 2:** **Gene frequency in chromosome ends of*T. cruzi*genome**

	**Genomic copy number (pseudogenes)**	**Subtelomeric copy number (pseudogenes)**	**Percentage of subtelomeric copies**
Retrotransposon Hot Spot (RHS)^1^	752 (557)	141 (103)	19%
Trans-sialidases^1^	1430 (693)	127 (96)	9%
Dispersed gene family-1 (DGF-1)^1^	565 (136)	70 (33)	12%
ATP-dependent DEAD/H RNA helicase^2^	151 (141)	19 (17)	12,5%
N-acetyltransferase complex ARD1 subunit^2^	41 (38)	14 (13)	34%
MASP^1^	1377 (433)	7 (6)	0,50%
Mucins^1^	863 (201)	3 (2)	0,35%

^1^*Trypanosoma cruzi* genome project data [3]

^2^TriTrypDB data (http://tritrypdb.org/tritrypdb/)

A search for orthologs of typical subtelomeric genes in other trypanosomatids (*T. brucei* and *L. major*) revealed that these genes are specific to *T. cruzi* (Additional file 1). These data reinforce the hypothesis that chromosome ends may have been hot-spots for DNA recombination that contributed to the divergence between these protozoan parasites.

### Classification of *T. cruzi* chromosome ends according to the gene content of the subtelomeric regions

Comparison of all *T. cruzi* chromosome ends indicated that they can be classified into 11 groups (I to XI) according to the gene content of the subtelomeric region (Table 1, and Additional files 1 and 2). Figure 1 show the complex patchwork of sequence blocks shared by *T. cruzi* chromosome ends. The overall size, sequence content and organization of subtelomeres relative to the terminal hexameric repeat tracts and the subtelomeric single-copy DNA are different for each subtelomere. All the subtelomeric gene families are present in group I whereas only one subtelomeric gene, TS or RHS, is present in groups IX and X. The most representative groups in the databases are I and IV with seven contigs each. The telomeric contigs assigned to groups II and III contain members of five subtelomeric families with the difference that DGF-1 and retrotransposons are lacking in groups II and III, respectively (Figure 1). Groups IV, V-VII and VIII contain two to four members of the subtelomeric families. Finally, group XI comprises four chromosome ends (Tels 46 to 49) in which the telomeric repeats are located internally in the contig (Figure 1). Two hypotheses could explain the presence of telomeric repeats within the contigs: 1) an error occurred during the *in silico* sequence alignment, resulting in the integration of two different chromosome ends in the same contig or 2) a merge of different telomeres occurred in the parasite genome, forming chromosomes with internal telomeric sequences. In some contigs the size of the subtelomeric region has not yet been determined (Tel 33 to Tel 45). Tentatively, these were placed in group XI.

Analysis of subtelomeric region maps shows that RHS, DGF-1 and TS are often duplicated. The most common organization is the presence of one or more TS genes flanked by RHS genes. Most ATP-dependent DEAD/H RNA helicase and N-acetyltransferase genes are found together within the subtelomeres, with the N-acetyltransferase gene located close to telomere.

### Synteny analysis between homologous chromosome ends of *T. cruzi*

**Figure 3:**
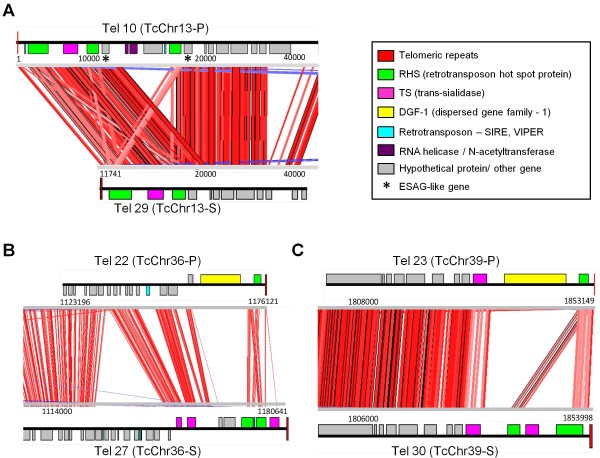
**Synteny analysis between homologous chromosome ends of T. cruzi.** Comparison of the ends of homologous chromosomes TcChr13-P and S **(panel A)**; TcChr36-P and S **(panel B)** and TcChr39-P and S **(panel C)**. Chromosome ends Tel10 and Tel 29 are located in the homologous chromosomes TcChr13-P and TcChr13-S; Tel 22 and Tel 27 in the homologous TcChr36-P and TcChr36-S and Tel 23 and Tel 30 in the homologous TcChr39-P and TcChr39-S. The red lines represent regions of homology between the contigs. The annotated genes are indicated by colored boxes. The ESAG-like genes are indicated by the black star.

Out of 49 chromosome ends examined, 27 were assigned to 12 homologous chromosome-sized scaffolds (TcChr) available in the TriTryp database (Table 3). Using the ACT program (Artemis Comparison Tool), available at the Sanger Institute (http://www.sanger.ac.uk/resources/soft ware/act/), we were able to compare the degree of synteny between six homologous pairs located at the same chromosomal extremity (Tables 3 and Additional file 3). This analysis disclosed synteny breaks at the subtelomere. There is a high degree of synteny in the interstitial regions of homologous chromosomes, which is broken in the subtelomeres. Chromosome ends Tel 10 and Tel 29 share a syntenic block located in the subtelomere immediately after the hexamer repeats and were assigned to the homologous pair TcChr13-P and TcChr13-S, respectively. The synteny is disrupted after the second RHS gene by the insertion of a block containing RNA helicase, N-acetyltransferase and hypothetical protein genes (Figure 3A). The inserted block is followed by RHS and ESAG (Expression Site Associated Genes)-like sequences. These sequences were first described in *T. brucei* and are located next to the telomeres, close to the Variant Surface Glycoprotein (VSG) sites. They are related to recombination and expression of surface protein genes in this parasite [13]. The finding of ESAG-like sequences next to *T. cruzi* telomeres reinforces the hypothesis of the occurrence of recombination events in the subtelomeric regions in this parasite. The presence of the RHS gene adjacent to ESAG-like sequences suggests that the former could have been the target for a recombination event that broke the synteny.

**Table 3:** Homologous chromosomes with telomeric repeat

**Chromosome/ contig (TriTryp)**	**Chromosomal end**	**Telomeric repeat location in chromosome-size contig**
TcChr11-P	Tel 24	3' end
TcChr11-S	Tel 2	5' end
TcChr13-P	Tel 10	5' end
TcChr13-S	Tel 29	5' end
TcChr19-P	Tel 46	internal
TcChr19-S	Tel 3	3' end
TcChr21-P	Tel 42	5' end
TcChr21-S	Tel 47	internal
TcChr22-P	Tel 4	5' end
TcChr22-S	Tel 25	3' end
TcChr25-P	Tel 5	5' end
TcChr25-S	Tel 34	5' end
TcChr25-S	Tel 21	3' end
TcChr27-P	Tel 22	3' end
TcChr27-S	Tel 48	internal
TcChr34-P	Tel 43	5' end
TcChr34-S	Tel 15	5' end
TcChr35-P	Tel 16	5' end
TcChr35-S	Tel 35	5' end
TcChr35-P	Tel 11	3' end
TcChr35-S	Tel 9	3' end
TcChr36-P	Tel 22	3' end
TcChr36-S	Tel 27	3' end
TcChr39-P	Tel 23	3' end
TcChr39-S	Tel 30	5' end
TcChr40-P	Tel 18	5' end
TcChr40-S	Tel 45	3' end

Chromosome ends Tel 22-Tel 27 and Tel 23-Tel 30 were assigned to the homologous chromosome pairs TcChr36-S and 36-P and TcChr39-S and 39-P, respectively. Subtelomeres from homologous chromatids (for instance, TcChr 36-S and 36-P) can differ largely in size and gene content (Figure 3B and C). Since clone CL Brener is a hybrid which has two haplotypes, this difference could be explained by the fact that the homologous chromosomes are derived from different haplotypes. Therefore, the chromosome-sized scaffolds assigned to the Esmeraldo and non-Esmeraldo haplotypes were designated S and P, respectively. Syntenic analysis showed that there was a high degree of synteny conservation between the interstitial regions and that this synteny is broken in the subtelomeres (Figure 3B and C). The pattern of homology between interstitial regions with a synteny break in the chromosome ends was observed in all homologous chromosome ends analyzed (Additional file 3).

We are aware that high coverage is necessary to confirm a syntenic break within homologous chromosomes and to produce a comparable contig assembly. In this work we analyzed the degree of synteny between six homologous chromosome pairs located at the same chromosomal extremity (Figure 3 and Supplementary Figure 3). In two of them (TcChr 36 – Tels 22 and 27 and TcChr 39 – Tels 23 and 30), which are shown in Figure 3, the assembly was confirmed by sequencing at high coverage, reinforcing the hypothesis of the occurrence of a syntenic break at the telomeric end. The chromosome ends of the pair TcChr 13-S and 13-P (Tels 10 and 29) were sequenced to high coverage except for the breakpoint in the homologue TcChr 13-P, which was sequenced to low coverage. With respect to other chromosome ends, there was low-coverage sequence data (Tels 5, 11, 15, 19, 22 and 27) or one of the chromosome ends was interrupted before the interstitial region - for example, in TcChr 25-S (Tel 34), TcChr 34-P (Tel 43) and TcChr 35-S (Tel 35) (Supplementary Figure 3). Although such regions cannot be unambiguously resolved by the available data, they could be associated with the breaks in synteny. This finding confirms the problems involved in the assembly of the *T. cruzi* sequence due to the high allelic variation and the presence of repetitive sequences in the genome [3].

### Mapping of chromosome ends to chromosomal bands separated by PFGE

**Figure 4:**
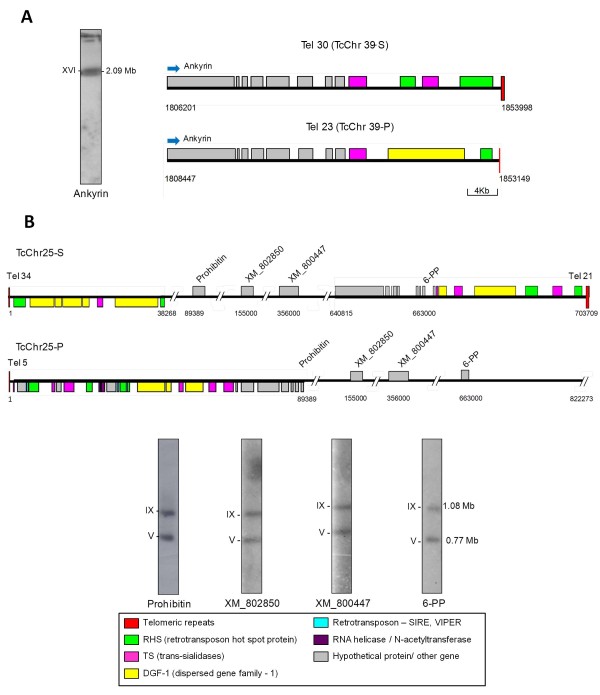
**Integration of*in silico*data with the*T. cruzi*molecular karyotype: mapping of chromosome ends to the chromosomal bands separated by PFGE. A.** Schematic representation showing the location of chromosome ends Tel 30 and Tel 23 in the homologous chromosomes TcChr39-P and TcChr39-S, respectively. The left panel shows the hybridization of marker ankyrin with the chromosomal bands of clone CL Brener separated by PFGE. The probe hybridized only to chromosomal band XVI, indicating that both homologous chromosomes TcChr39-P and TcChr39-P are located in the same chromosomal band. **B.** Chromosome ends Tel 34 and Tel 21 are located in chromosome TcChr25-S and Tel 5 in chromosome TcChr25-P. The hybridization of subtelomeric marker glucanolactonase-6PP, found in Tel 21, the prohibitin found in Tel 5 and interstitial markers XM_802850 and XM_800447 with the chromosomal bands separated by PFGE is shown at the bottom. The markers hybridized with chromosomal bands V and IX, indicating that the homologs of chromosome TcCh25 are of different sizes. The maps are to scale and the genomic coordinates are indicated at the beginning and end of each map. Each chromosome end assembly is oriented 5’ to 3’ according to the TriTrypDB annotation. Blue arrows indicate the transcription sense.

Using chromosome-specific markers, the chromosome ends were mapped into chromosomal bands of clone CL Brener separated by PFGE (Figure 4). In this context the term chromosomal bands refers to bands separated by PFGE, which are visualized after staining with ethidium bromide. A chromosomal band can contain comigrating non-homologous chromosomes; and homologous chromosomes can migrate separately. As previously shown, the telomeric contigs Tel 30 and 23 are at the 3’ ends of the homologous pair TcChr39-S and TcChr39-P, respectively (see Figure 4A). The ankyrin gene, located at the interstitial region flanking chromosome ends Tel 30 and Tel 23, hybridized with chromosomal band XVI (2.09 Mb), indicating that these two homologous chromosomes are located in this band and are the same size (Figure 4A). Tel 34 and 21 are at the extreme opposite ends of chromosome TcChr25-S, and Tel 5 are the 5’ extremity of TcChr25-P (Figure 4B). Markers located in the interstitial regions flanking the chromosome ends –prohibitin gene located at the 5’ end, XM_802850 and XM_800447 at the middle and 6-phosphogluconolactonase (6-pp) gene at the 3’ end– hybridized with chromosomal bands V (0.77 Mb) and IX (1.08 Mb), suggesting that TcChr25-S and TcChr25-P constitute a heteromorphic pair of homologous chromosomes. As mentioned above clone CL Brener is a hybrid that contains two haplotypes (S and P). It was not possible to define whether TcChr25-S and TcChr25-P are located on bands V and IX because markers prohibitin, XM_802850, XM_800447 and 6-pp are present in both haplotypes.

### Structural and functional analysis of subtelomeric gene families

We studied the structure and function of subtelomeric gene families in clone CL Brener. Approximately 9% of TS, 12% of DGF-1 and 19% of RHS genes annotated in the genome were located at chromosome ends (Table 2 and Additional file 1). Out of 565 copies of DGF-1 in the *T. cruzi* genome, 70 copies were found at the chromosome ends. Among the subtelomeric copies of DGF-1, 37 were intact and 33 truncated (Table 2 and Additional file 4). The complete copies showed transmembrane and signal peptide domains, suggesting a structural role for this protein. DGF-1 subtelomeric copies were always accompanied by RHS genes. There are 141 subtelomeric RHS sequences, most of which are pseudogenes (n=103). The complete RHS copies (n=38) have characteristic features of the family, such as the ATP/GTP binding motif and an insertion site for retrotransposons (Additional file 5). Although the abundance of RHS and DGF families on the *T. cruzi* genome, especially next to the telomeres, proteins codified by these genes still not have a clear biological function [20,21].

Fifty-three copies of retrotransposons, most of them VIPER/SIRE elements, were identified at the subtelomeres (Additional file 6). VIPER is an LTR-like retroelement associated with SIRE, a short interspersed repetitive element specific of *T. cruzi*[22]. We also found 3 non-LTR elements (1 NARTc and 2 L1Tc). All retrotransposons found at chromosome ends appeared to be non-functional copies.

TS genes represent the largest *T. cruzi* gene family, with 1,430 genes distributed throughout the genome that can be grouped into four groups (TS I to IV) with different characteristics [1,2]. Of the 127 TS subtelomeric sequences here analyzed, 31 are complete genes, while 96 copies of sequences are incomplete genes. All the groups of the TS superfamily are represented in the subtelomeric regions; most of the sequences (n=83) are members of group II (GP82, GP85, TC85), which includes 22 complete genes (Table 4). Group I of the TS family is represented only by three genes, all truncated; group III is represented by 31 genes, 7 of which are complete; and group IV by 10 genes, 2 of which are complete (Table 4 and Additional file 7). Recently, Freitas and coworkers [23] performed a sequence cluster analysis of all complete trans-sialidase genes and identified four additional groups. We also classified the complete subtelomeric TS genes according this new clustering (see Additional file 7). There is a good agreement (79%) between our classification and that proposed by these authors. There was only one exception to this: 7 TS subtelomeric sequences in group III were distributed into groups VII (1 TESA gene) and VIII (6 complement regulatory proteins genes).

**Table 4:** Summary of telomeric trans-sialidases

**Gene**	**Complete**	**Incomplete**	**Total**
	**(signal peptide)**		
**TS - group I**			
TS-1	0 (0)	3	3
group total	0 (0)	3	3
**TS - group II**			
Gp82	7 (6)	2	9
Gp90	3 (3)	2	5
Gp85	2 (2)	4	6
Tc85/SA85	7 (7)	12	19
ASP-1	0 (0)	3	3
ASP-2	3 (3)	36	39
ASP-3	0 (0)	1	1
ASP-4	0 (0)	1	1
group total	22 (21)	61	83
**TS - group III**			
CRP	6 (4)	17	23
FL160	0 (0)	4	4
TESA	1 (1)	3	4
group total	7 (5)	24	31
**TS - group IV**			
Tc13	1 (1)	2	3
C71	0	4	4
Tcc1J12	1 (1)	2	3
group total	2 (2)	8	10
**Total**	**31 (28)**	**96**	**127**

Previous studies have reported the presence of TS genes in the chromosome ends of *T. cruzi* but failed to establish whether there are expressed genes [14]. Our results confirm the abundance of (pseudo) genes of the GP85 subfamily as well as genes and (pseudo) genes of other TSs, such as GP82 (9 genes) and Complement Regulatory Protein (23 genes). It is important to note that 31 complete TS genes could be expressed by the parasite, most of them bear a signal peptide (28 genes) and the acceptor site for the GPI anchor (28 genes). These proteins are found on the surface of the parasite and play a role in cell invasion and/or mammalian host immune evasion.

Transcripts of subtelomeric genes can be found in *T. cruzi* databases. To confirm whether subtelomeric genes (trans-sialidases, RHS, DGF-1, RNA helicases and N-acetyltransferase) were translated, we searched for peptides in the expressed protein database of *T. cruzi* (TriTrypDB), which contains peptides identified by mass spectrometry. In this search we did not include sequences from truncated genes or pseudogenes. Of the 31complete TS genes, 17 (54.8%) showed mass spectrometry-based evidence of gene expression; all belonged to TS group II (Additional file 8). Of the 37 complete DGF-1 genes, 11 (~30%) were found in protein databases, and 32 (84.2%) of the 38 RHS complete genes were translated (Additional file 8). We did not identify any peptides for subtelomeric RNA helicases or N-acetyltransferase in these databases. Taken together, these results suggest that *T. cruzi* subtelomeric regions could contain expression sites, especially for RHS, DGF-1 and TS from group II.

## Discussion

In higher eukaryotes the telomeric repeat array extends several kilobases from the chromosome ends [24,25], whereas in protozoans and fungi it is much shorter, averaging 130–350 bp. We estimated the average length of the *T. cruzi* telomere repeats to be ~ 320 bp (53.3 repeats) (Table 1). However, their lengths varied widely among telomeric contigs (6 to 142 repeats). In a previous work, Freitas-Junior and coworkers [10] experimentally observed a great variability in the length of telomeric repeats in the CL Brener clone, ranging from 1 to 10 Kb. The smaller size of the sequences identified *in silico* may have been caused by slippage artifacts during BAC replication in *Escherichia coli* cells or by the difficulty assembling small repeat sequences obtained by whole genome shotgun sequencing*,* both methods used in the *T. cruzi* genome project [3].

Despite the sequence variations, similar telomeric structures have been detected in almost all *T. cruzi* chromosomes studied to date. The telomeric junction, a signature for *T. cruzi* telomeres, was present in all chromosome ends, confirming this sequence as a signature sequence of *T. cruzi* chromosomes [9]. In a previous work we suggested that the events that generated the common *T. cruzi* telomeric block could be reconstructed from events that occurred at a tandem array of gp85 genes [14] as follows: first, a deletion brought together a fragment containing the spacer between two gp85 genes and part of a gp85 5’-UTR, with the 3’- UTR of the same gene; subsequently, a break took place in the 3’ UTR generating an end that was healed by telomerase or an alternative telomere repair mechanism; eventually these two structures were fixed as the *T. cruzi* telomere. In the present study, the size of the subtelomere varied widely from 5 kb to 182 kb among individual *T. cruzi* chromosome ends, and the organization of several subtelomeres, for instance, Tel 31 and Tel 32 (Figure 1), suggests that they have undergone truncation and that this could be a general phenomenon in *T. cruzi*.

We were able to identify 49 chromosome ends harboring the telomeric repeats in clone CL Brener, 40 located in chromosome-sized scaffolds and 9 in unassigned contigs. The number of chromosome ends found is smaller than we had expected; however, it is worth mentioning that about 50% of the *T. cruzi* genome is composed of multigenic families and repetitive sequences [3] and as the chromosome ends are enriched with these sequences they are very difficult to assemble. For this reason there are still a number of small unassigned contigs harboring typical subtelomeric genes or hexamer repeats that were not analyzed in this work. It should also be highlighted that the chromosome-sized scaffolds of *T. cruzi* are useful for sequence analysis and constitute an important tool for defining the linear gene sequence of the parasite. However, in most cases they do not reflect the actual chromosomal lengths and are in fact part of a single chromosome [7]. Our in-depth analysis of telomeric and subtelomeric regions showed that the *T. cruzi* chromosome end structure varies widely as a result of differences in the abundance and organization of surface protein coding genes (TS and DGF-1) and RHS, retrotransposon, RNA-helicase and N-acetyltransferase genes. All the 425 complete genes within the subtelomeric region were present at more than one chromosome end. For example, RHS sequences were distributed in 47 subtelomeres, TS in 39, retrotransposons and DGF-1 in 29, RNA helicase in 16 and N-acetyltransferase in 11 chromosome ends. Therefore, it seems that switching mechanisms operated in *T. cruzi* to generate new variants of these gene families.

Comparison of *T. cruzi* homologous chromosomes showed that synteny breaks down around the subtelomeric region, reinforcing the hypothesis that frequent recombination events occurred between subtelomeric regions of this parasite. Adjacent to the telomeric repeats is a mosaic of surface protein coding sequences and RHS, retrotransposon, RNA-helicase and N-acetyltransferase genes that exhibit a great deal of polymorphism both between termini of an individual chromosome or between different chromosome ends (see Figure 1). In *T. brucei*, chromosomal rearrangements have been associated with the presence of RHS genes and retrotransposons [26]. *T. cruzi* chromosome-sized scaffolds TcChr13-P and TcChr13-S are syntenic up to the beginning of the subtelomeric region, where the synteny is broken by the insertion of a 7 Kb region flanked by RHS genes. Apparently, the RHS sequences were duplicated during the insertion, suggesting that homologous recombination had occurred. The mosaicism in subtelomeric regions in *T. cruzi* chromosomes could be due to some common underlying mechanism. It is reasonable to suggest that there may be a selective advantage to maintaining the chromosome end polymorphism or a common active mechanism that leads to the accumulation and maintenance of mosaicism. Recently Souza and coworkers [7] reported extensive variation in genome size and karyotype polymorphism among *T. cruzi* lineages. They observe that *T. cruzi* lineages exhibit conservation of chromosome structure and synteny indicating that the variability found in the subtelomeric regions are typical of these chromosomal regions.

Confirming the findings of previous studies, RHS sequences were found flanking DGF-1 and TS genes. All subtelomeric copies of DGF-1 were flanked by RHS or TS sequences. DGF-1 genes were organized in tandem, with multiple copies flanked by RHS and/or TS sequences. The organization of RHS genes flanking surface protein genes (TS and/or DGF-1) may suggest that these sites have been involved in the generation of new surface protein variants of the parasite. The repetitive sequences present in the RHS genes and pseudogenes might be a target for homologous recombination or microhomology-mediated end joining, allowing the generation of variants by recombination of different chromosome ends.

In addition, we confirmed that RHS, DGF-1, TS, DEAD/H-RNA helicase and N-acetyltransferase sequences are abundant in subtelomeric regions of *T. cruzi*[9,14]. For instance, 19%, 12% and 9%, respectively, of RHS, DGF-1 and TS sequences of the whole genome were found in the subtelomeric regions. Thirty-four and 12%, respectively, of N-acetyltransferase and DEAD/H-RNA helicase sequences were also located in these regions, indicating that they too could be considered characteristic markers for the subtelomeric regions (Table 2). Despite great abundance in *T. cruzi* genome mucins and MASP are poorly found in the subtelomeric regions. Helicases are essential molecular motor enzymes involved in processes requiring the separation of nucleic acid strands. They are classified into six different superfamilies according to the presence of conserved motifs. Both RNA-helicase and RecQ helicase belong to superfamily 2, the largest family, which is implicated in diverse cellular processes, including telomere maintenance [27]. In yeast ATP-dependent DEAD/H RNA helicases are part of complexes involved in mRNA decapping and deadenylation [28].

Recently in *T. cruzi,* ATP-dependent DEAD/H RNA helicases have been found in RNA in stress granules that may be involved in RNA metabolism and whose cell distribution seemed to be developmentally regulated [29]. Considering the polycistronic nature of Kinetoplastida transcription, a fine tuning of gene expression during cell cycle has to be exerted post-transcriptionally. Therefore, mRNA processing is a critical step in the parasite’s survival, and the machinery involved in this process can be considered an essential mechanism of regulation.

In protozoan parasites, especially *T. brucei* and *P. falciparum*, the role of subtelomeric regions in the generation of new variants of surface antigen genes and the control of expression of these genes has been widely demonstrated [11-13]. In *P. falciparum,* telomeres are followed by a non-coding region called TAS (telomere associated sequence) that consists of six blocks of repetitive sequences – TAREs (telomere associated repetitive elements). Upstream TASs are members of multigene families that encode virulence factors, like the var gene family. Each cell has up to 70 different var genes, and differential expression of these allows the escape of the parasite from the immune system by a mechanism known as antigenic variation [30-32]. In *T. brucei*, surface glycoprotein genes – VSG (variant surface glycoprotein) - were identified near telomeric repeats, and each trypanosome encodes up to a thousand different VSGs [13,20]. Parasite survival in mammalian hosts results from a sophisticated strategy of antigenic variation that involves switching the glycoprotein coat [33]. It was not possible to identify similar organizational patterns in *T. cruzi* chromosome ends, and no active transcriptional promoters have been identified to date in this parasite. However, as observed in *T. brucei*, retrotransposons and RHS genes are commonly located next to subtelomeric surface antigen genes and could have acted as a recombination site.

In the chromosome ends of *T. cruzi* there are a large number of genes and pseudogenes annotated as trans-sialidases (TS) with no further specifications. The TS superfamily is divided into four groups with different biological functions [1,2,34]. In the present study, all the members of these four groups were identified in the chromosome ends, genes from group II being the most abundant. This group comprises proteins that function as surface-located adhesins involved in host cell invasion [1,2,35]. Freitas and coworkers [23] also described the presence of gp85, gp82, gp90 and ASP-2 genes in the subtelomeric regions of *T. cruzi.* These genes could be a target for recombination, generating genetic variability and reinforcing the hypothesis of the participation of subtelomeric regions in the generation of new variants of surface antigens. Here, TS genes and pseudogenes flanked on both sides by RHS genes were observed in several chromosome ends. This organization is suggestive of the repetitive regions adjacent to VSG genes in *T. brucei* telomeres [36], where the repetitive sequences are involved in recombination mechanisms responsible for antigenic variation [37-39]. Perhaps a similar mechanism for generating gene diversity existed in *T. cruzi* that produced the surface antigens variability that we currently observe.

Complete copies of TS (31) and DGF-1 (37) genes, some of them larger than 10 kb, were identified in the subtelomeric regions, indicating that these regions are sites for generation and storage of variant surface antigens and that they can also act as active transcription sites for these genes. Subtelomeric genes are transcribed towards the telomeric repeats in all the chromosome ends analyzed (Additional file 2). In some chromosome ends analyzed the inversion of transcription sense was observed at the beginning of the interstitial region. In this work we have described a detailed analysis of the structure and organization of chromosome ends in *T. cruzi* and have confirmed the abundance of surface protein genes flanked by repetitive sequences at the subtelomeric regions. It is tempting to suggest that these regions acted as a gene reservoir and recombination site responsible for the large number of surface gene variants in *T. cruzi* and play an important role in the parasite adaptation and evasion of the host immune system.

Finally, we would like to make some considerations regarding the state of the assembly of the *T. cruzi* genome. The results presented in this work highlight the complexity of the *T. cruzi* genome and the difficulties involved in carrying out a more in-depth analysis of the chromosome structure of this parasite. We carried out an initial analysis of a set of subtelomeric sequence assemblies which were properly ordered and positioned in relation to the respective telomeres. This allows comparison of subtelomeric sequence organization of a few separate telomeres. Although the *in silico* chromosome assemblies were of great value for analysis, they should be improved by re-sequencing of selected regions and analysis by Comparative Genomic Hybridization (CGH) [40]. Sequencing of new strains of *T. cruzi* coupled with the CGH technique can highlight deleted and/or amplified regions along the chromosome [40]. For the subtelomeric region, and also possibly other repeated regions of the genome, this effort should be complemented by the cloning of genomic fragments in traditional vectors such as BAC, since the high-throughput DNA sequencing of the whole *T. cruzi* genome produced relatively short telomeric contigs.

## Conclusions

Our results indicate that there is extensive genetic variation between *T. cruzi* chromosome ends. This includes the size of subtelomeric regions and relative abundance and organization of genes encoding surface proteins, retrotransposon hot spot genes, retrotransposon elements, RNA-helicase, and N-acetyltransferase genes.

Comparison of homologous chromosomes showed that synteny breaks down around the subtelomeric region, reinforcing the hypothesis that frequent recombination events occurred between subtelomeric regions of this parasite, and suggesting a new functional definition of subtelomeric regions as those terminal places where chromosomal synteny is lost.

## Methods

### Parasite

*T. cruzi* CL Brener clone was used throughout this study [41]. Parasites were maintained by cyclic passage in mice and axenic cultures at 28 °C in liver-infusion tryptose medium (LIT) containing 10% fetal calf serum.

### Separation of *T. cruzi* chromosomal DNA by PFGE

Separation of *T. cruzi* chromosomal DNA by PFGE was performed as previously described [8]. Briefly, 1x10^7^ epimastigote cells from *T. cruzi* were immobilized in 1% low-melting point agarose and incubated with a solution containing 0.5 M EDTA (pH 8.0), 1% sodium lauryl sarcosinate (Sarkosyl)and 1 mg/mL proteinase K at 50 °C for 48 h. PFGE was carried out on 1.1% agarose gel in 0.5X TBE (45 mM Tris; 45 mM boric acid; 1 mM EDTA, pH 8.3) at 13 °C for 132 h using the Gene Navigator System (Amersham Pharmacia Biotech, NJ, USA) and a hexagonal electrode array.

Gels were stained with ethidium bromide (0.5 μg/mL) and photographed. DNA samples were incubated with 0.25 M HCl for 45 min, denatured with 0.5 M NaOH/1 M NaCl for 20 min, neutralized with 1 M Tris-base/0.5 M NaCl for 20 min and transferred to nylon membranes in 20X SSC (1X SSC = 0.15 M NaCl and 0.015 M sodium citrate). The membranes were hybridized as described below.

### Hybridization

Membranes were pre-hybridized in a solution containing 50% formamide/5X SSC/5X Denhardt’s solution (Invitrogen)/0.1 mg/mL salmon sperm DNA/0.1 mg/mL tRNA at 42 °C for 1 h and hybridized overnight at 42 °C with ^32^P-labeled probes. Following hybridization, membranes were subjected to two washes (30 min each at 42 °C) in 2X SSC containing 0.1% SDS and 0.1% sodium pyrophosphate and two additional washes at 56 °C in 0.1X SSC containing 0.1% SDS and 0.1% sodium pyrophosphate. They were then exposed to X-ray film. The following sequences were used as probes: DGF-1 (Tc00.1047053508283.69), Histone H2B (Tc00.1047053511635.20), N-acetyltransferase (Tc00.1047053504149.210), RHS (Tc00.1047053506129.80) and RNA helicase (Tc00.1047053511473.6) TS (Tc00.1047053510543.200).

### *In silico* analysis: identification of the telomeric contigs in *T. cruzi* chromosome-sized scaffolds and synteny analysis

All sequences used throughout this work are from clone CL Brener, which is available in TriTrypDB. Analysis of the 41 *in silico* chromosome pairs (TcChr 1 to 41) and also the unassigned contigs deposited in TriTrypDB resulted in the identification of 49 telomeric contigs. As TriTrypDB data were used in the analysis, the nomenclature for *in silico* chromosomes (TcChr 1 to 41) proposed by Weatherly and coworkers [6] was maintained throughout the manuscript. The chromosome-sized contigs containing the telomeric repeats were identified using the gene ID number from telomeric contigs available in the TriTrypDB website. The contigs selected by this approach were used to identify typical subtelomeric genes and define the length of the subtelomeric region. Data from each gene, including gene annotation and genomic locus, were used to construct maps of the chromosome ends with the DNAMAN program (http://www.lynnon.com/). The telomeric junction was identified using BLAST (bl2seq) (http://blast.ncbi.nlm.nih.gov). Synteny analyses were performed by aligning telomeric contigs using Artemis Comparison Tool (ACT) (http://www.sanger.ac.uk/resources/software/act/) [42].

Sequences from the TS, RHS and DGF gene families annotated in the chromosome ends were collected from TriTrypDB and individually analyzed to search for transcripts. Sequences containing uninterrupted ORFs (open reading frames) larger than 300 bp were considered potentially expressed and submitted to further analysis (see below). Sequences containing small or interrupted ORFs were assumed to be incomplete or pseudogenes.

Classification of subtelomeric TS sequences into the groups of the TS superfamily [2,34] was carried out with BLASTx and BLASTp using the parameters established by [36]. To identify potential sites for addition of a GPI anchor and signal peptide, sequences from the TS, DGF-1 and RHS families were analyzed with FragAnchor (http://navet.ics.hawaii.edu/ fraganchor ~/NNHMM/NHMM.html) [43,44] and Signal IP 3.0 (http://www.cbs.dtu.dk/services SignalP/) [45], respectively. DGF-1 sequences were also analyzed with TMHMM Server v. 2.0 (http://www.cbs.dtu.dk/services/TMHMM/) [46] to search for transmembrane domains. The presence of ATP/GTP binding motifs (TPGIGKS) and retrotransposon insertion sites (LLY) was investigated in RHS sequences. Retrotransposons (RT) were identified in chromosome ends using Repbase from the GIRI database website (http://www.girinst.org/censor/index.php) with the Repeat Masker algorithm [47]. RT elements were analyzed individually to search for complete active elements. *T. cruzi* proteins identified by proteomic analysis were available at TriTrypDB (http://tritrypdb.org/tritrypdb/showQuestion.do?questionFullName=GeneQuestions.GenesByMassSpec). Peptides coded by subtelomeric genes (trans-sialidases, RHS, DGF-1, RNA helicases and N-acetyltransferase) were detected in mass spectrometry databases using the gene id number. Data generated in this work will be submitted to TriTrypDB (http://tritrypdb.org/tritrypdb/).

## Competing interests

The authors declare that they have no competing interests.

## Authors’ contributions

RRMB and MMM participated in the experimental design, bioinformatics analysis and annotation, data analysis and preparation of the manuscript. CRA and AMM assisted with the analysis and classification of DNA sequences from chromosome ends. DRC assisted with physical mapping experiments. JCR performed synteny analysis. DCB provided sequence data and performed synteny analysis. MAC contributed reagents, materials and analysis tools. JLR contributed reagents, materials and analysis tools. JFS designed, coordinated and supervised the study and participated in the interpretation and discussion of results and the preparation of the manuscript. All authors read and approved the final manuscript.

## Acknowledgements

This work was supported by grants from FAPESP and CNPq (Brazil) to JFS. RRMB was awarded a doctoral fellowship by FAPESP and MMM was awarded a postdoctoral fellowship by FAPESP. CRA is an undergraduate student and was awarded an undergraduate research scholarship by FAPESP. DCM was awarded a master’s fellowship by FAPESP.

## Supplementary Material

Additional file 1 **Gene annotation of*T. cruzi*subtelomeric assemblies.** A complete list of chromosome ends, including gene annotation, locus ID, locus size, transcription sense in the chromosome-sized scaffolds (TcChr) and genomic location. Click here for file

Additional file 2 **Schematic representation of*T. cruzi*chromosome ends.** Schematic maps of *T. cruzi* chromosome ends analyzed in this study. The red boxes represent the telomeric repeats (TTAGGG). Each colored box represents a single annotated gene (TriTrypDB - http://tritrypdb.org/tritrypdb/) as indicated in the figure. The maps are to scale and the genomic coordinates are indicated. Blue arrows indicate the transcription sense. Each chromosome end is oriented 5’ to 3’ according to the TriTrypDB annotation. The chromosome ends were separated into eleven distinct groups according to the gene content of the subtelomeric region. Click here for file

Additional file 3 **Synteny analysis between homologous chromosome ends of*T. cruzi*.** Synteny analysis between the homologous chromosome ends listed in Table 3. The red lines represent regions of homology between the contigs. The annotated genes are indicated by colored boxes. Click here for file

Additional file 4 **Subtelomeric DGF-1 genes.** A list of DGF-1 genes found in the subtelomeric regions, including annotation (TriTryp DB), locus_id, locus size, chromosome-sized scaffolds (TcChr) location, genomic location and gene integrity. Click here for file

Additional file 5 **Subtelomeric RHS genes.** A complete list of RHS family genes found in the subtelomeric regions, including annotation (TriTrypDB), locus_id, locus size, chromosome-sized scaffolds (TcChr) location, genomic location and gene integrity. Click here for file

Additional file 6 **Subtelomeric retrotransposons.** A complete list of retroelements identified in the subtelomeric regions, including annotation (GIRI DB), locus size, chromosome-sized scaffolds (TcChr) location, genomic location and gene integrity. Click here for file

Additional file 7 **Subtelomeric trans-sialidases (TS).** A complete list of trans-sialidase family members identified in the subtelomeric regions, including locus_id, locus size, chromosome-sized scaffolds (TcChr) location, genomic location, transcript classification, gene integrity and classification according Freitas et al. [23]. Click here for file

Additional file 8 **Evidence for translation of subtelomeric genes.** A complete list of genes identified by mass spectrometry-based evidence of gene expression of TriTrypDB, including locus_id, locus size, chromosome-sized scaffolds (TcChr) location, genomic location, gene annotation and life cycle stage in which the peptide was isolated. Click here for file
