# Supplementary material for: Anatomy and evolution of telomeric and subtelomeric regions in the human protozoan parasite Trypanosoma cruzi
Source: BMC Genomics. 2012 Jun 8;13:229. doi: 10.1186/1471-2164-13-229 (PMC3418195; doi:10.1186/1471-2164-13-229)
Supplement: Additional file 2 — Schematic representation ofT. cruzichromosome ends. Schematic maps of T. cruzi chromosome ends analyzed in this study. The red boxes represent the telomeric repeats (TTAGGG). Each colored box represents a single annotated gene (TriTrypDB - http://tritrypdb.org/tritrypdb/) as indicated in the figure. The maps are to scale and the genomic coordinates are indicated. Blue arrows indicate the transcription sense. Each chromosome end is oriented 5’ to 3’ according to the TriTrypDB annotation. The chromosome ends were separated into eleven distinct groups according to the gene content of the subtelomeric region. [file 1471-2164-13-229-S2.pptx]

## Slide 1
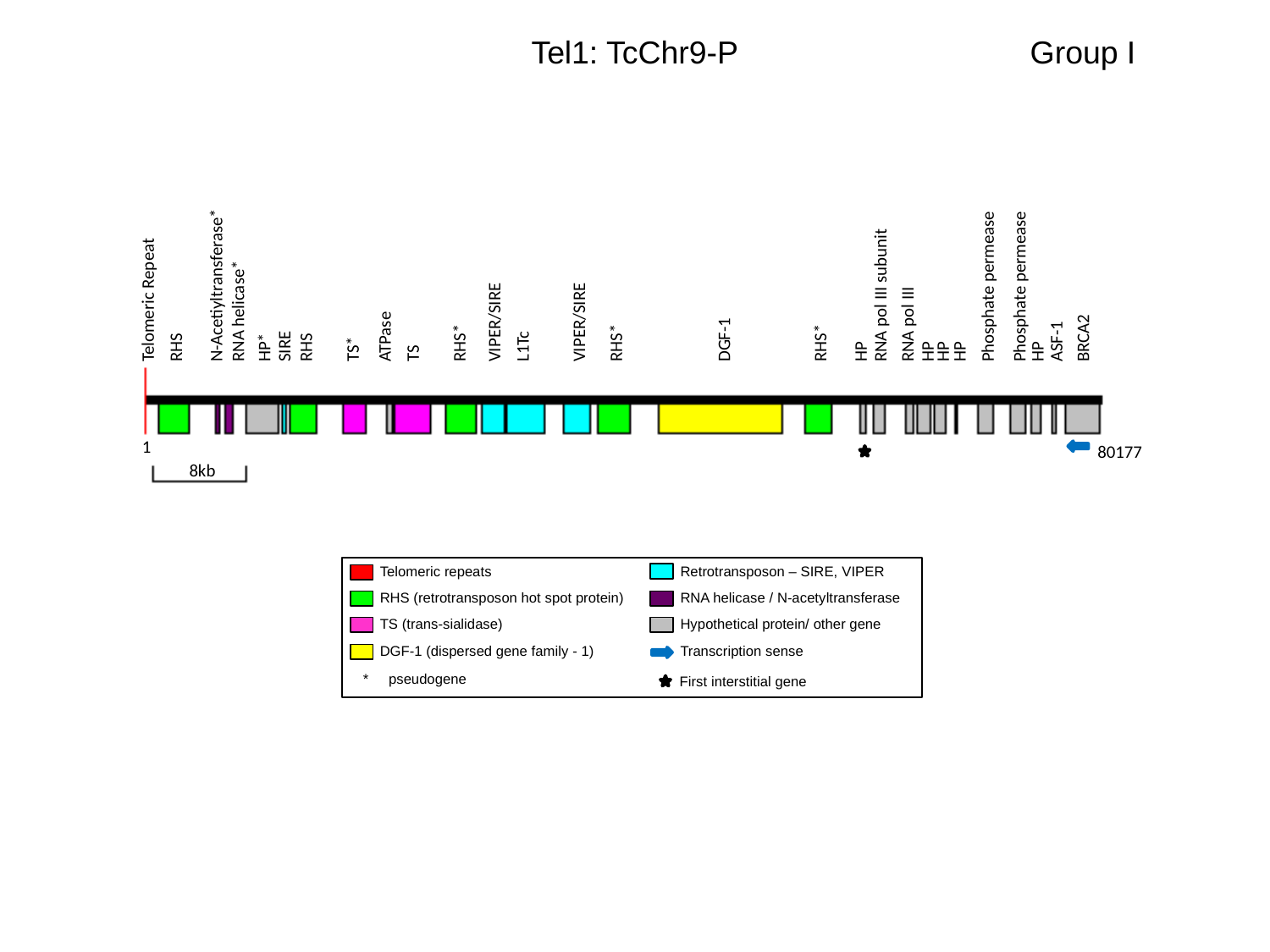

Tel1: TcChr9-P
Group I
RHS
N-Acetiyltransferase*
RNA helicase*
HP*
SIRE
RHS
TS*
ATPase
TS
RHS*
VIPER/SIRE
L1Tc
VIPER/SIRE
RHS*
RHS*
HP
RNA pol III subunit
RNA pol III
HP
HP
HP
Phosphate permease
Phosphate permease
HP
ASF-1
BRCA2
Telomeric Repeat
DGF-1
1
80177
8kb
Telomeric repeats
Retrotransposon – SIRE, VIPER
RHS (retrotransposon hot spot protein)
RNA helicase / N-acetyltransferase
TS (trans-sialidase)
Hypothetical protein/ other gene
DGF-1 (dispersed gene family - 1)
Transcription sense
* pseudogene
First interstitial gene

## Slide 2
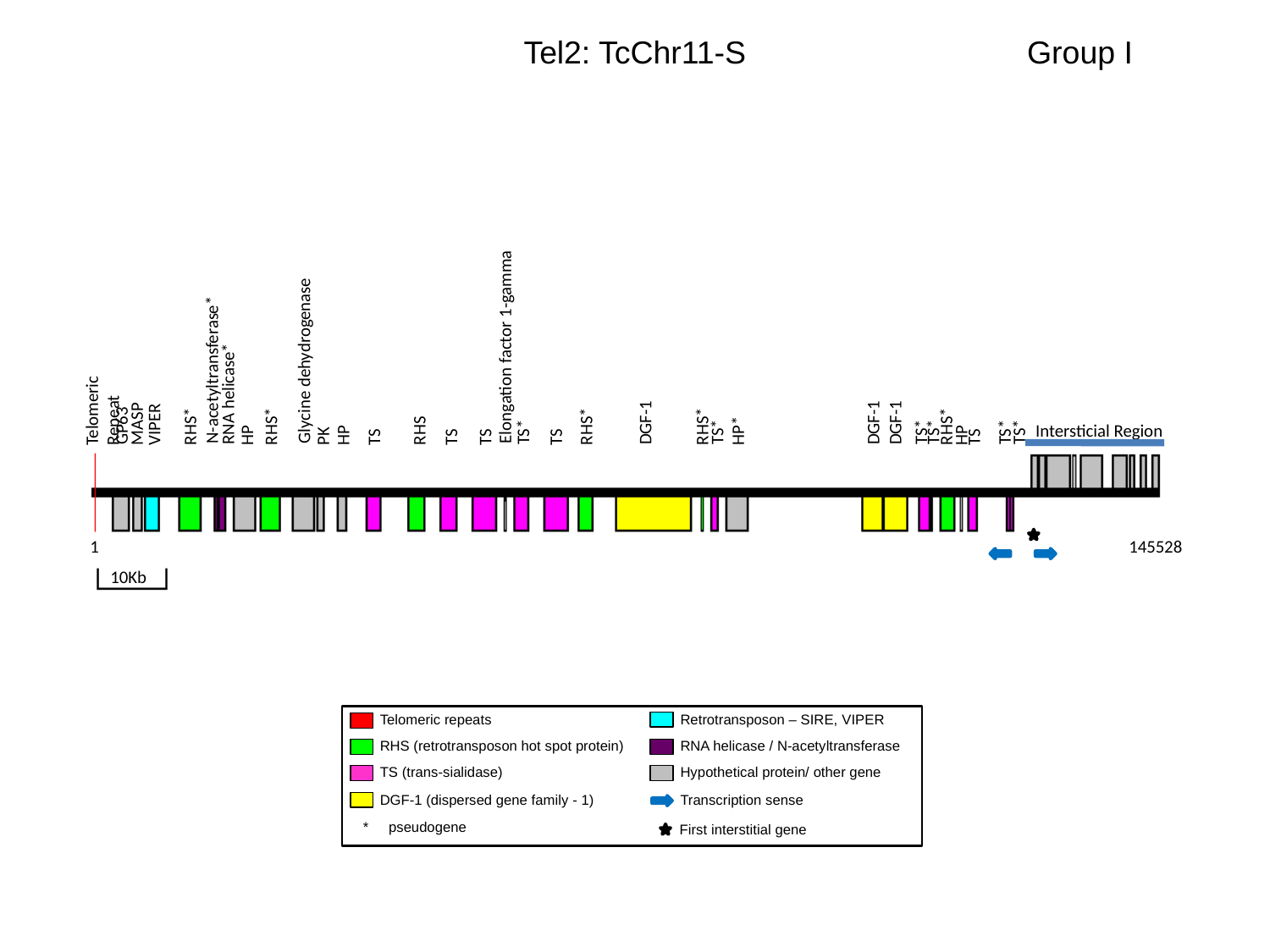

Tel2: TcChr11-S
Group I
Telomeric Repeat
Elongation factor 1-gamma
Glycine dehydrogenase
N-acetyltransferase*
RNA helicase*
DGF-1
DGF-1
DGF-1
MASP
VIPER
GP63
RHS*
RHS*
RHS*
RHS*
RHS*
Intersticial Region
HP*
RHS
TS*
TS*
TS*
TS*
TS*
TS*
HP
HP
HP
PK
TS
TS
TS
TS
TS
1
145528
10Kb
Telomeric repeats
Retrotransposon – SIRE, VIPER
RHS (retrotransposon hot spot protein)
RNA helicase / N-acetyltransferase
TS (trans-sialidase)
Hypothetical protein/ other gene
DGF-1 (dispersed gene family - 1)
Transcription sense
* pseudogene
First interstitial gene

## Slide 3
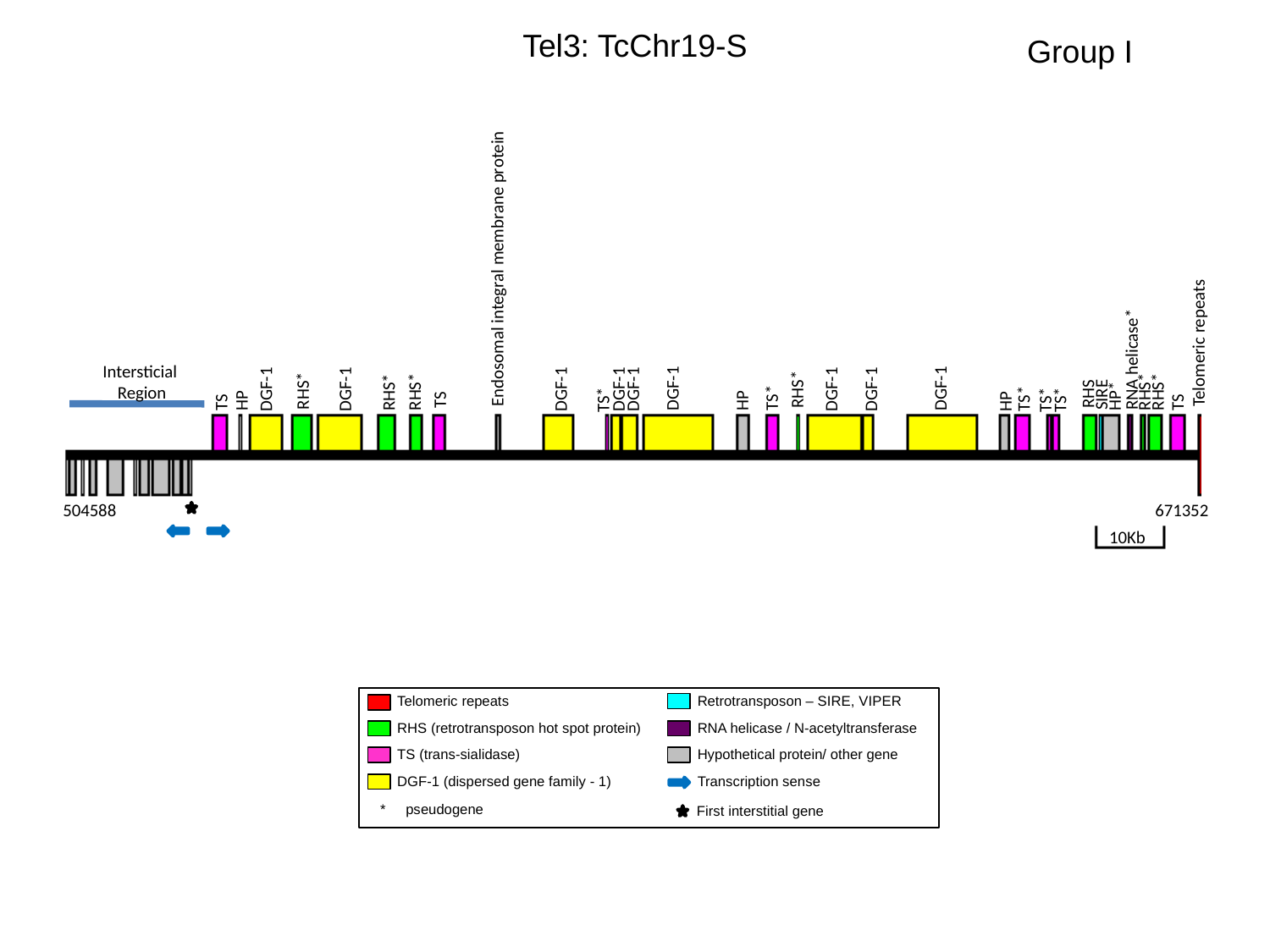

Tel3: TcChr19-S
Group I
Endosomal integral membrane protein
Telomeric repeats
RNA helicase*
Intersticial
Region
DGF-1
DGF-1
DGF-1
DGF-1
DGF-1
DGF-1
DGF-1
RHS*
DGF-1
DGF-1
RHS*
RHS*
RHS*
RHS*
RHS*
RHS
SIRE
HP*
TS*
TS*
TS
TS*
TS*
TS*
HP
HP
HP
TS
TS
671352
504588
10Kb
Telomeric repeats
Retrotransposon – SIRE, VIPER
RHS (retrotransposon hot spot protein)
RNA helicase / N-acetyltransferase
TS (trans-sialidase)
Hypothetical protein/ other gene
DGF-1 (dispersed gene family - 1)
Transcription sense
* pseudogene
First interstitial gene

## Slide 4
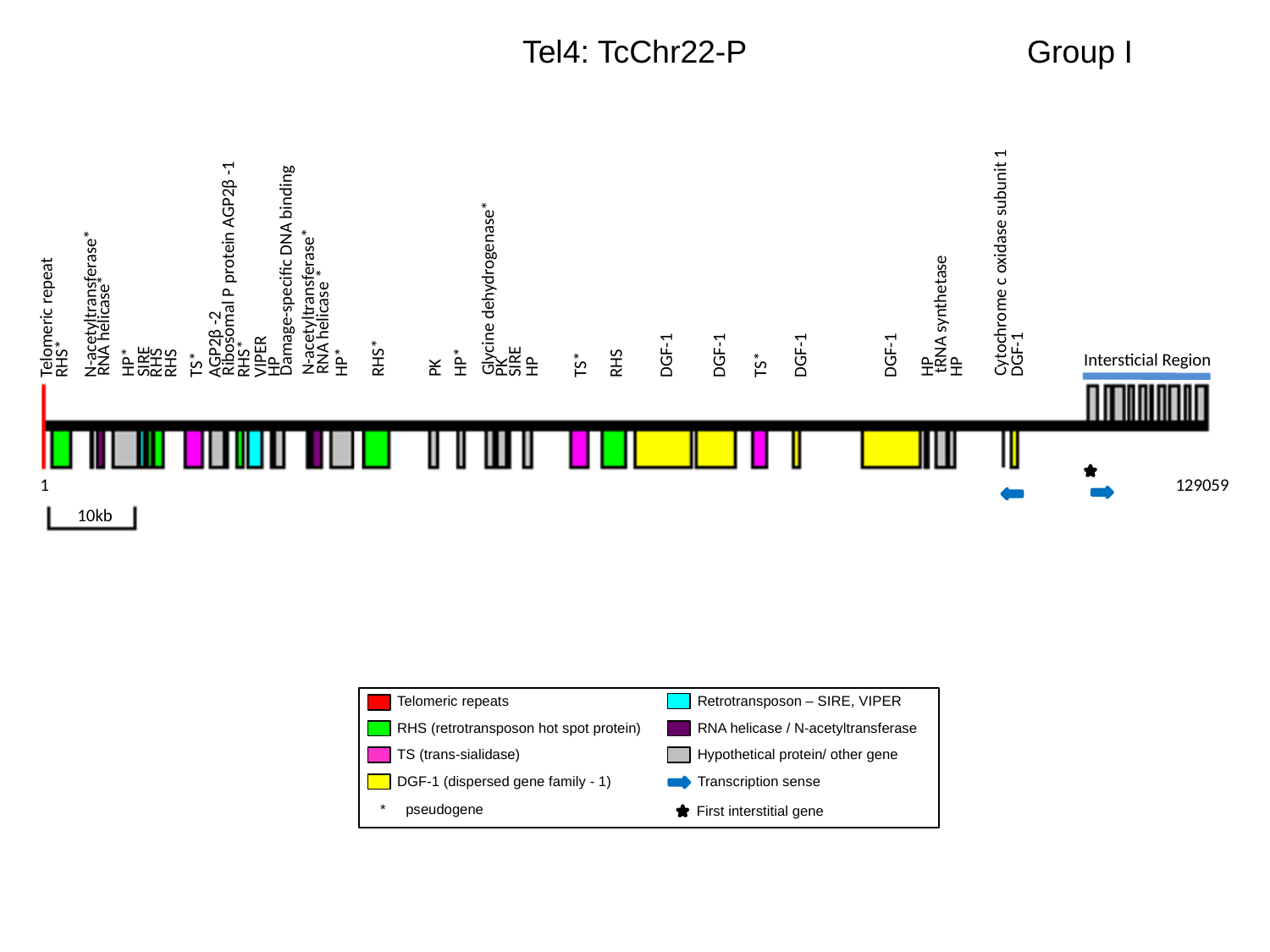

Tel4: TcChr22-P
Group I
Cytochrome c oxidase subunit 1
Ribosomal P protein AGP2β -1
Damage-specific DNA binding
Telomeric repeat
Glycine dehydrogenase*
N-acetyltransferase*
N-acetyltransferase*
tRNA synthetase
RNA helicase *
RNA helicase*
AGP2β -2
RHS*
DGF-1
DGF-1
TS*
RHS
RHS*
DGF-1
TS*
RHS
RHS
DGF-1
VIPER
DGF-1
TS*
RHS*
Intersticial Region
SIRE
SIRE
HP*
HP*
HP*
HP
HP
HP
HP
PK
PK
1
129059
10kb
Telomeric repeats
Retrotransposon – SIRE, VIPER
RHS (retrotransposon hot spot protein)
RNA helicase / N-acetyltransferase
TS (trans-sialidase)
Hypothetical protein/ other gene
DGF-1 (dispersed gene family - 1)
Transcription sense
* pseudogene
First interstitial gene

## Slide 5
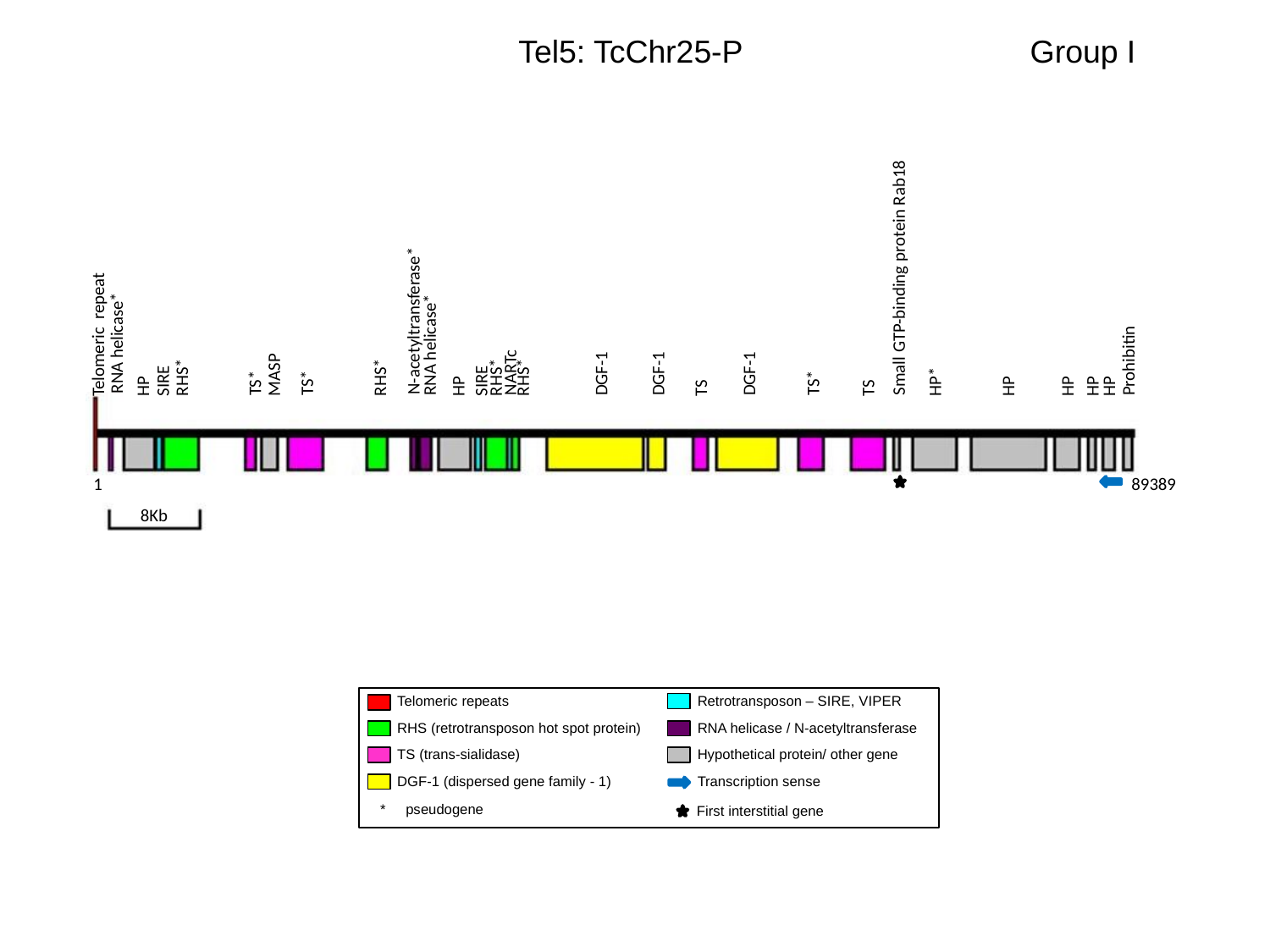

Tel5: TcChr25-P
Group I
Small GTP-binding protein Rab18
Telomeric repeat
N-acetyltransferase*
RNA helicase*
RNA helicase*
Prohibitin
NARTc
DGF-1
DGF-1
DGF-1
MASP
RHS*
RHS*
RHS*
RHS*
SIRE
SIRE
HP*
TS*
TS*
TS*
HP
HP
HP
HP
HP
HP
TS
TS
1
89389
8Kb
Telomeric repeats
Retrotransposon – SIRE, VIPER
RHS (retrotransposon hot spot protein)
RNA helicase / N-acetyltransferase
TS (trans-sialidase)
Hypothetical protein/ other gene
DGF-1 (dispersed gene family - 1)
Transcription sense
* pseudogene
First interstitial gene

## Slide 6
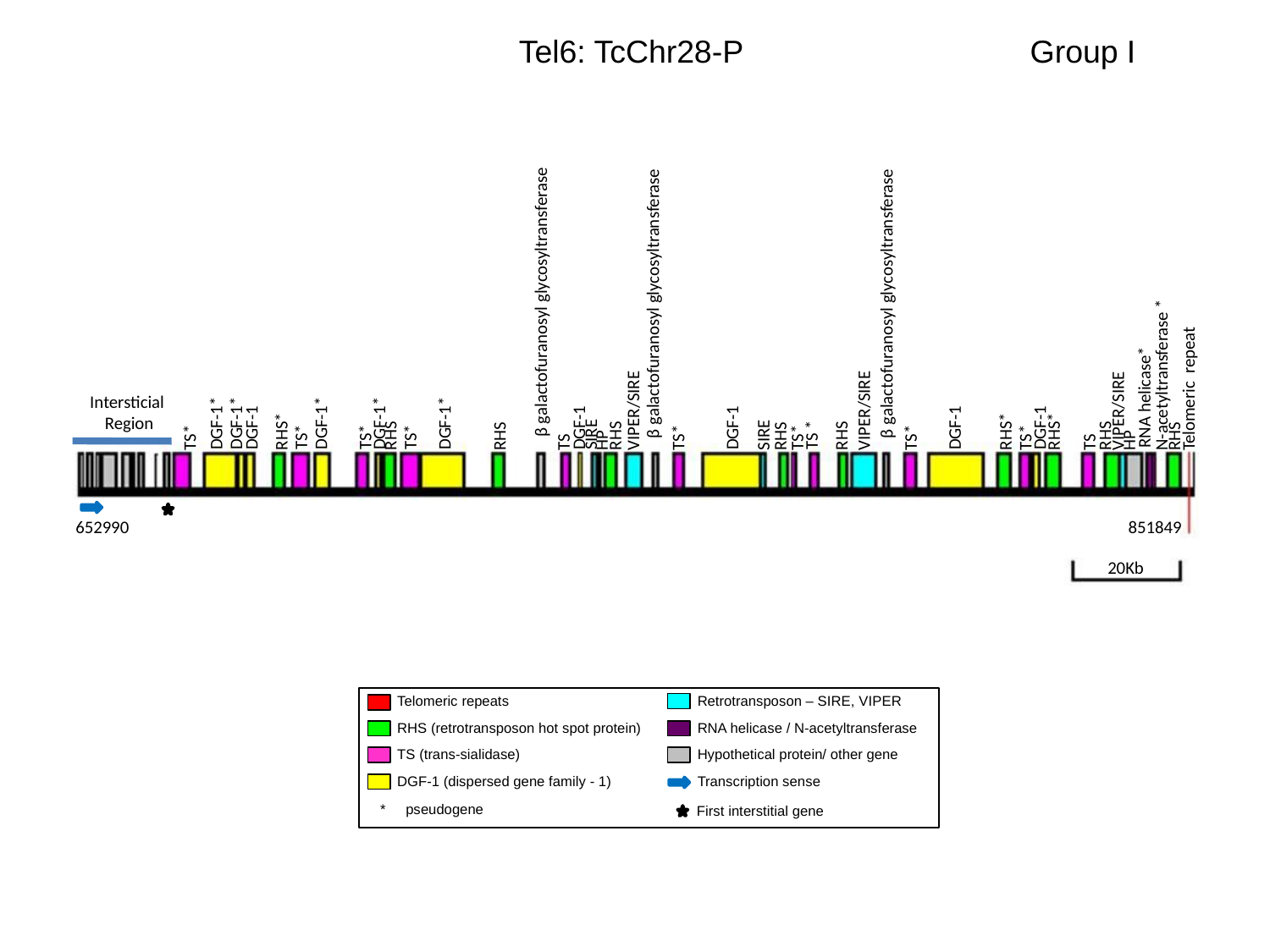

Tel6: TcChr28-P
Group I
N-acetyltransferase *
β galactofuranosyl glycosyltransferase
β galactofuranosyl glycosyltransferase
β galactofuranosyl glycosyltransferase
RHS
Telomeric repeat
VIPER/SIRE
 RNA helicase*
Intersticial
Region
VIPER/SIRE
VIPER/SIRE
DGF-1*
DGF-1*
DGF-1*
DGF-1*
DGF-1*
DGF-1
DGF-1
DGF-1
DGF-1
DGF-1
RHS*
RHS*
RHS*
SIRE
SIRE
TS *
RHS
RHS
RHS
RHS
RHS
RHS
TS*
TS*
TS*
TS*
TS*
TS*
TS*
TS*
HP
HP
TS
TS
652990
851849
20Kb
Telomeric repeats
Retrotransposon – SIRE, VIPER
RHS (retrotransposon hot spot protein)
RNA helicase / N-acetyltransferase
TS (trans-sialidase)
Hypothetical protein/ other gene
DGF-1 (dispersed gene family - 1)
Transcription sense
* pseudogene
First interstitial gene

## Slide 7
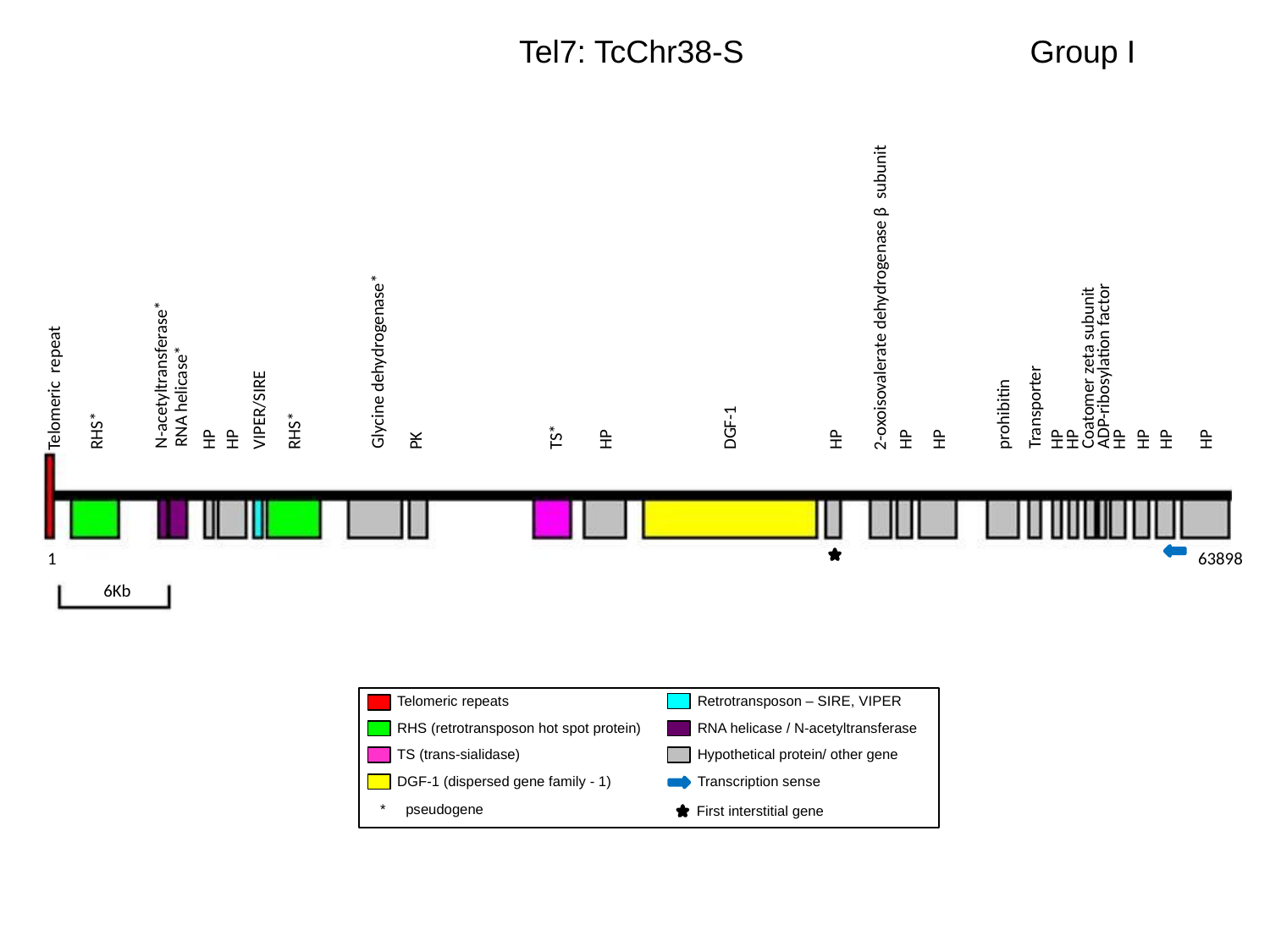

Tel7: TcChr38-S
Group I
2-oxoisovalerate dehydrogenase β subunit
Telomeric repeat
Glycine dehydrogenase*
ADP-ribosylation factor
Coatomer zeta subunit
N-acetyltransferase*
RNA helicase*
Transporter
VIPER/SIRE
prohibitin
DGF-1
RHS*
RHS*
TS*
HP
HP
HP
HP
HP
HP
HP
HP
HP
HP
HP
HP
PK
1
63898
6Kb
Telomeric repeats
Retrotransposon – SIRE, VIPER
RHS (retrotransposon hot spot protein)
RNA helicase / N-acetyltransferase
TS (trans-sialidase)
Hypothetical protein/ other gene
DGF-1 (dispersed gene family - 1)
Transcription sense
* pseudogene
First interstitial gene

## Slide 8
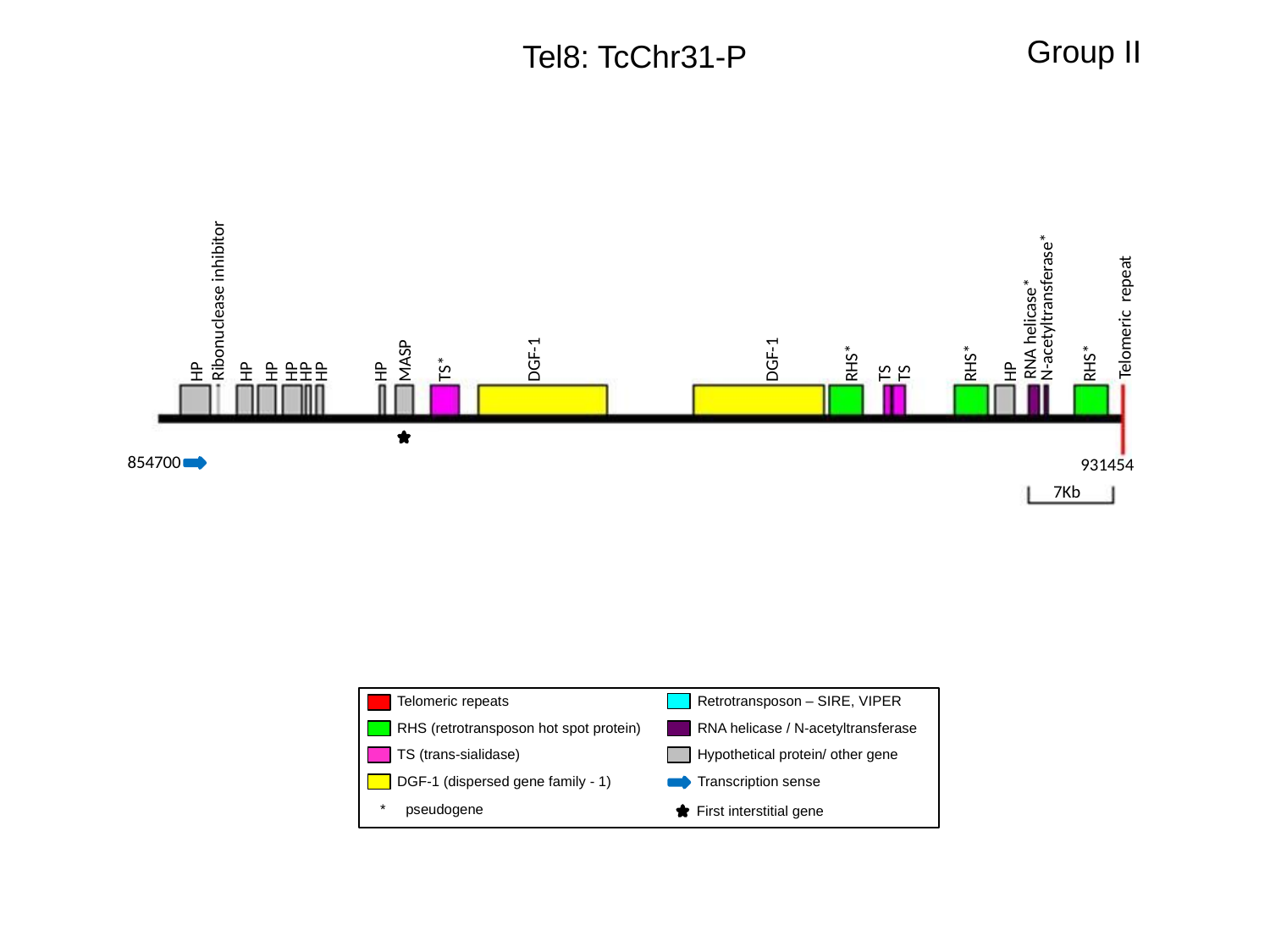

Group II
Tel8: TcChr31-P
RHS*
Ribonuclease inhibitor
N-acetyltransferase*
Telomeric repeat
RNA helicase*
HP
HP
HP
DGF-1
DGF-1
HP
HP
HP
HP
MASP
TS*
RHS*
RHS*
HP
TS
TS
854700
931454
7Kb
Telomeric repeats
Retrotransposon – SIRE, VIPER
RHS (retrotransposon hot spot protein)
RNA helicase / N-acetyltransferase
TS (trans-sialidase)
Hypothetical protein/ other gene
DGF-1 (dispersed gene family - 1)
Transcription sense
* pseudogene
First interstitial gene

## Slide 9
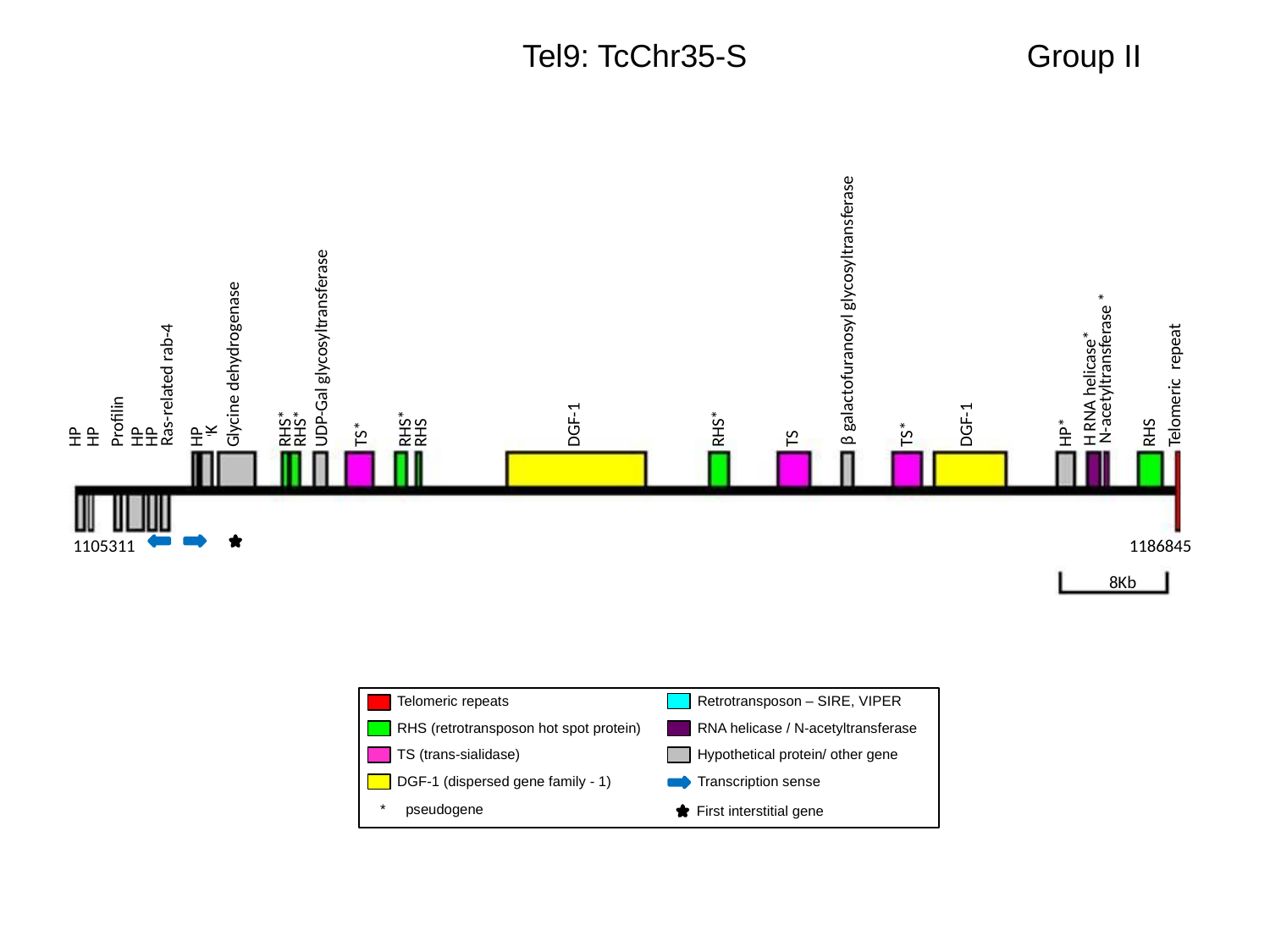

Tel9: TcChr35-S
Group II
β galactofuranosyl glycosyltransferase
UDP-Gal glycosyltransferase
Glycine dehydrogenase
RHS*
RHS*
RHS*
RHS
RHS*
RHS
Telomeric repeat
N-acetyltransferase *
Ras-related rab-4
H RNA helicase*
Profilin
DGF-1
DGF-1
HP*
TS*
TS*
HP
HP
HP
HP
HP
TS
1105311
1186845
8Kb
PK
Telomeric repeats
Retrotransposon – SIRE, VIPER
RHS (retrotransposon hot spot protein)
RNA helicase / N-acetyltransferase
TS (trans-sialidase)
Hypothetical protein/ other gene
DGF-1 (dispersed gene family - 1)
Transcription sense
* pseudogene
First interstitial gene

## Slide 10
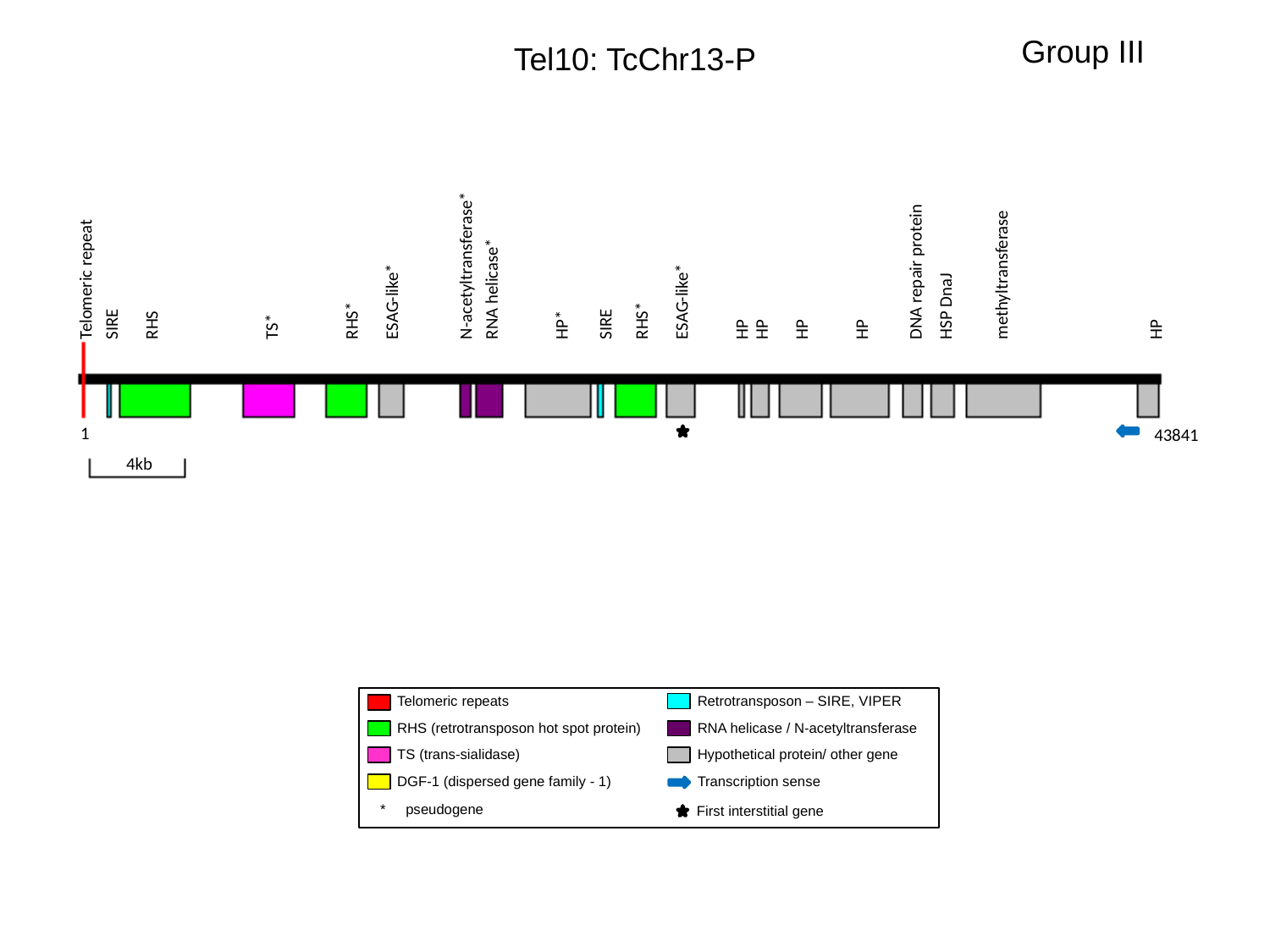

Group III
Tel10: TcChr13-P
N-acetyltransferase*
Telomeric repeat
SIRE
RHS
TS*
RHS*
ESAG-like*
SIRE
RHS*
ESAG-like*
methyltransferase
DNA repair protein
RNA helicase*
HSP DnaJ
HP*
HP
HP
HP
HP
HP
1
43841
4kb
Telomeric repeats
Retrotransposon – SIRE, VIPER
RHS (retrotransposon hot spot protein)
RNA helicase / N-acetyltransferase
TS (trans-sialidase)
Hypothetical protein/ other gene
DGF-1 (dispersed gene family - 1)
Transcription sense
* pseudogene
First interstitial gene

## Slide 11
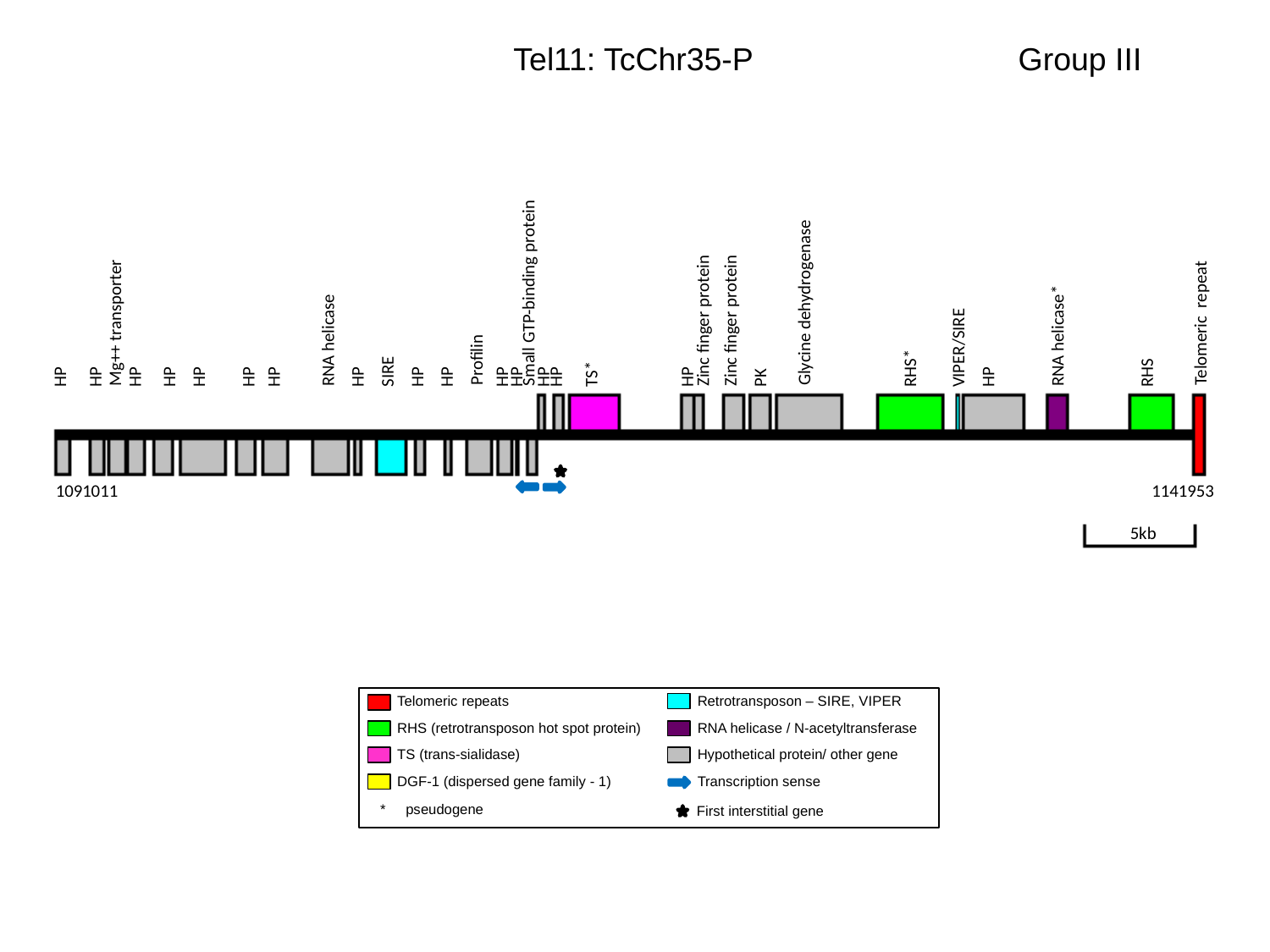

Tel11: TcChr35-P
Group III
Small GTP-binding protein
Glycine dehydrogenase
VIPER/SIRE
Zinc finger protein
Zinc finger protein
Telomeric repeat
Mg++ transporter
RNA helicase*
RNA helicase
Profilin
RHS*
SIRE
RHS
TS*
HP
HP
HP
HP
HP
HP
HP
HP
HP
HP
HP
HP
HP
HP
HP
HP
PK
1091011
1141953
5kb
Telomeric repeats
Retrotransposon – SIRE, VIPER
RHS (retrotransposon hot spot protein)
RNA helicase / N-acetyltransferase
TS (trans-sialidase)
Hypothetical protein/ other gene
DGF-1 (dispersed gene family - 1)
Transcription sense
* pseudogene
First interstitial gene

## Slide 12
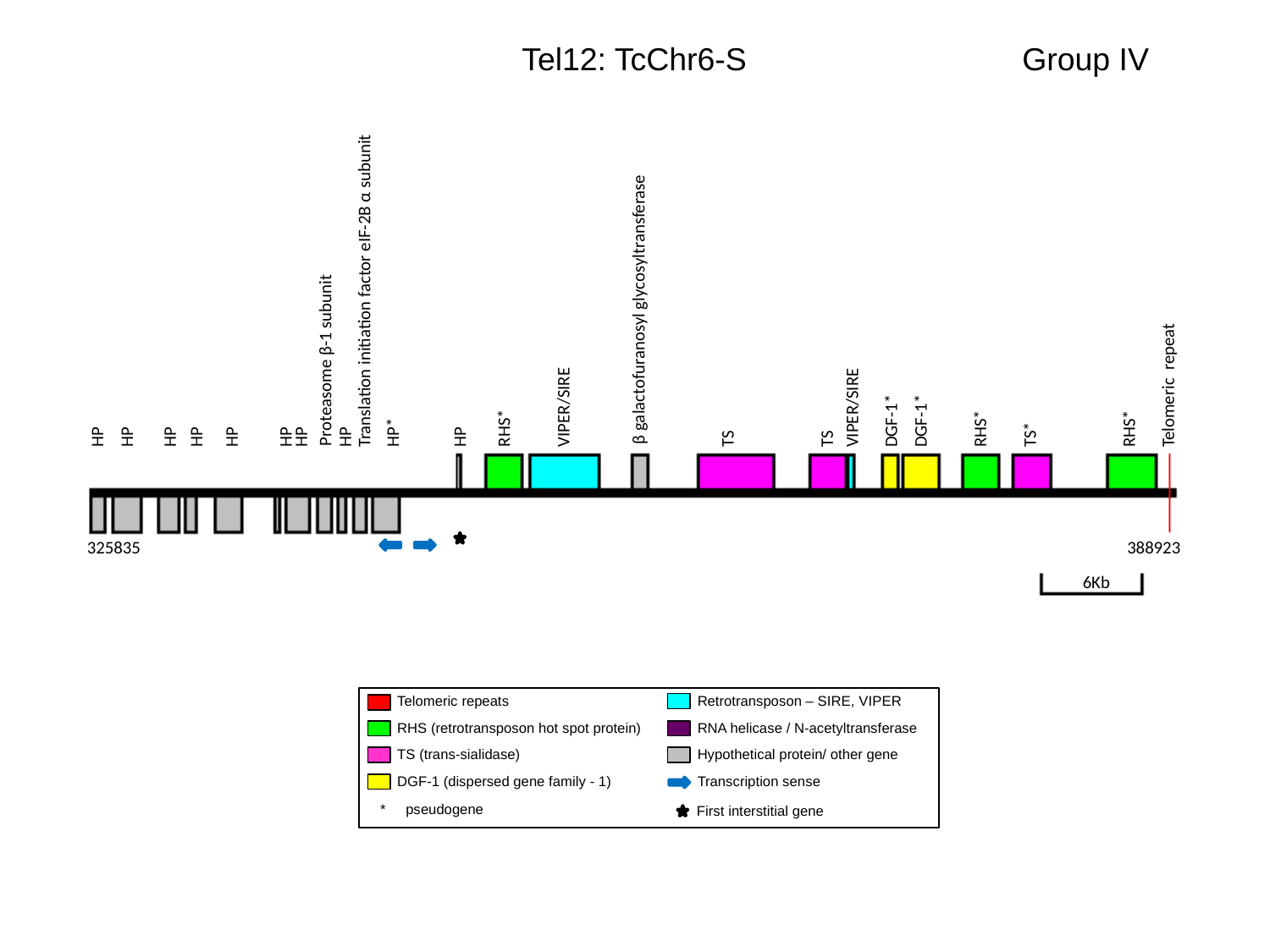

Tel12: TcChr6-S
Group IV
Translation initiation factor eIF-2B α subunit
β galactofuranosyl glycosyltransferase
TS
TS
VIPER/SIRE
DGF-1*
DGF-1*
RHS*
TS*
RHS*
Telomeric repeat
Proteasome β-1 subunit
VIPER/SIRE
RHS*
HP
HP
HP
HP
HP
HP
HP
HP*
HP
HP
325835
388923
6Kb
Telomeric repeats
Retrotransposon – SIRE, VIPER
RHS (retrotransposon hot spot protein)
RNA helicase / N-acetyltransferase
TS (trans-sialidase)
Hypothetical protein/ other gene
DGF-1 (dispersed gene family - 1)
Transcription sense
* pseudogene
First interstitial gene

## Slide 13
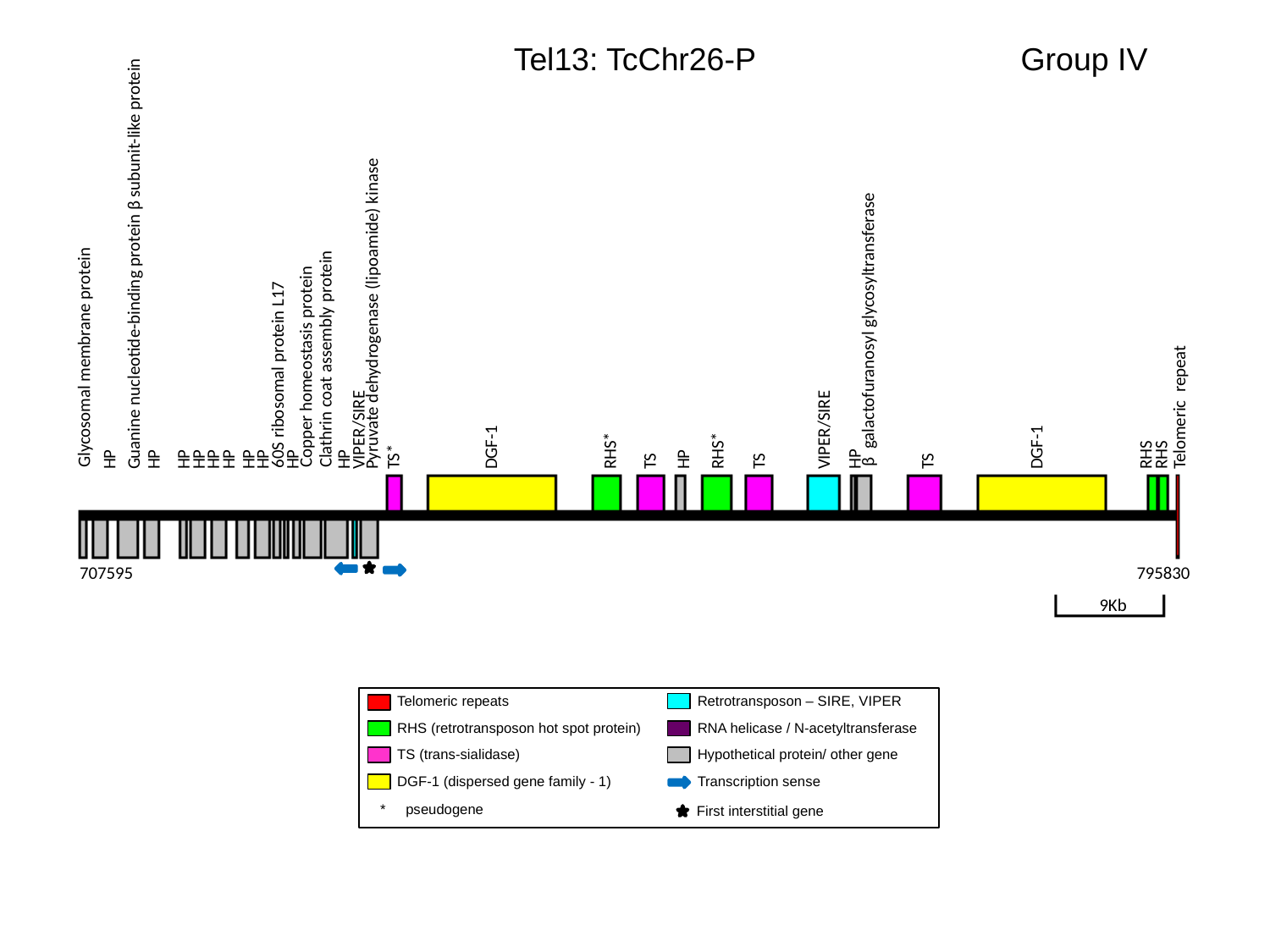

Guanine nucleotide-binding protein β subunit-like protein
Pyruvate dehydrogenase (lipoamide) kinase
β galactofuranosyl glycosyltransferase
Glycosomal membrane protein
Clathrin coat assembly protein
Copper homeostasis protein
HP
HP
HP
HP
HP
HP
HP
HP
HP
HP
VIPER/SIRE
TS*
DGF-1
RHS*
TS
HP
RHS*
TS
VIPER/SIRE
TS
DGF-1
RHS
RHS
Telomeric repeat
60S ribosomal protein L17
HP
707595
795830
9Kb
Tel13: TcChr26-P
Group IV
Telomeric repeats
Retrotransposon – SIRE, VIPER
RHS (retrotransposon hot spot protein)
RNA helicase / N-acetyltransferase
TS (trans-sialidase)
Hypothetical protein/ other gene
DGF-1 (dispersed gene family - 1)
Transcription sense
* pseudogene
First interstitial gene

## Slide 14
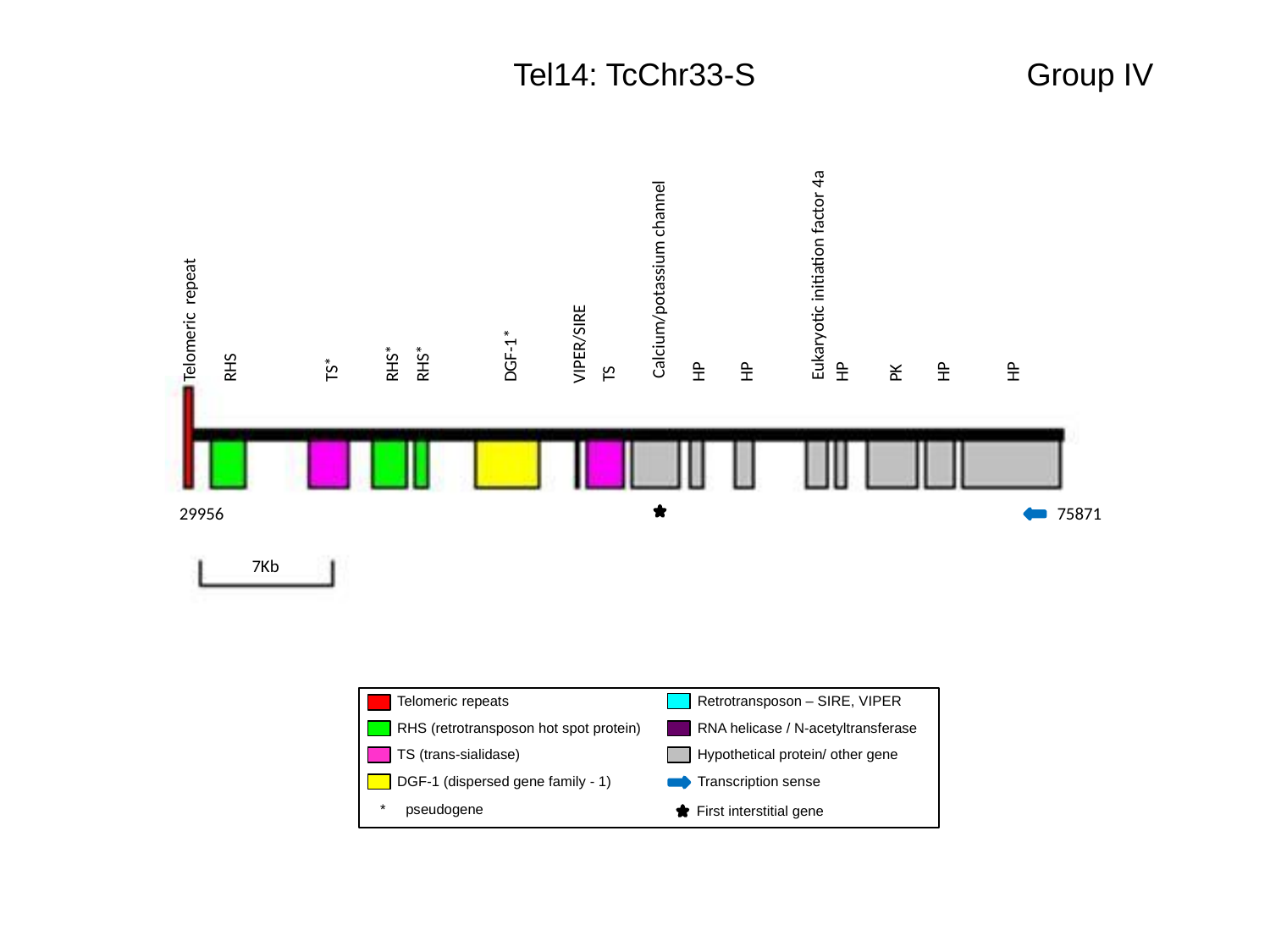

Tel14: TcChr33-S
Group IV
Eukaryotic initiation factor 4a
Calcium/potassium channel
Telomeric repeat
RHS
TS*
RHS*
RHS*
DGF-1*
TS
PK
VIPER/SIRE
HP
HP
HP
HP
HP
29956
75871
7Kb
Telomeric repeats
Retrotransposon – SIRE, VIPER
RHS (retrotransposon hot spot protein)
RNA helicase / N-acetyltransferase
TS (trans-sialidase)
Hypothetical protein/ other gene
DGF-1 (dispersed gene family - 1)
Transcription sense
* pseudogene
First interstitial gene

## Slide 15
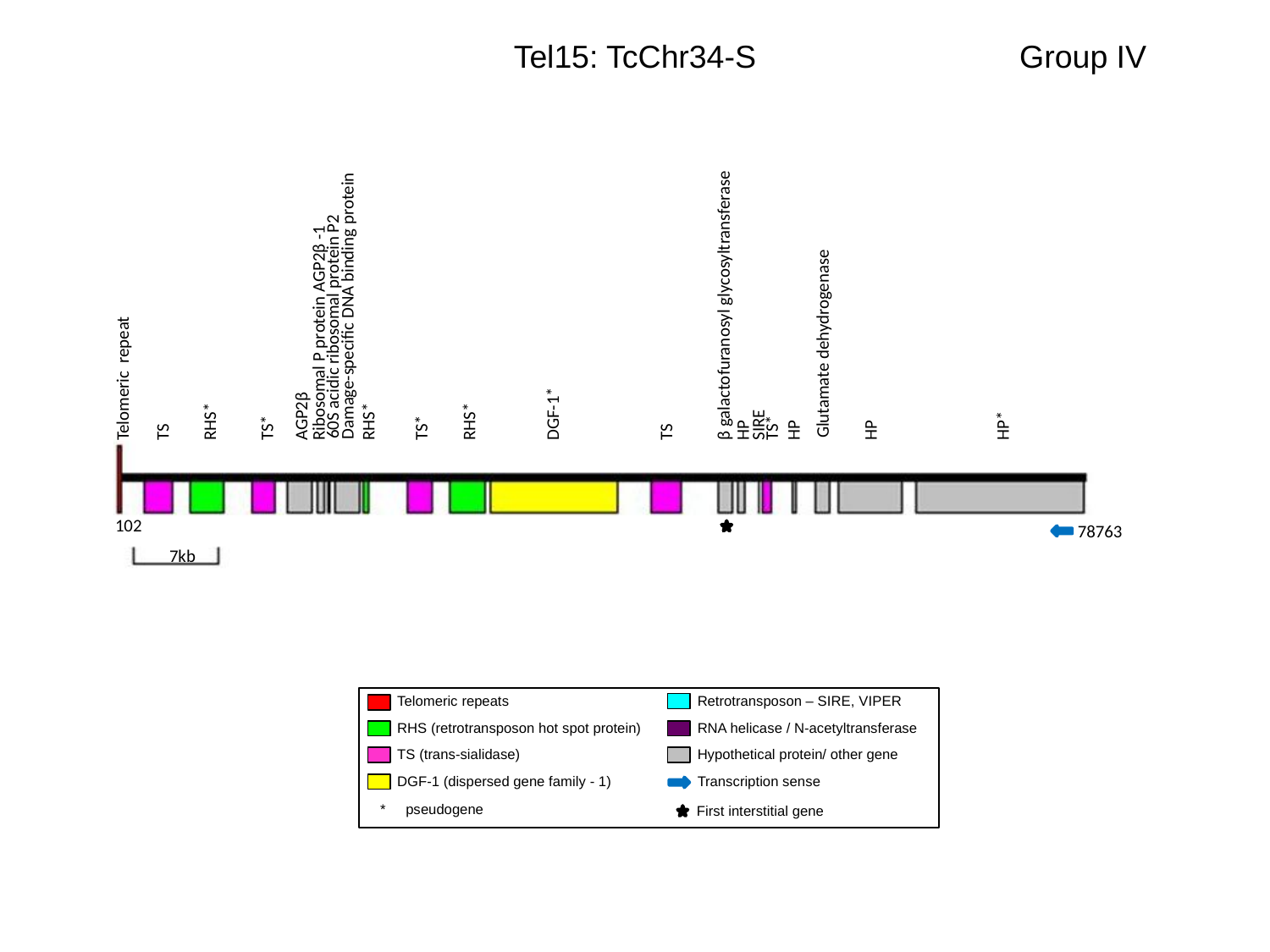

Tel15: TcChr34-S
Group IV
β galactofuranosyl glycosyltransferase
Ribosomal P protein AGP2β -1
Damage-specific DNA binding protein
60S acidic ribosomal protein P2
Telomeric repeat
TS
RHS*
TS*
AGP2β
RHS*
TS*
RHS*
DGF-1*
TS
TS*
Glutamate dehydrogenase
SIRE
HP
HP
HP
HP*
102
78763
7kb
Telomeric repeats
Retrotransposon – SIRE, VIPER
RHS (retrotransposon hot spot protein)
RNA helicase / N-acetyltransferase
TS (trans-sialidase)
Hypothetical protein/ other gene
DGF-1 (dispersed gene family - 1)
Transcription sense
* pseudogene
First interstitial gene

## Slide 16
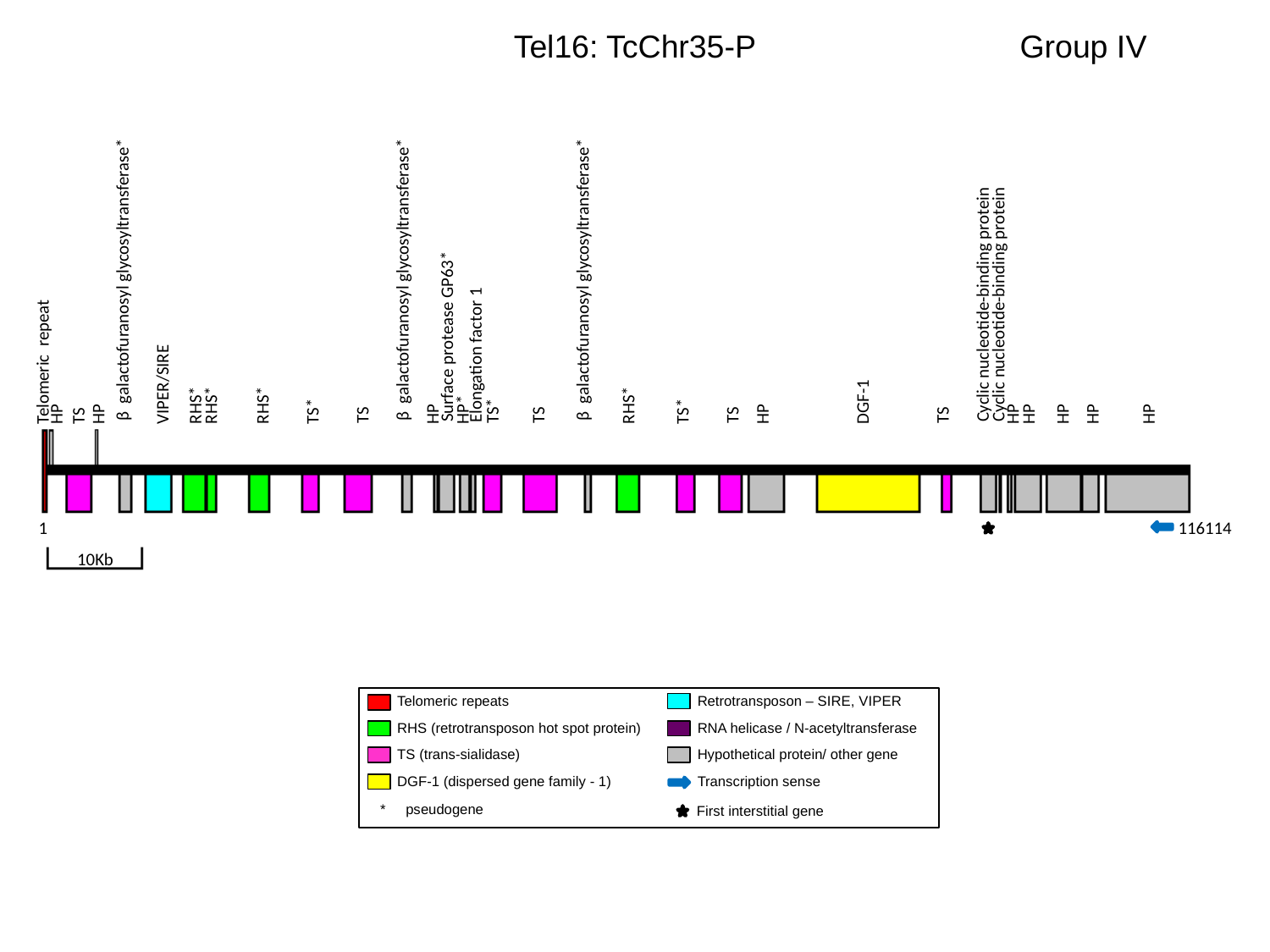

Tel16: TcChr35-P
Group IV
β galactofuranosyl glycosyltransferase*
β galactofuranosyl glycosyltransferase*
β galactofuranosyl glycosyltransferase*
Cyclic nucleotide-binding protein
Cyclic nucleotide-binding protein
Telomeric repeat
HP
TS
HP
VIPER/SIRE
RHS*
RHS*
RHS*
TS*
HP
HP*
RHS*
TS*
HP
DGF-1
HP
HP
HP
HP
HP
Surface protease GP63*
Elongation factor 1
TS*
TS
TS
TS
TS
1
116114
10Kb
Telomeric repeats
Retrotransposon – SIRE, VIPER
RHS (retrotransposon hot spot protein)
RNA helicase / N-acetyltransferase
TS (trans-sialidase)
Hypothetical protein/ other gene
DGF-1 (dispersed gene family - 1)
Transcription sense
* pseudogene
First interstitial gene

## Slide 17
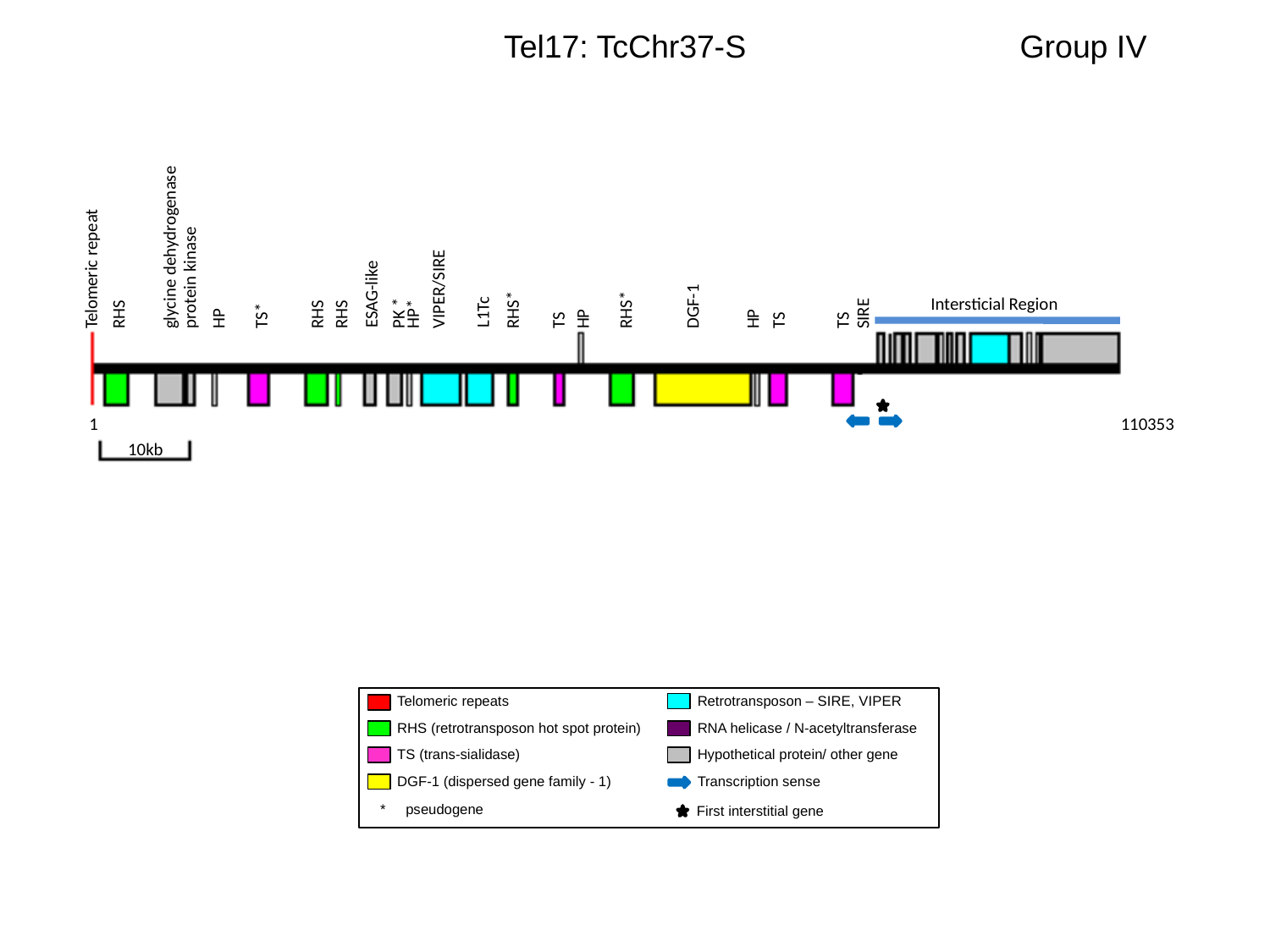

Tel17: TcChr37-S
Group IV
glycine dehydrogenase
Telomeric repeat
protein kinase
SIRE
HP
HP
VIPER/SIRE
ESAG-like
Intersticial Region
RHS
DGF-1
RHS*
RHS*
L1Tc
PK *
HP*
RHS
RHS
TS*
HP
TS
TS
TS
1
110353
10kb
Telomeric repeats
Retrotransposon – SIRE, VIPER
RHS (retrotransposon hot spot protein)
RNA helicase / N-acetyltransferase
TS (trans-sialidase)
Hypothetical protein/ other gene
DGF-1 (dispersed gene family - 1)
Transcription sense
* pseudogene
First interstitial gene

## Slide 18
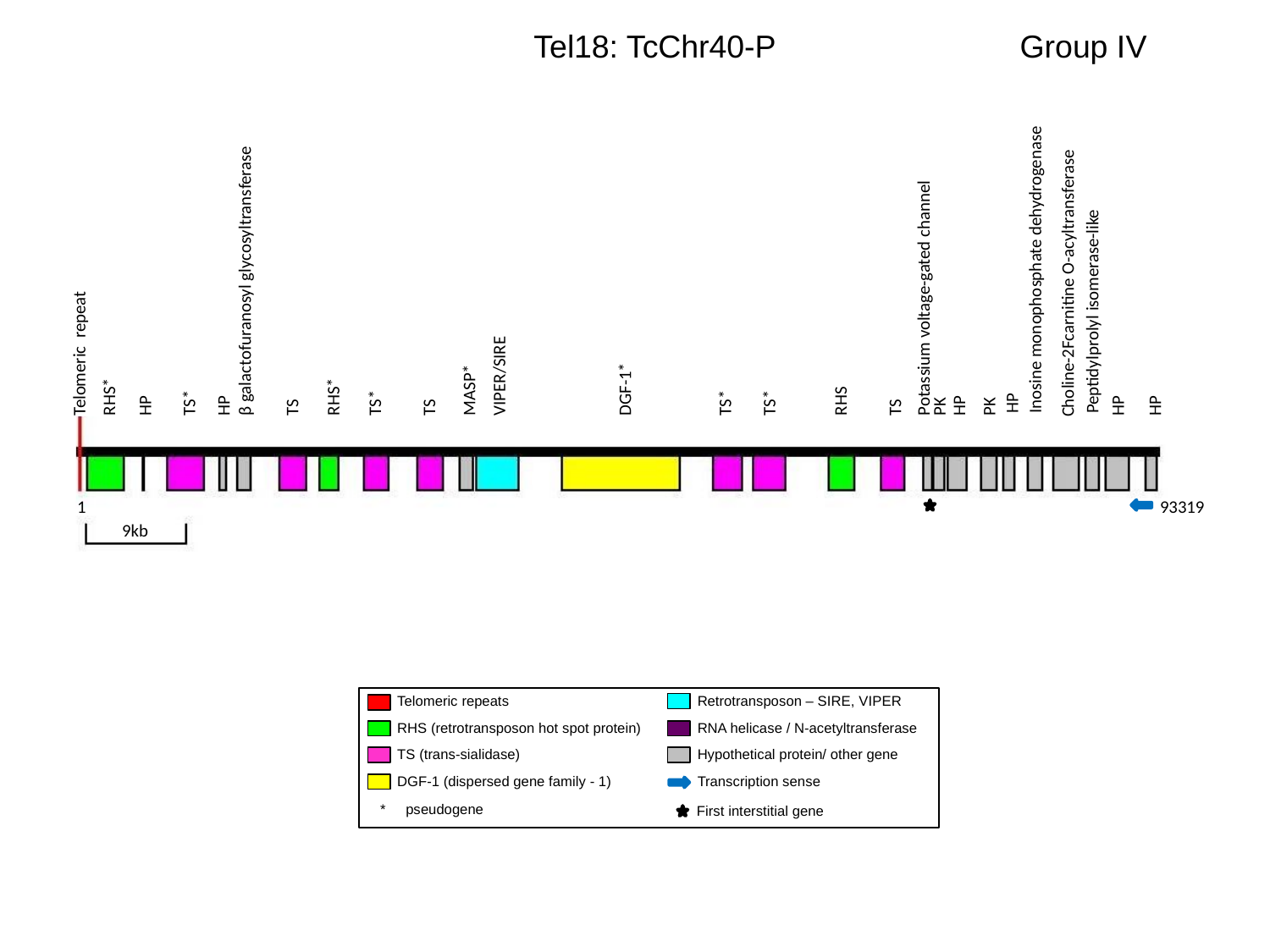

Tel18: TcChr40-P
Group IV
β galactofuranosyl glycosyltransferase
HP
HP
Potassium voltage-gated channel
PK
HP
PK
HP
Inosine monophosphate dehydrogenase
Peptidylprolyl isomerase-like
Telomeric repeat
RHS*
TS*
RHS*
HP
TS
TS*
MASP*
VIPER/SIRE
DGF-1*
TS
TS*
TS
TS*
RHS
HP
1
93319
9kb
Choline-2Fcarnitine O-acyltransferase
Telomeric repeats
Retrotransposon – SIRE, VIPER
RHS (retrotransposon hot spot protein)
RNA helicase / N-acetyltransferase
TS (trans-sialidase)
Hypothetical protein/ other gene
DGF-1 (dispersed gene family - 1)
Transcription sense
* pseudogene
First interstitial gene

## Slide 19
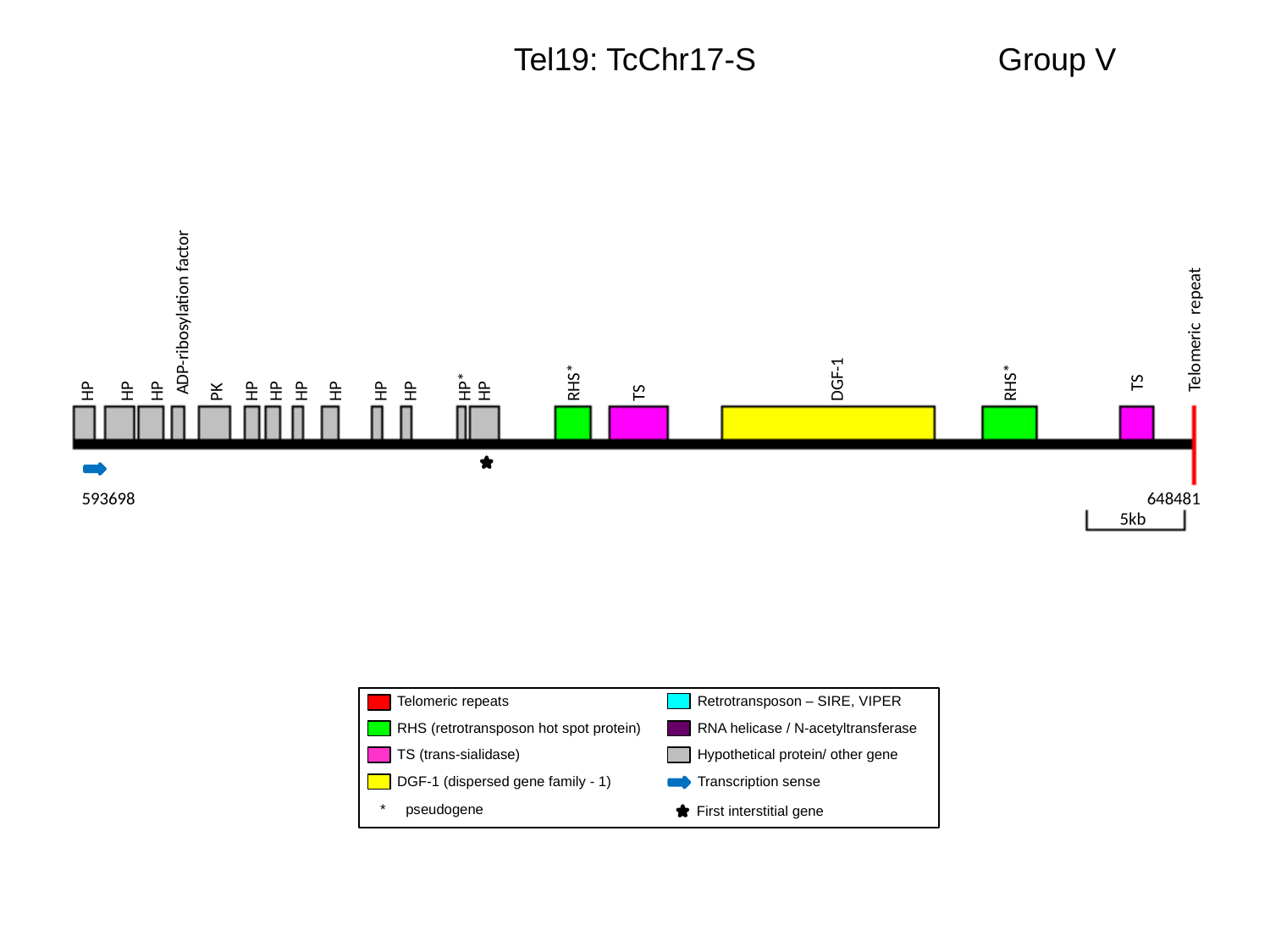

Tel19: TcChr17-S
Group V
Telomeric repeat
ADP-ribosylation factor
TS
HP
HP
HP
PK
HP
HP
HP
HP
HP
HP
HP*
HP
RHS*
TS
DGF-1
RHS*
593698
648481
5kb
Telomeric repeats
Retrotransposon – SIRE, VIPER
RHS (retrotransposon hot spot protein)
RNA helicase / N-acetyltransferase
TS (trans-sialidase)
Hypothetical protein/ other gene
DGF-1 (dispersed gene family - 1)
Transcription sense
* pseudogene
First interstitial gene

## Slide 20
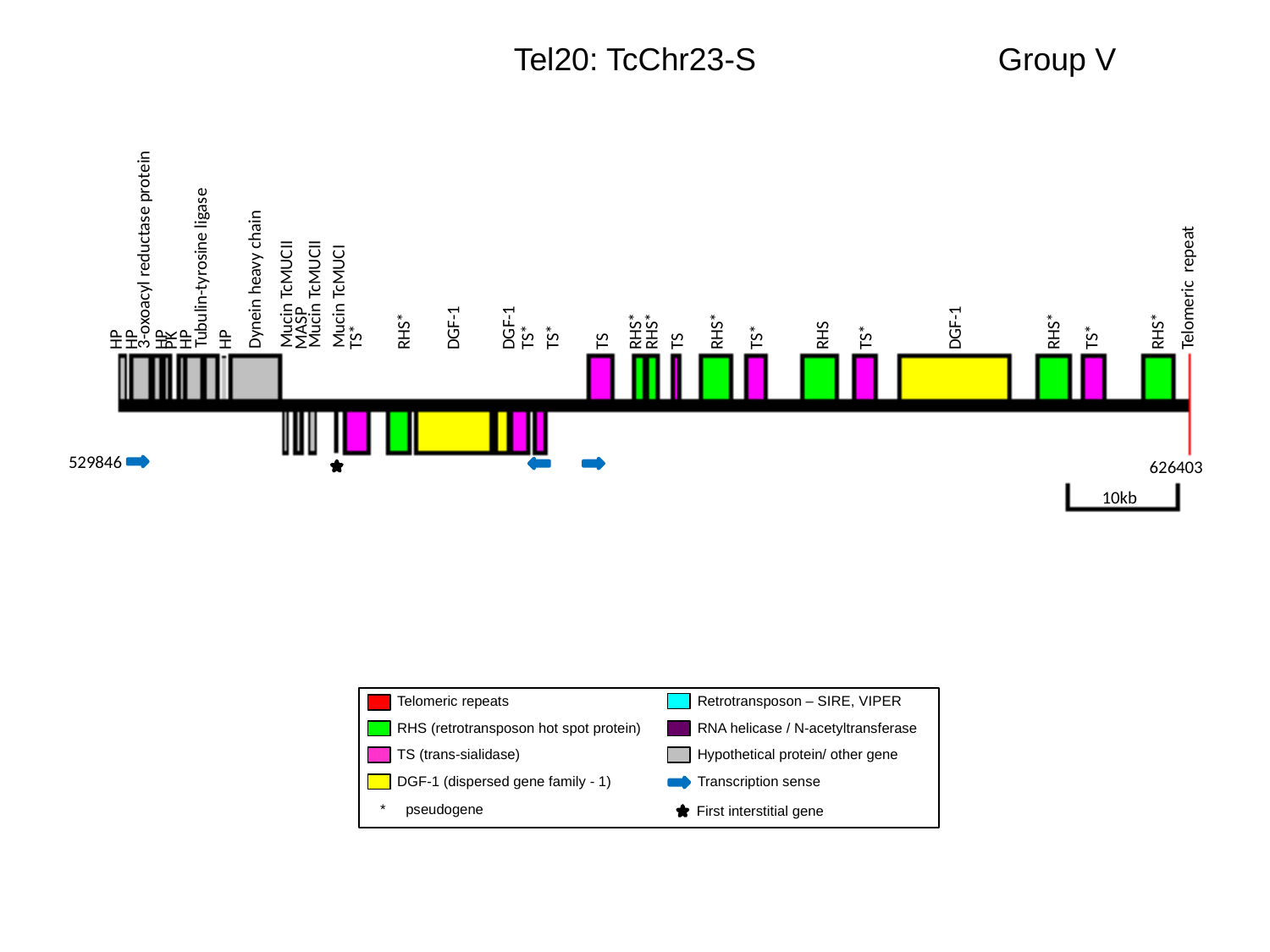

Tel20: TcChr23-S
Group V
3-oxoacyl reductase protein
TS*
TS*
TS*
TS
TS
TS*
TS*
TS*
Telomeric repeat
Tubulin-tyrosine ligase
Dynein heavy chain
Mucin TcMUCII
Mucin TcMUCII
Mucin TcMUCI
RHS*
RHS*
DGF-1
DGF-1
RHS*
RHS*
RHS
DGF-1
RHS*
RHS*
MASP
HP
HP
HP
HP
HP
PK
529846
626403
10kb
Telomeric repeats
Retrotransposon – SIRE, VIPER
RHS (retrotransposon hot spot protein)
RNA helicase / N-acetyltransferase
TS (trans-sialidase)
Hypothetical protein/ other gene
DGF-1 (dispersed gene family - 1)
Transcription sense
* pseudogene
First interstitial gene

## Slide 21
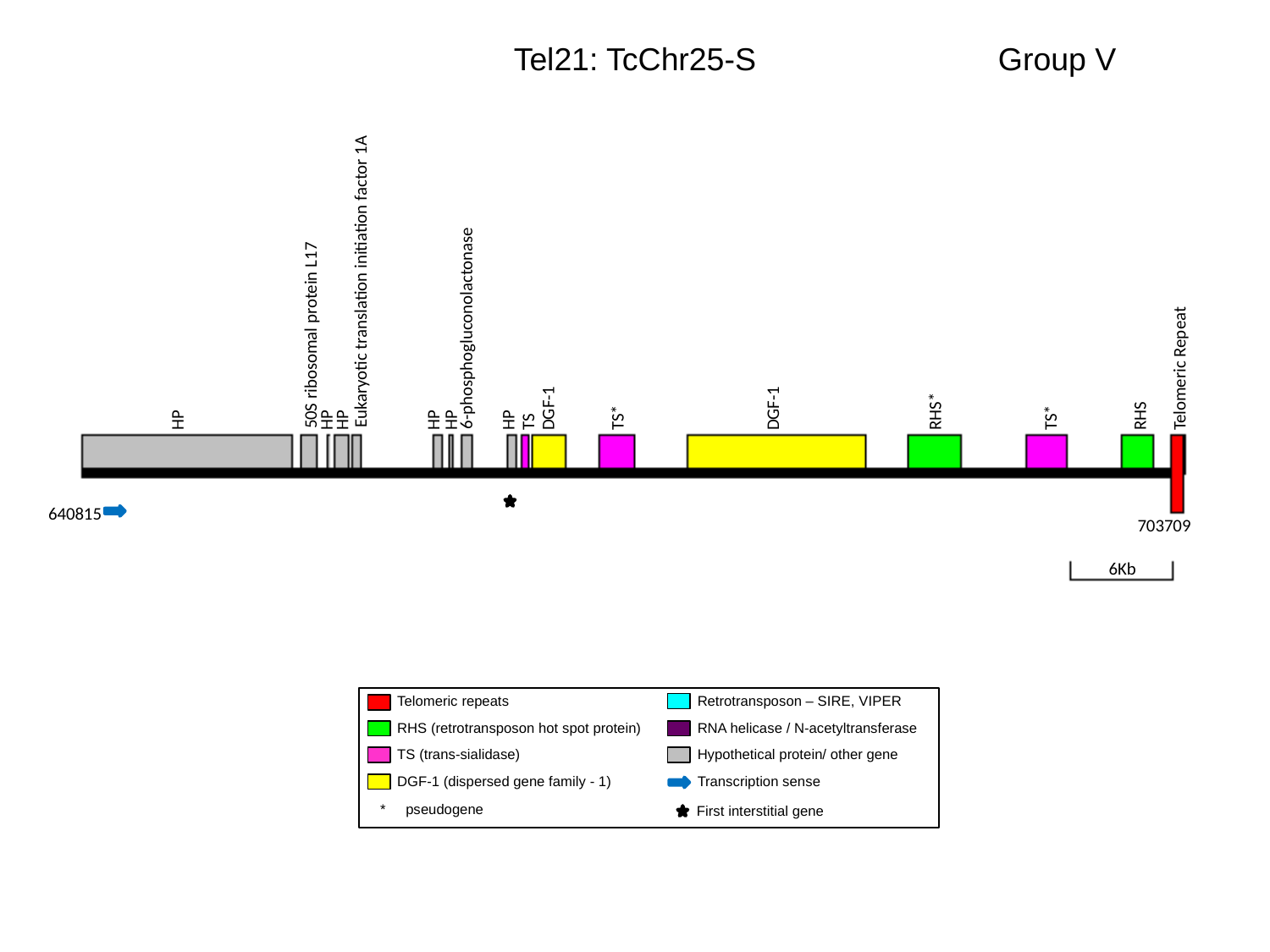

Tel21: TcChr25-S
Group V
Eukaryotic translation initiation factor 1A
6-phosphogluconolactonase
50S ribosomal protein L17
HP
HP
HP
HP
HP
HP
TS
DGF-1
TS*
DGF-1
RHS*
TS*
RHS
Telomeric Repeat
640815
703709
6Kb
Telomeric repeats
Retrotransposon – SIRE, VIPER
RHS (retrotransposon hot spot protein)
RNA helicase / N-acetyltransferase
TS (trans-sialidase)
Hypothetical protein/ other gene
DGF-1 (dispersed gene family - 1)
Transcription sense
* pseudogene
First interstitial gene

## Slide 22
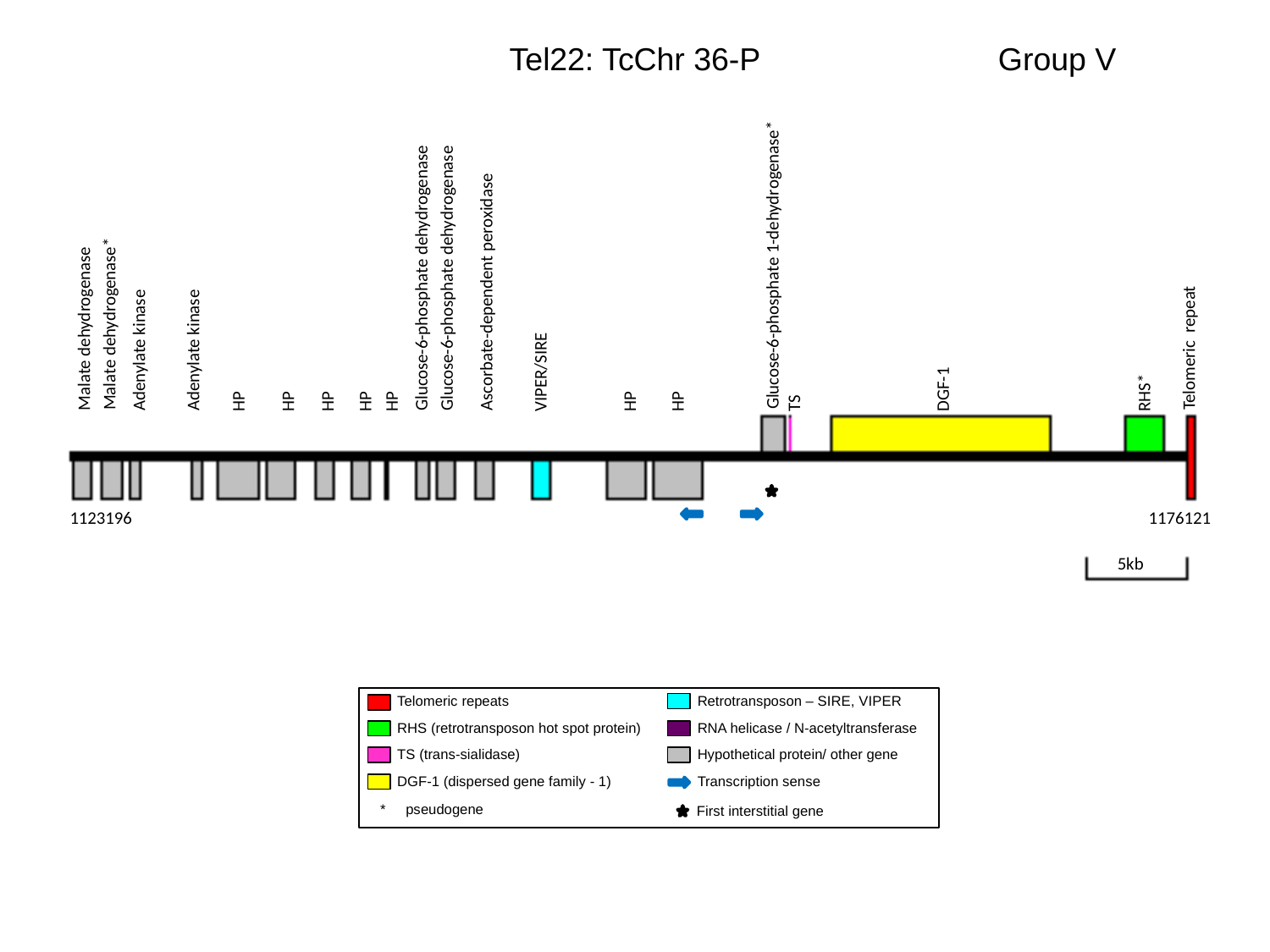

Tel22: TcChr 36-P
Group V
Glucose-6-phosphate 1-dehydrogenase*
Glucose-6-phosphate dehydrogenase
Glucose-6-phosphate dehydrogenase
Ascorbate-dependent peroxidase
Malate dehydrogenase*
Malate dehydrogenase
Telomeric repeat
Adenylate kinase
Adenylate kinase
VIPER/SIRE
DGF-1
RHS*
HP
HP
HP
HP
HP
HP
HP
TS
1123196
1176121
5kb
Telomeric repeats
Retrotransposon – SIRE, VIPER
RHS (retrotransposon hot spot protein)
RNA helicase / N-acetyltransferase
TS (trans-sialidase)
Hypothetical protein/ other gene
DGF-1 (dispersed gene family - 1)
Transcription sense
* pseudogene
First interstitial gene

## Slide 23
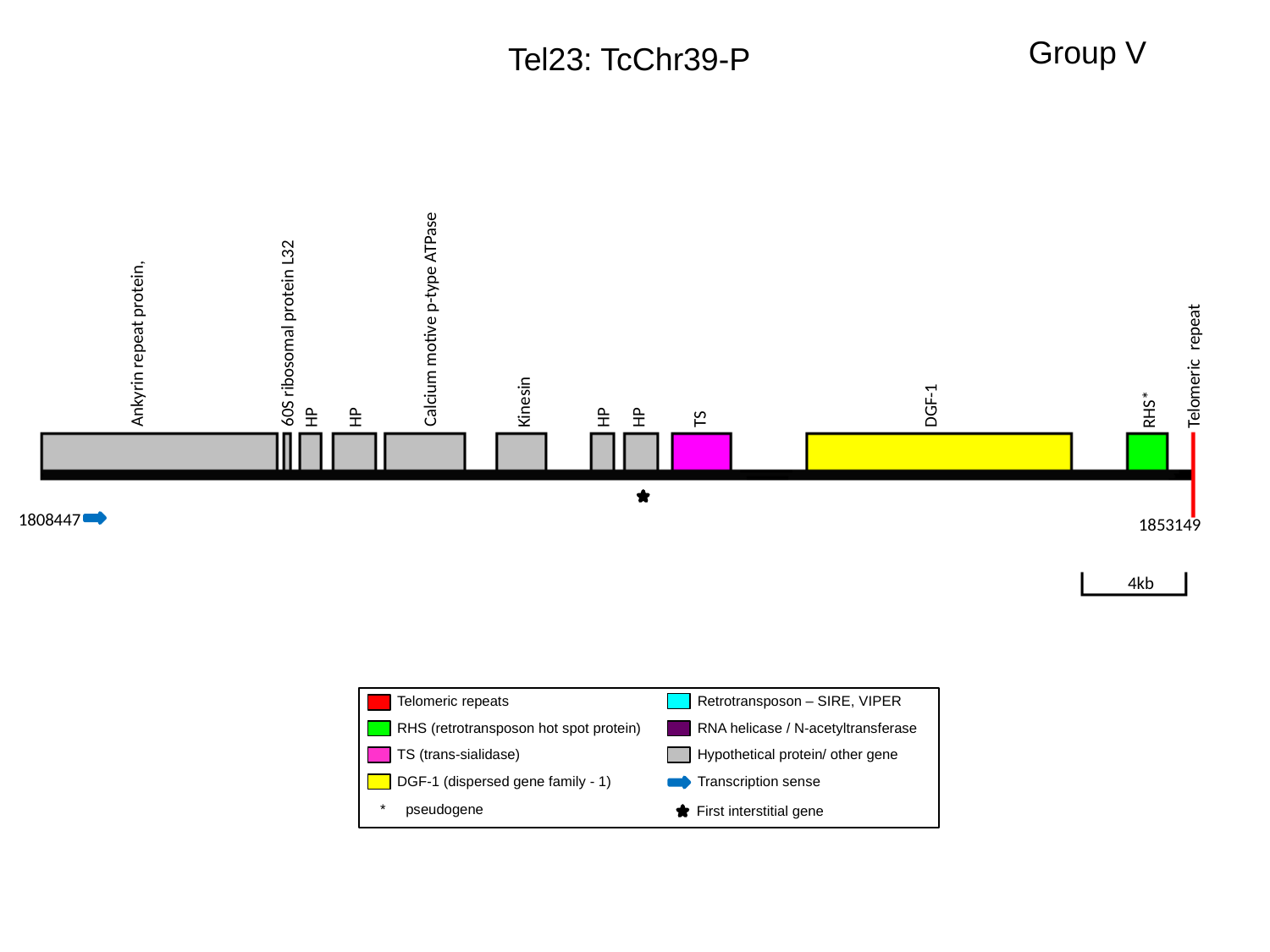

Group V
Tel23: TcChr39-P
Calcium motive p-type ATPase
60S ribosomal protein L32
RHS*
Telomeric repeat
Ankyrin repeat protein,
Kinesin
DGF-1
HP
HP
HP
HP
TS
1808447
1853149
4kb
Telomeric repeats
Retrotransposon – SIRE, VIPER
RHS (retrotransposon hot spot protein)
RNA helicase / N-acetyltransferase
TS (trans-sialidase)
Hypothetical protein/ other gene
DGF-1 (dispersed gene family - 1)
Transcription sense
* pseudogene
First interstitial gene

## Slide 24
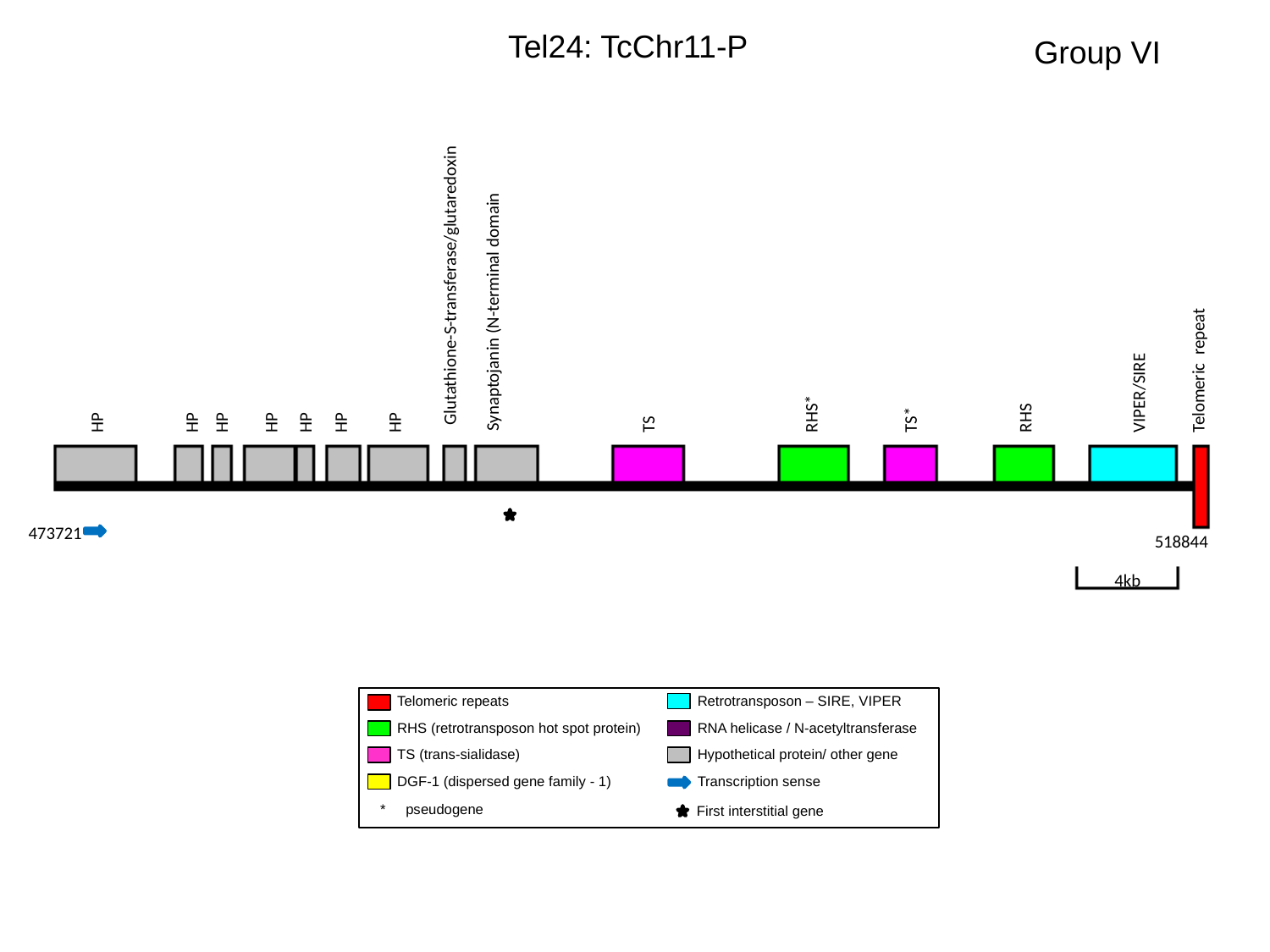

Tel24: TcChr11-P
Group VI
Glutathione-S-transferase/glutaredoxin
Synaptojanin (N-terminal domain
TS
HP
HP
HP
HP
HP
HP
HP
RHS*
TS*
RHS
VIPER/SIRE
Telomeric repeat
473721
518844
4kb
Telomeric repeats
Retrotransposon – SIRE, VIPER
RHS (retrotransposon hot spot protein)
RNA helicase / N-acetyltransferase
TS (trans-sialidase)
Hypothetical protein/ other gene
DGF-1 (dispersed gene family - 1)
Transcription sense
* pseudogene
First interstitial gene

## Slide 25
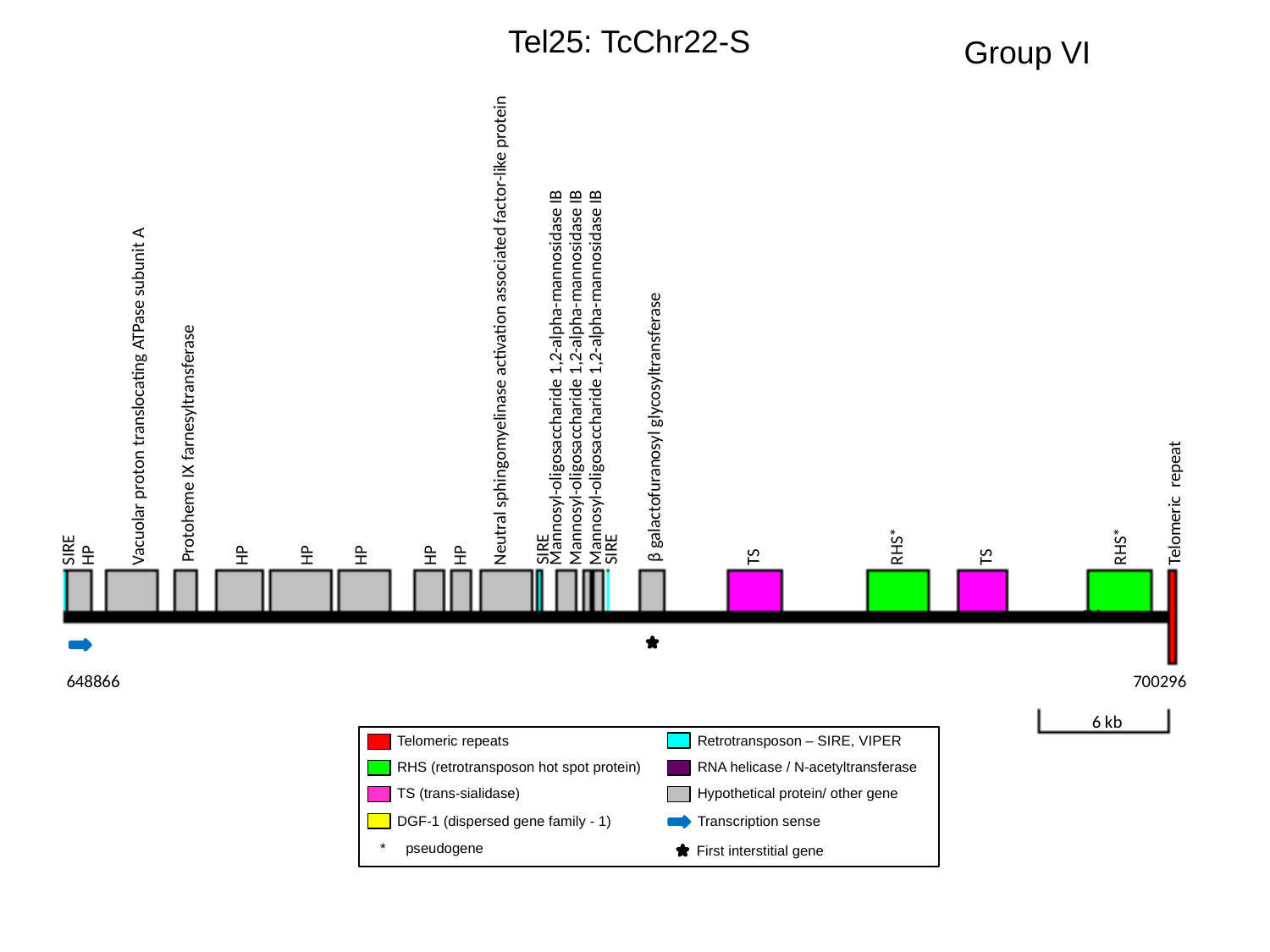

Tel25: TcChr22-S
Group VI
Neutral sphingomyelinase activation associated factor-like protein
Mannosyl-oligosaccharide 1,2-alpha-mannosidase IB
Mannosyl-oligosaccharide 1,2-alpha-mannosidase IB
Mannosyl-oligosaccharide 1,2-alpha-mannosidase IB
Vacuolar proton translocating ATPase subunit A
β galactofuranosyl glycosyltransferase
Protoheme IX farnesyltransferase
TS
RHS*
TS
RHS*
Telomeric repeat
SIRE
HP
HP
HP
HP
HP
HP
SIRE
SIRE
6kb
648866
700296
6 kb
Telomeric repeats
Retrotransposon – SIRE, VIPER
RHS (retrotransposon hot spot protein)
RNA helicase / N-acetyltransferase
TS (trans-sialidase)
Hypothetical protein/ other gene
DGF-1 (dispersed gene family - 1)
Transcription sense
* pseudogene
First interstitial gene

## Slide 26
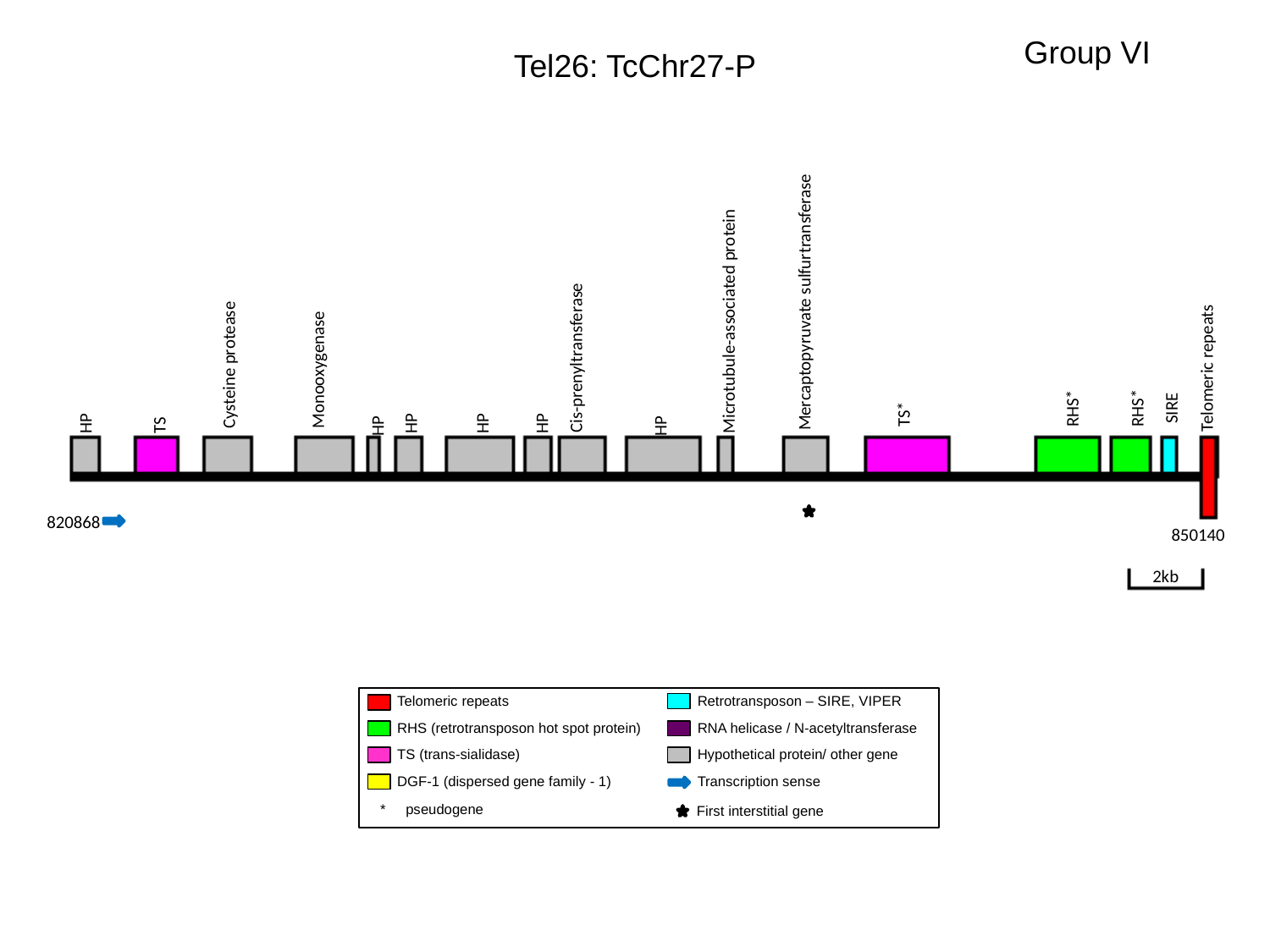

Group VI
Tel26: TcChr27-P
Mercaptopyruvate sulfurtransferase
Microtubule-associated protein
Cis-prenyltransferase
Monooxygenase
Cysteine protease
Telomeric repeats
HP
HP
HP
HP
HP
RHS*
SIRE
RHS*
TS*
HP
TS
820868
850140
2kb
Telomeric repeats
Retrotransposon – SIRE, VIPER
RHS (retrotransposon hot spot protein)
RNA helicase / N-acetyltransferase
TS (trans-sialidase)
Hypothetical protein/ other gene
DGF-1 (dispersed gene family - 1)
Transcription sense
* pseudogene
First interstitial gene

## Slide 27
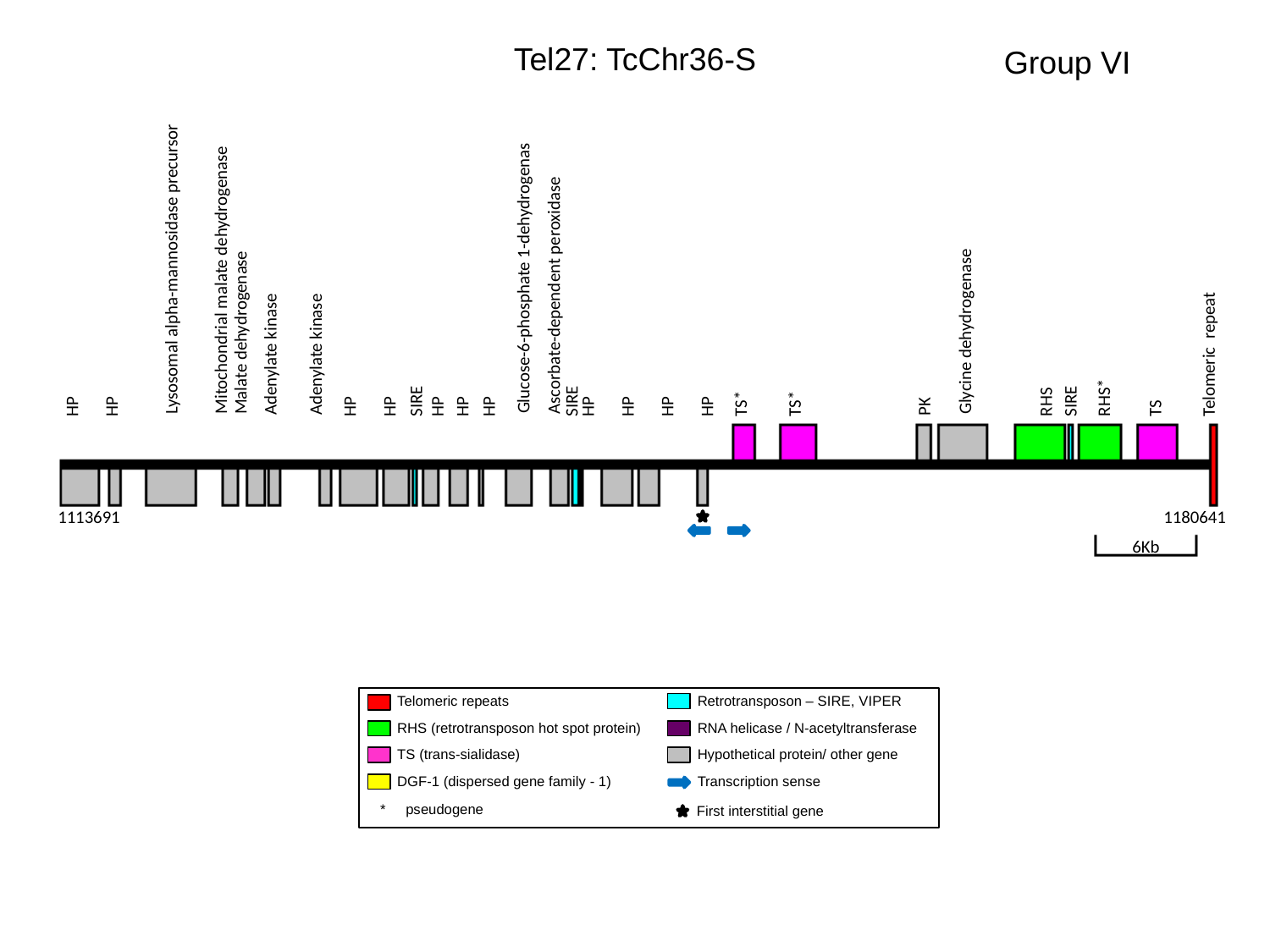

Tel27: TcChr36-S
Group VI
Lysosomal alpha-mannosidase precursor
Glucose-6-phosphate 1-dehydrogenas
Mitochondrial malate dehydrogenase
Ascorbate-dependent peroxidase
HP
HP
HP
HP
SIRE
HP
HP
HP
SIRE
HP
HP
HP
HP
TS*
TS*
RHS
SIRE
RHS*
TS
Telomeric repeat
Glycine dehydrogenase
Malate dehydrogenase
Adenylate kinase
Adenylate kinase
PK
1113691
1180641
6Kb
Telomeric repeats
Retrotransposon – SIRE, VIPER
RHS (retrotransposon hot spot protein)
RNA helicase / N-acetyltransferase
TS (trans-sialidase)
Hypothetical protein/ other gene
DGF-1 (dispersed gene family - 1)
Transcription sense
* pseudogene
First interstitial gene

## Slide 28
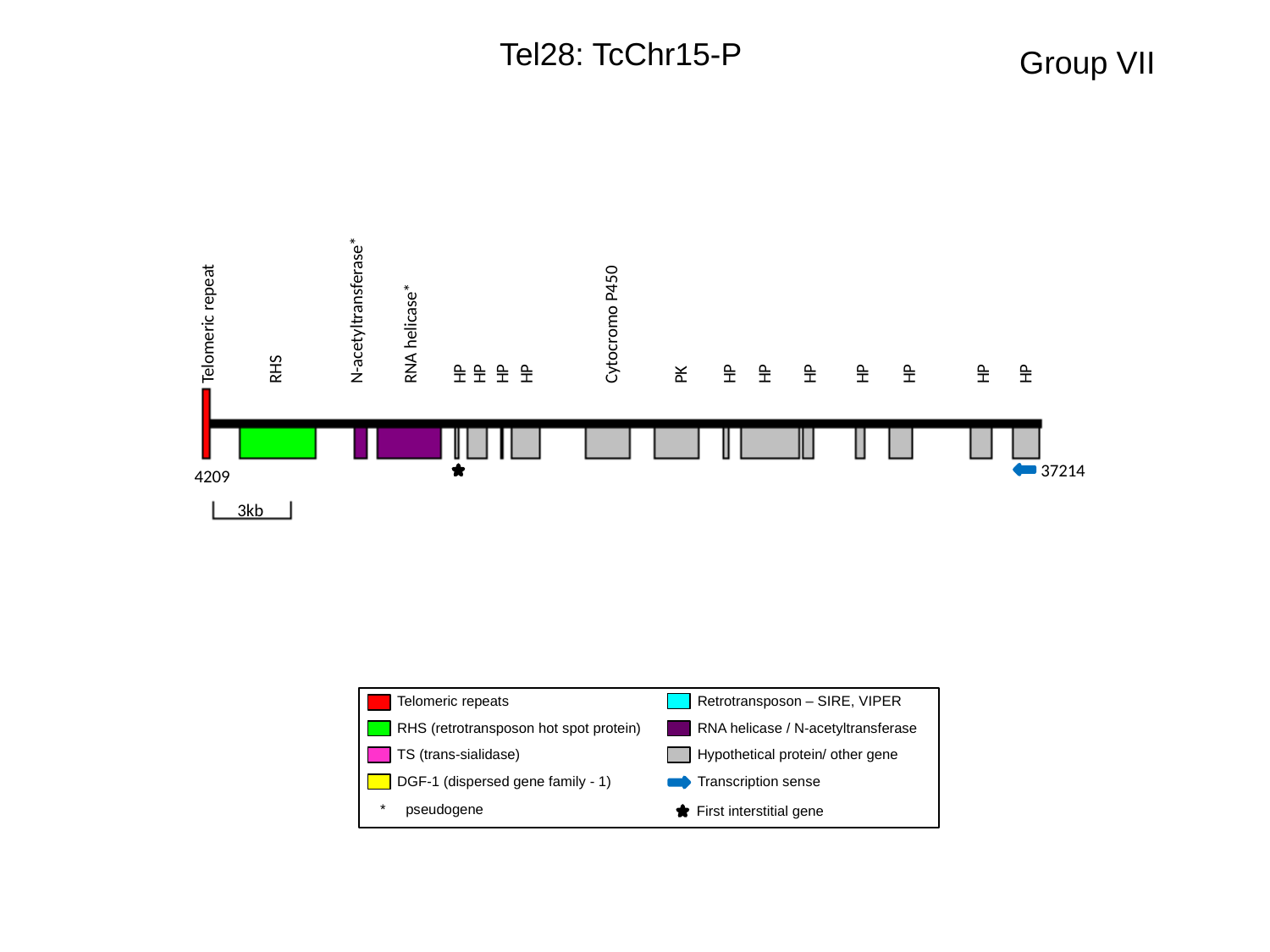

Tel28: TcChr15-P
Group VII
RHS
N-acetyltransferase*
RNA helicase*
HP
HP
HP
HP
Cytocromo P450
PK
HP
HP
HP
HP
HP
HP
HP
Telomeric repeat
37214
4209
3kb
Telomeric repeats
Retrotransposon – SIRE, VIPER
RHS (retrotransposon hot spot protein)
RNA helicase / N-acetyltransferase
TS (trans-sialidase)
Hypothetical protein/ other gene
DGF-1 (dispersed gene family - 1)
Transcription sense
* pseudogene
First interstitial gene

## Slide 29
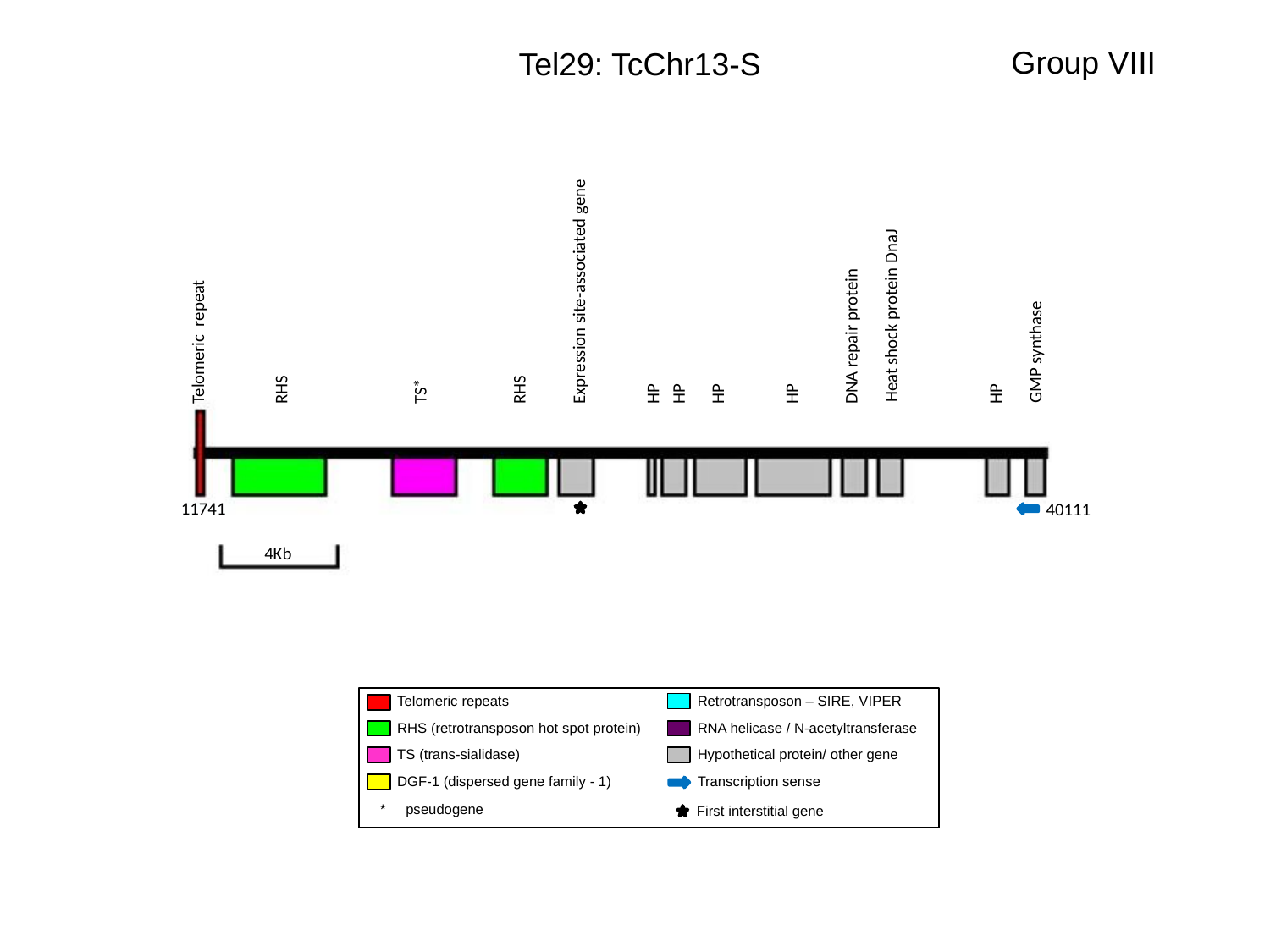

Group VIII
Tel29: TcChr13-S
Expression site-associated gene
Telomeric repeat
Heat shock protein DnaJ
DNA repair protein
HP
HP
GMP synthase
HP
HP
HP
RHS
TS*
RHS
11741
40111
4Kb
Telomeric repeats
Retrotransposon – SIRE, VIPER
RHS (retrotransposon hot spot protein)
RNA helicase / N-acetyltransferase
TS (trans-sialidase)
Hypothetical protein/ other gene
DGF-1 (dispersed gene family - 1)
Transcription sense
* pseudogene
First interstitial gene

## Slide 30
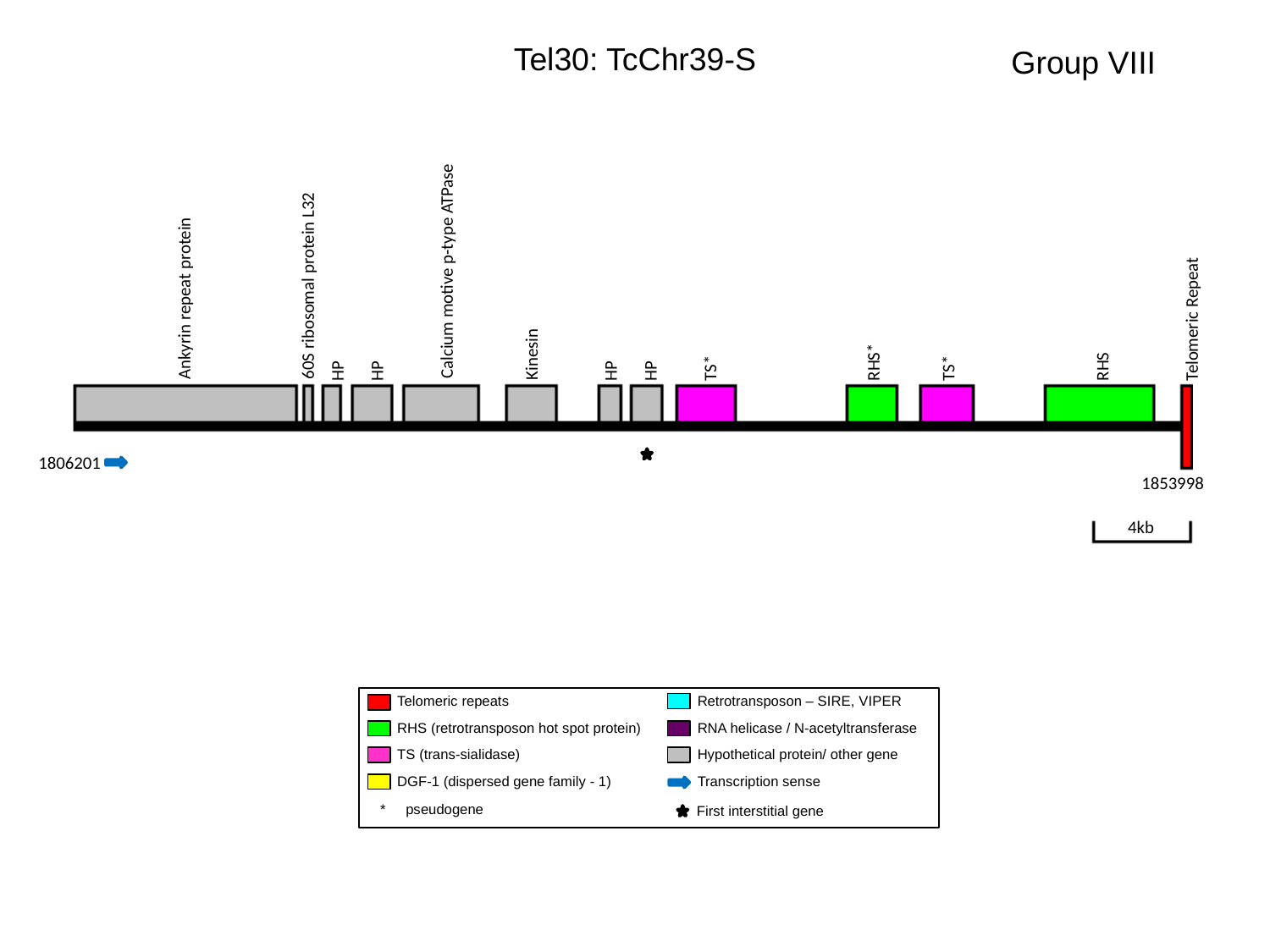

Tel30: TcChr39-S
Group VIII
Calcium motive p-type ATPase
60S ribosomal protein L32
HP
HP
HP
HP
TS*
RHS*
TS*
RHS
Telomeric Repeat
Ankyrin repeat protein
Kinesin
1806201
1853998
4kb
Telomeric repeats
Retrotransposon – SIRE, VIPER
RHS (retrotransposon hot spot protein)
RNA helicase / N-acetyltransferase
TS (trans-sialidase)
Hypothetical protein/ other gene
DGF-1 (dispersed gene family - 1)
Transcription sense
* pseudogene
First interstitial gene

## Slide 31
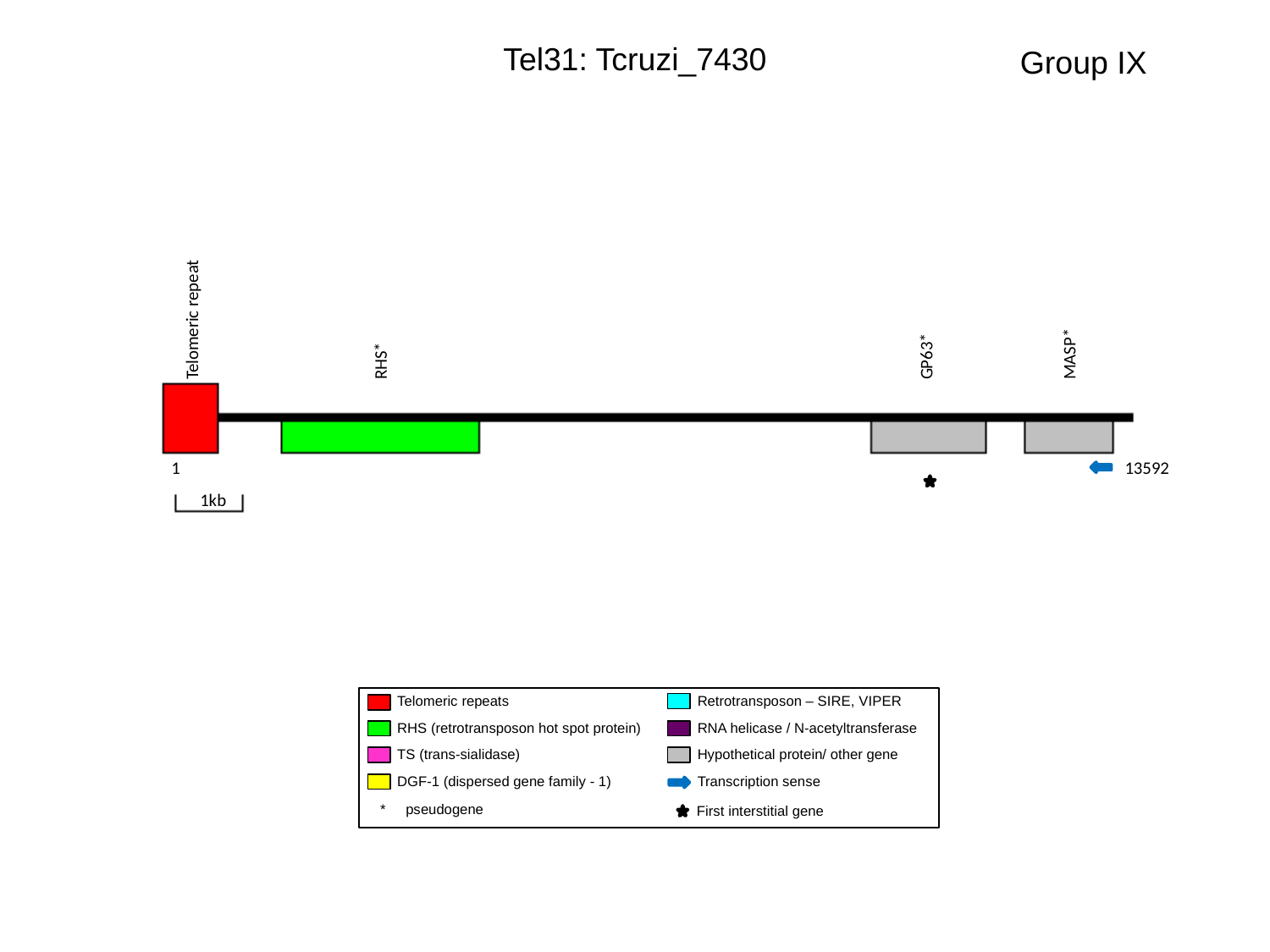

Tel31: Tcruzi_7430
Group IX
Telomeric repeat
RHS*
GP63*
MASP*
13592
1
1kb
Telomeric repeats
Retrotransposon – SIRE, VIPER
RHS (retrotransposon hot spot protein)
RNA helicase / N-acetyltransferase
TS (trans-sialidase)
Hypothetical protein/ other gene
DGF-1 (dispersed gene family - 1)
Transcription sense
* pseudogene
First interstitial gene

## Slide 32
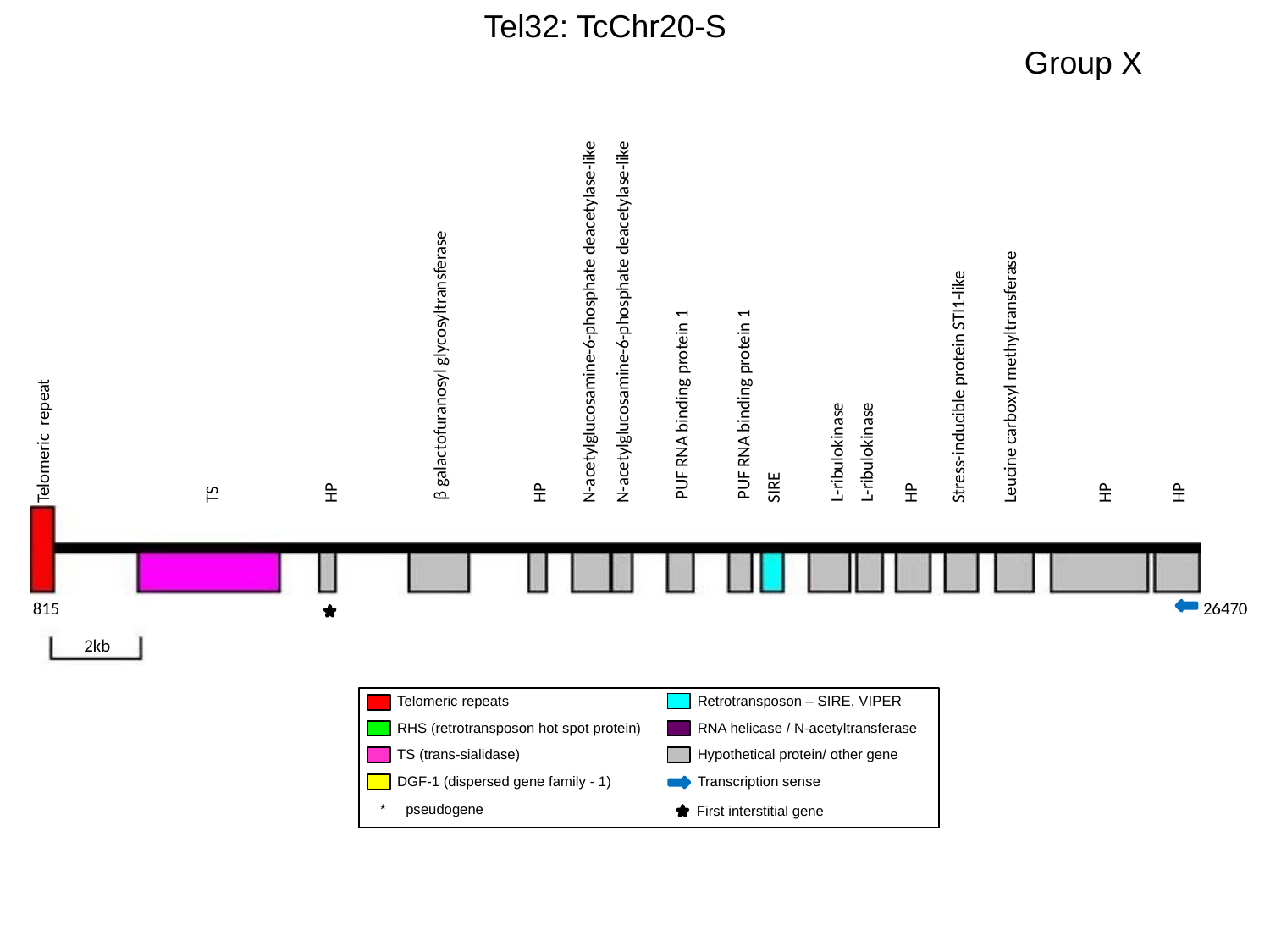

Tel32: TcChr20-S
Group X
N-acetylglucosamine-6-phosphate deacetylase-like
N-acetylglucosamine-6-phosphate deacetylase-like
Leucine carboxyl methyltransferase
Stress-inducible protein STI1-like
β galactofuranosyl glycosyltransferase
PUF RNA binding protein 1
PUF RNA binding protein 1
HP
Telomeric repeat
TS
HP
HP
HP
HP
L-ribulokinase
L-ribulokinase
SIRE
2kb
815
26470
2kb
Telomeric repeats
Retrotransposon – SIRE, VIPER
RHS (retrotransposon hot spot protein)
RNA helicase / N-acetyltransferase
TS (trans-sialidase)
Hypothetical protein/ other gene
DGF-1 (dispersed gene family - 1)
Transcription sense
* pseudogene
First interstitial gene

## Slide 33
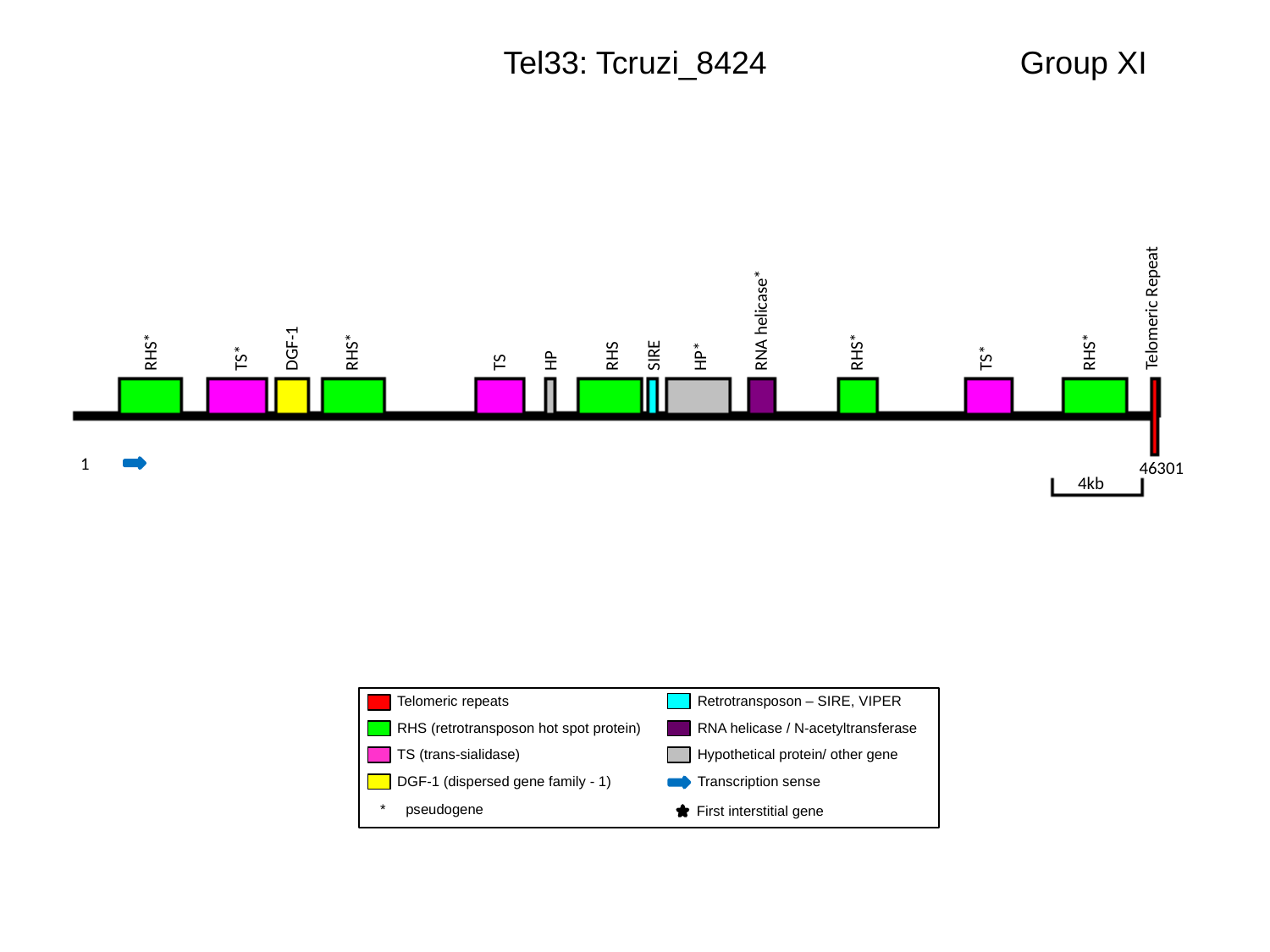

Tel33: Tcruzi_8424
Group XI
Telomeric Repeat
HP
RHS
SIRE
RHS*
TS*
DGF-1
RHS*
HP*
RNA helicase*
RHS*
TS*
RHS*
TS
1
46301
4kb
Telomeric repeats
Retrotransposon – SIRE, VIPER
RHS (retrotransposon hot spot protein)
RNA helicase / N-acetyltransferase
TS (trans-sialidase)
Hypothetical protein/ other gene
DGF-1 (dispersed gene family - 1)
Transcription sense
* pseudogene
First interstitial gene

## Slide 34
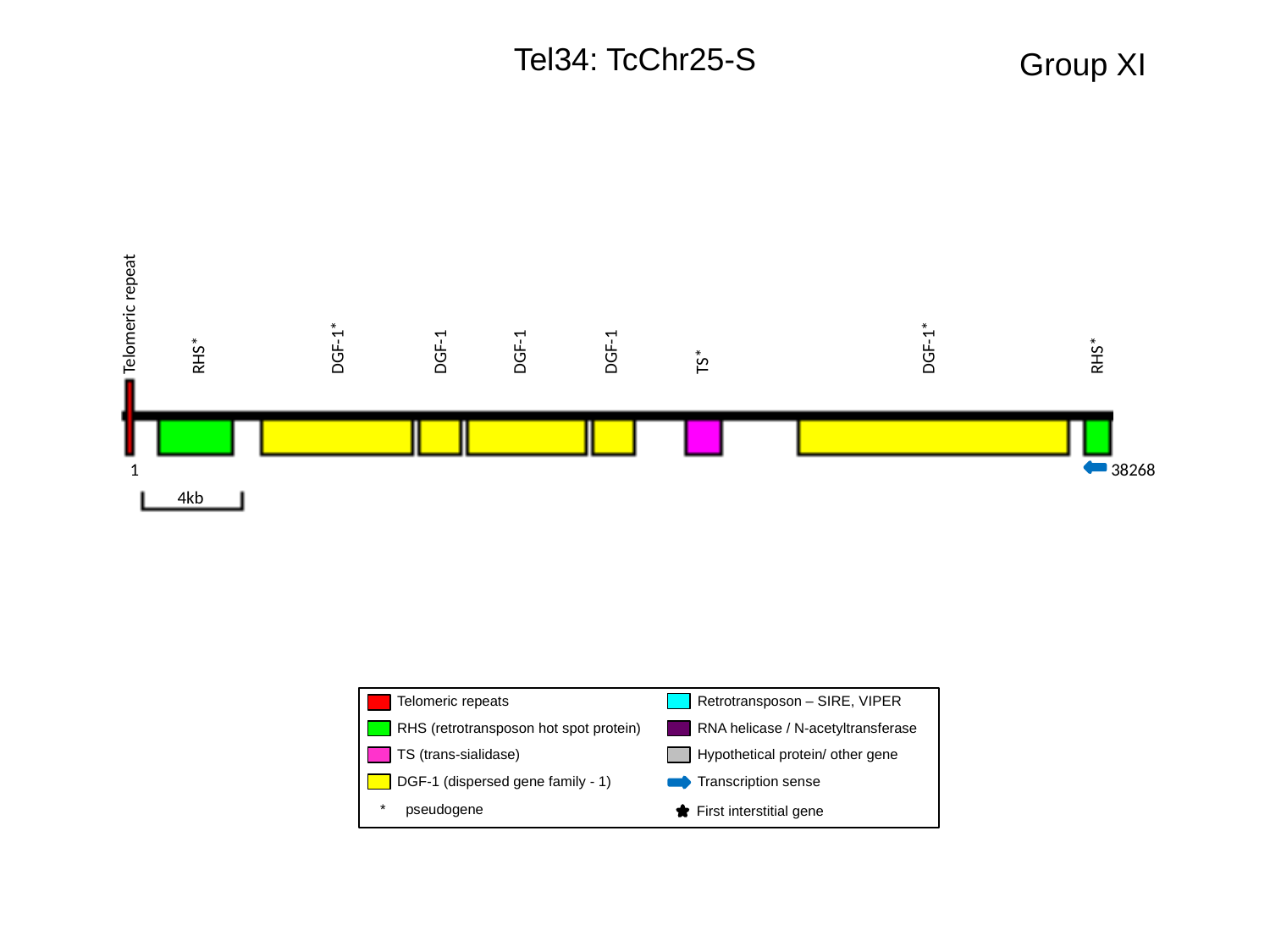

Tel34: TcChr25-S
Group XI
Telomeric repeat
RHS*
DGF-1*
DGF-1
DGF-1
DGF-1
TS*
DGF-1*
RHS*
38268
1
4kb
Telomeric repeats
Retrotransposon – SIRE, VIPER
RHS (retrotransposon hot spot protein)
RNA helicase / N-acetyltransferase
TS (trans-sialidase)
Hypothetical protein/ other gene
DGF-1 (dispersed gene family - 1)
Transcription sense
* pseudogene
First interstitial gene

## Slide 35
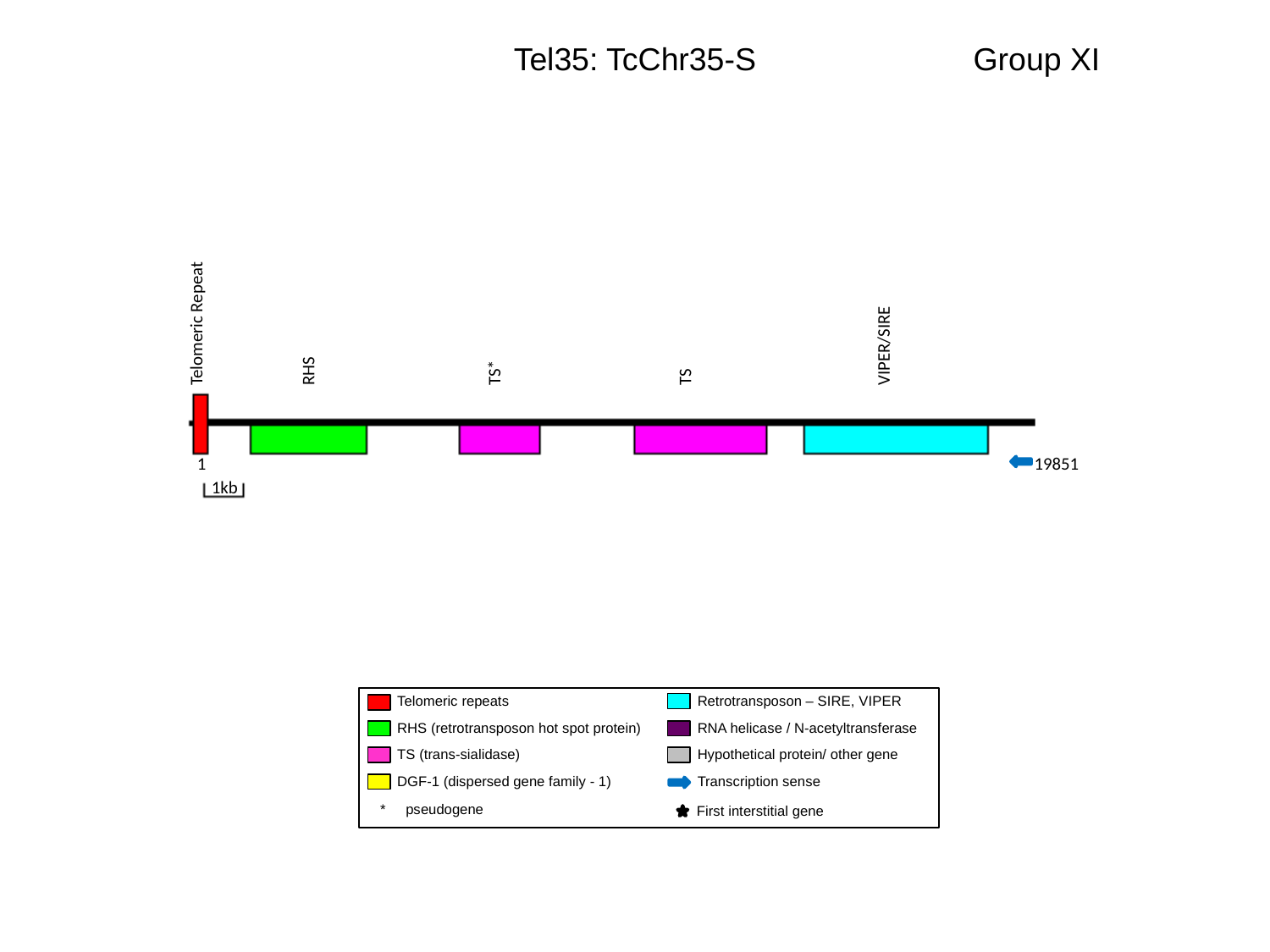

Tel35: TcChr35-S
Group XI
Telomeric Repeat
RHS
TS*
TS
VIPER/SIRE
1
19851
1kb
Telomeric repeats
Retrotransposon – SIRE, VIPER
RHS (retrotransposon hot spot protein)
RNA helicase / N-acetyltransferase
TS (trans-sialidase)
Hypothetical protein/ other gene
DGF-1 (dispersed gene family - 1)
Transcription sense
* pseudogene
First interstitial gene

## Slide 36
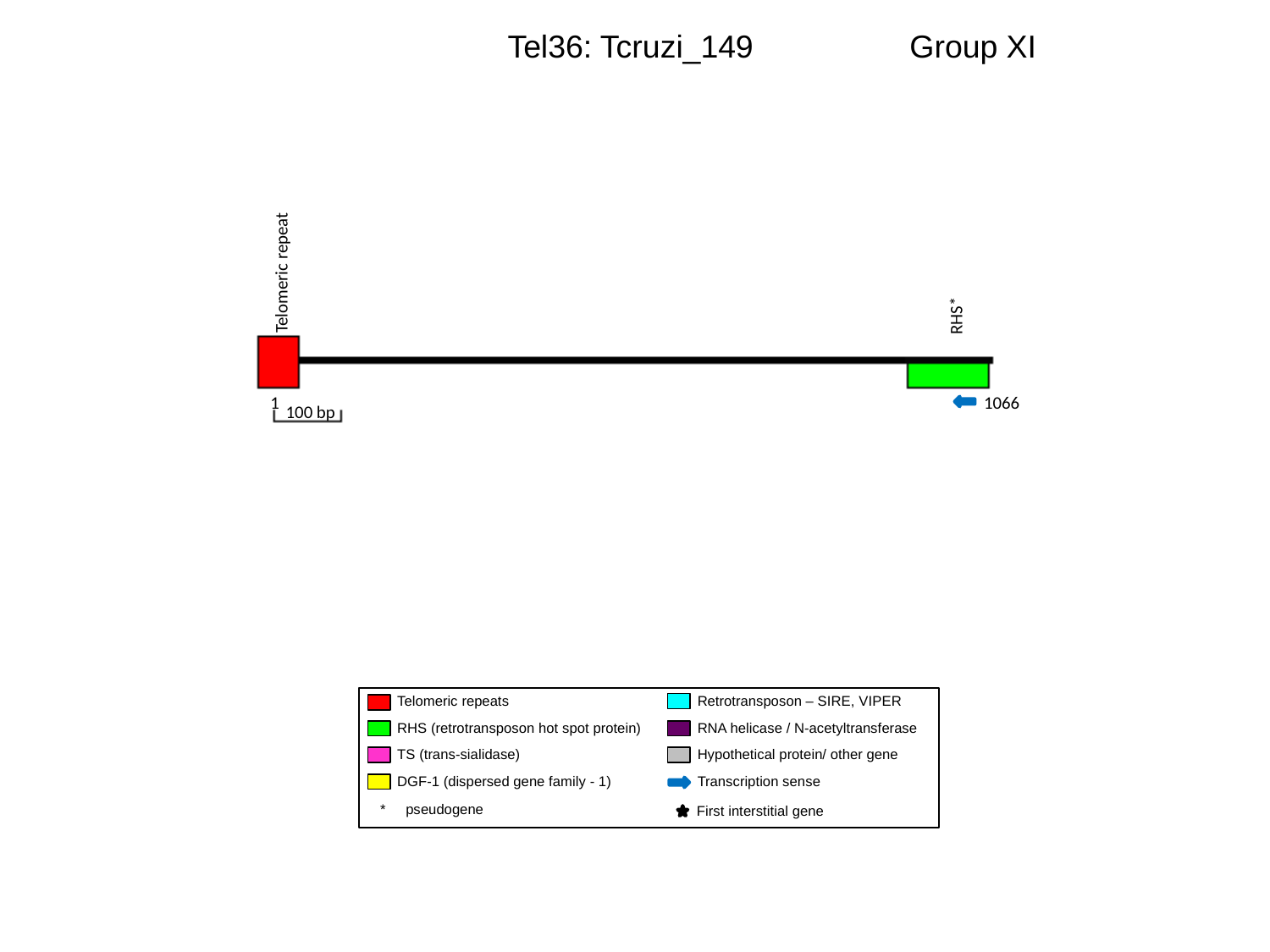

Tel36: Tcruzi_149
Group XI
Telomeric repeat
RHS*
1
1066
100 bp
Telomeric repeats
Retrotransposon – SIRE, VIPER
RHS (retrotransposon hot spot protein)
RNA helicase / N-acetyltransferase
TS (trans-sialidase)
Hypothetical protein/ other gene
DGF-1 (dispersed gene family - 1)
Transcription sense
* pseudogene
First interstitial gene

## Slide 37
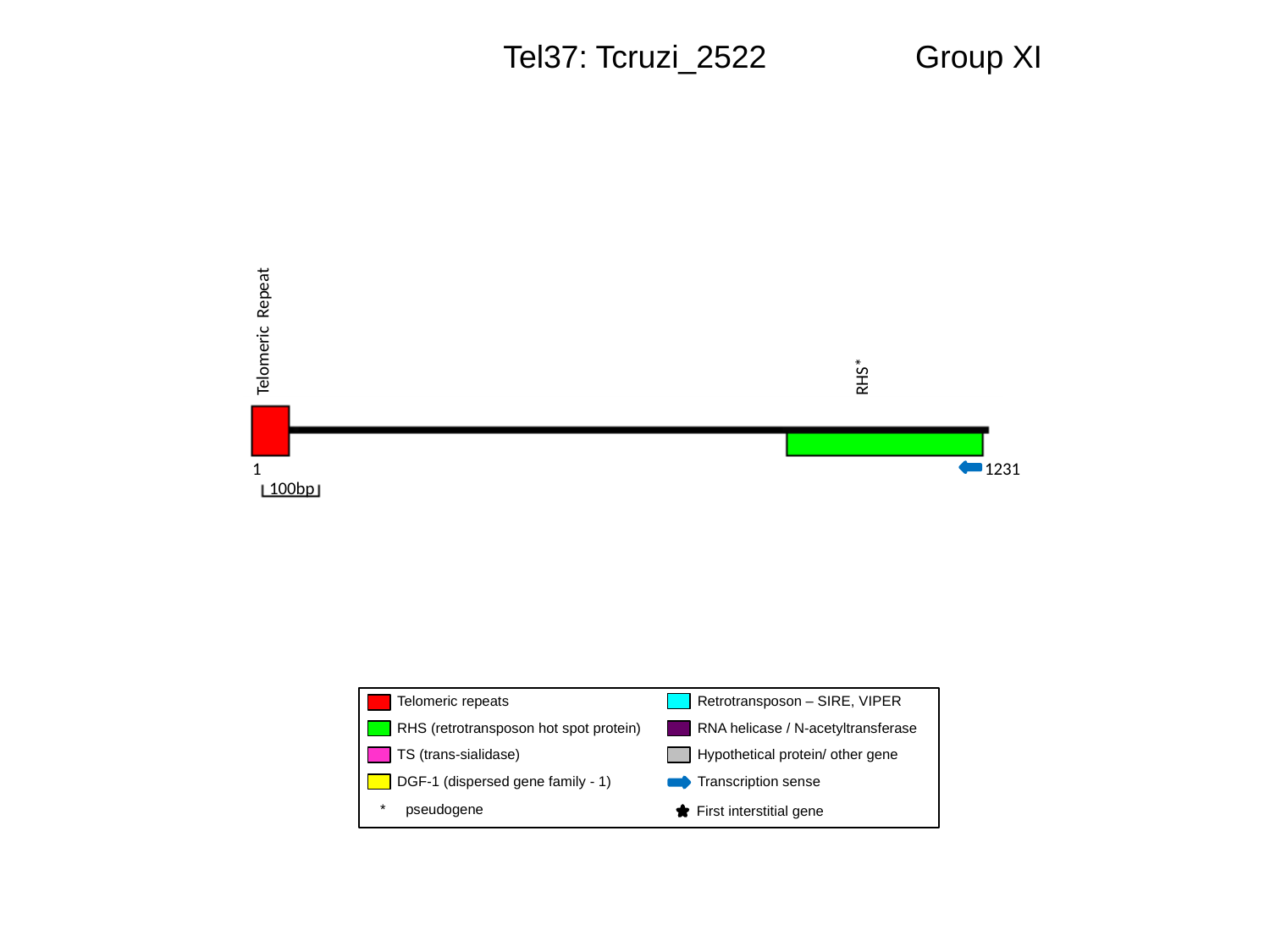

Tel37: Tcruzi_2522
Group XI
Telomeric Repeat
RHS*
1231
1
100bp
Telomeric repeats
Retrotransposon – SIRE, VIPER
RHS (retrotransposon hot spot protein)
RNA helicase / N-acetyltransferase
TS (trans-sialidase)
Hypothetical protein/ other gene
DGF-1 (dispersed gene family - 1)
Transcription sense
* pseudogene
First interstitial gene

## Slide 38
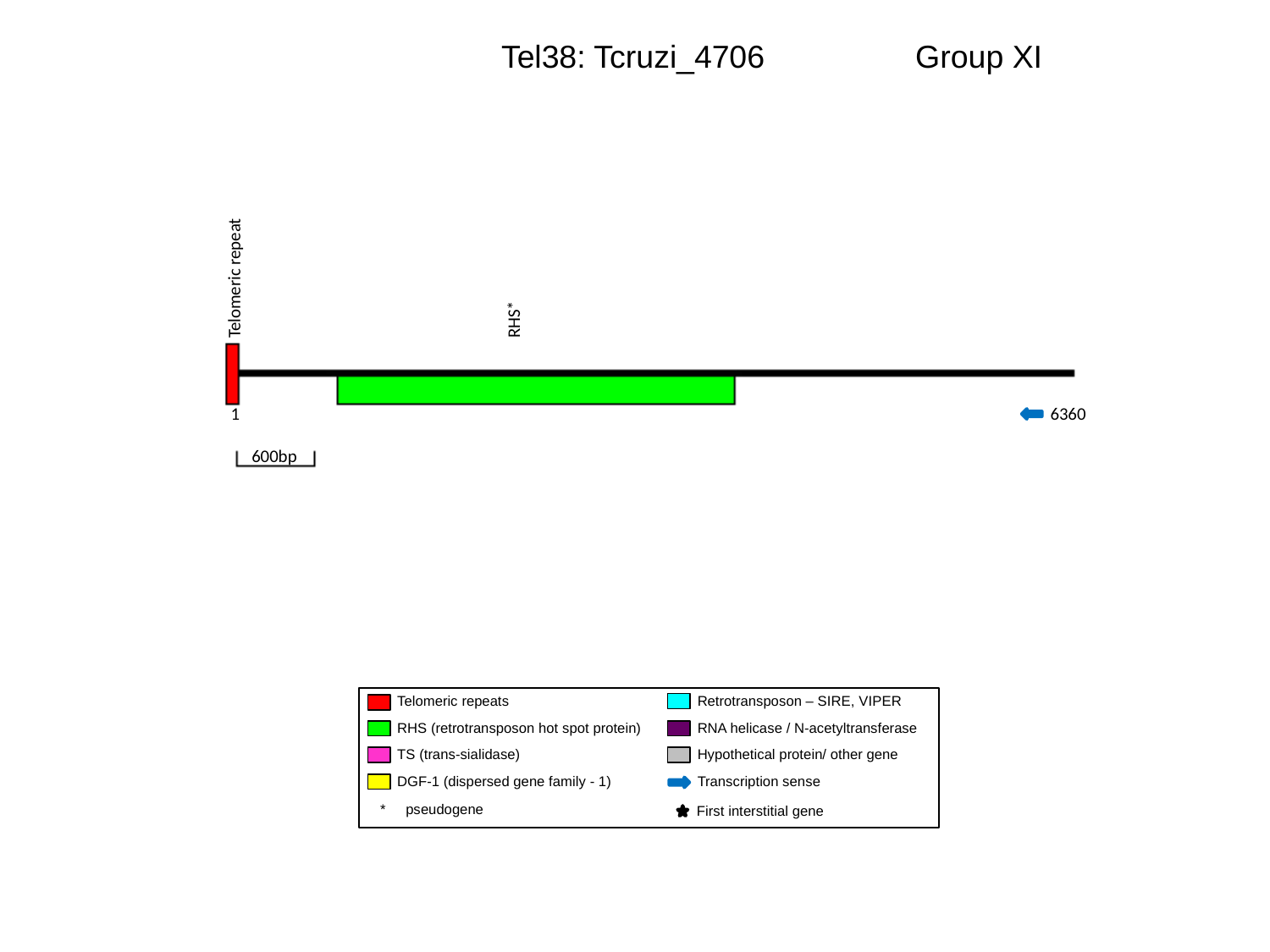

Group XI
Tel38: Tcruzi_4706
Telomeric repeat
RHS*
6360
1
600bp
Telomeric repeats
Retrotransposon – SIRE, VIPER
RHS (retrotransposon hot spot protein)
RNA helicase / N-acetyltransferase
TS (trans-sialidase)
Hypothetical protein/ other gene
DGF-1 (dispersed gene family - 1)
Transcription sense
* pseudogene
First interstitial gene

## Slide 39
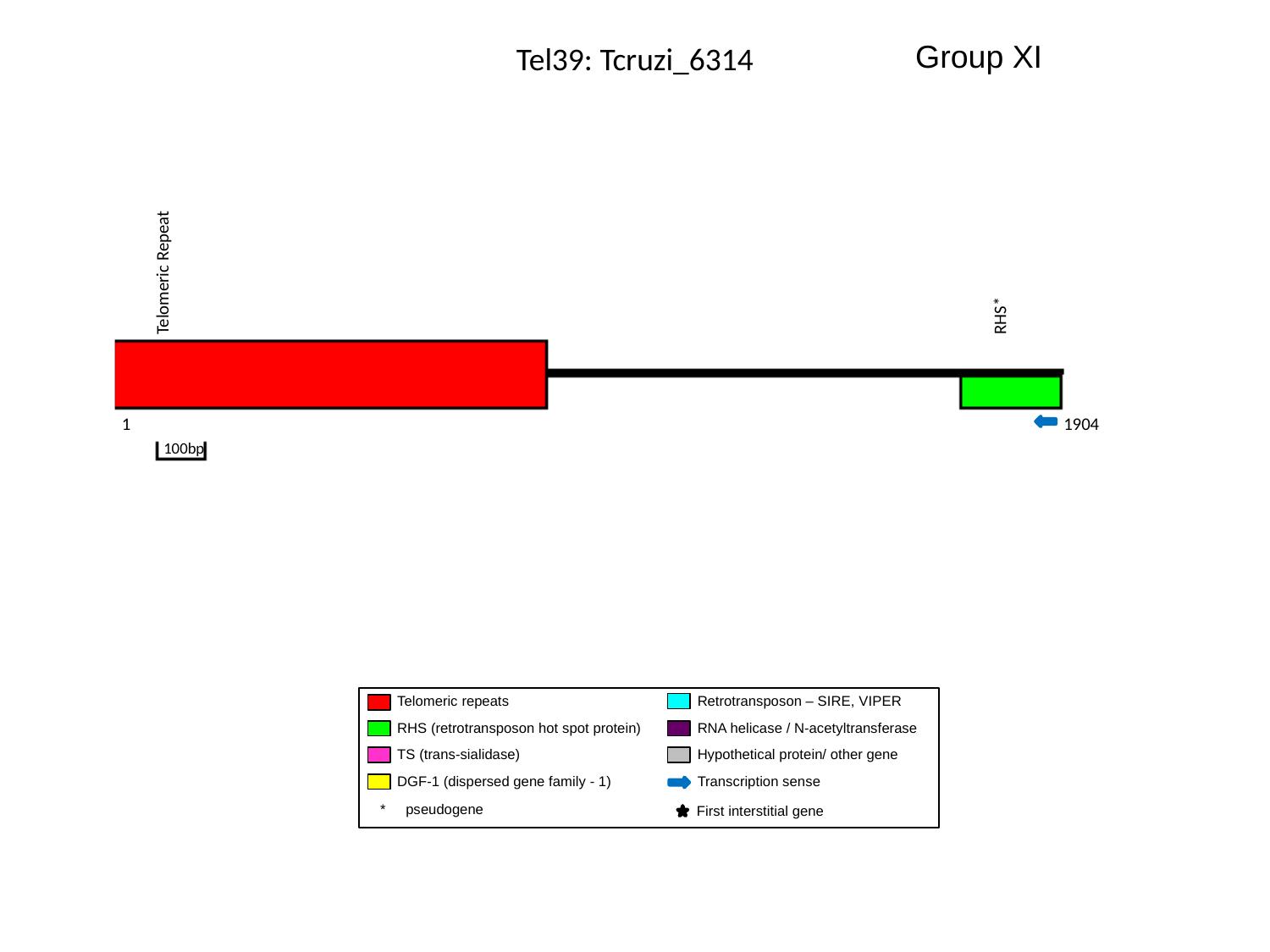

Group XI
Tel39: Tcruzi_6314
Telomeric Repeat
RHS*
1
1904
100bp
Telomeric repeats
Retrotransposon – SIRE, VIPER
RHS (retrotransposon hot spot protein)
RNA helicase / N-acetyltransferase
TS (trans-sialidase)
Hypothetical protein/ other gene
DGF-1 (dispersed gene family - 1)
Transcription sense
* pseudogene
First interstitial gene

## Slide 40
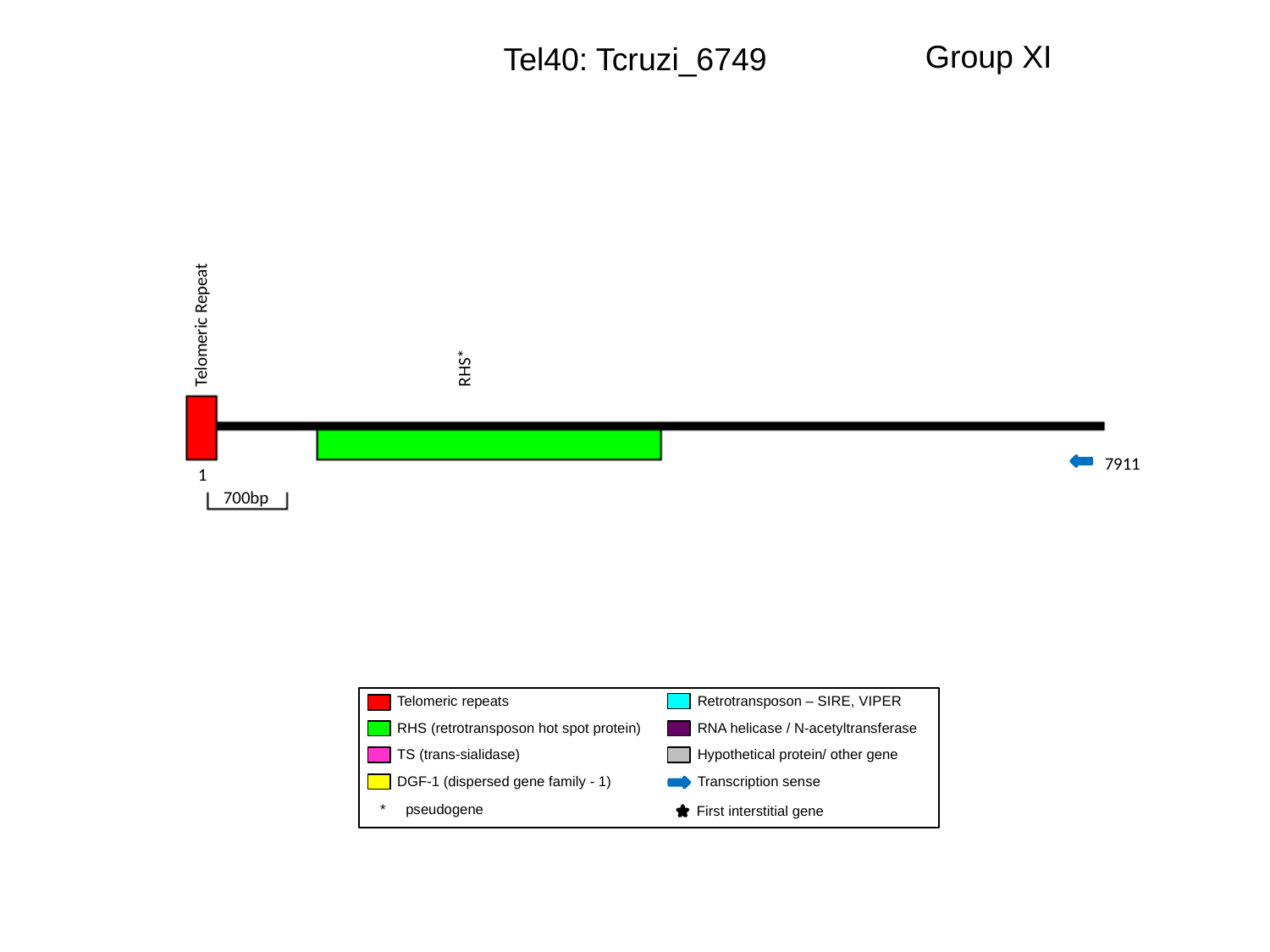

Group XI
Tel40: Tcruzi_6749
Telomeric Repeat
RHS*
7911
1
700bp
Telomeric repeats
Retrotransposon – SIRE, VIPER
RHS (retrotransposon hot spot protein)
RNA helicase / N-acetyltransferase
TS (trans-sialidase)
Hypothetical protein/ other gene
DGF-1 (dispersed gene family - 1)
Transcription sense
* pseudogene
First interstitial gene

## Slide 41
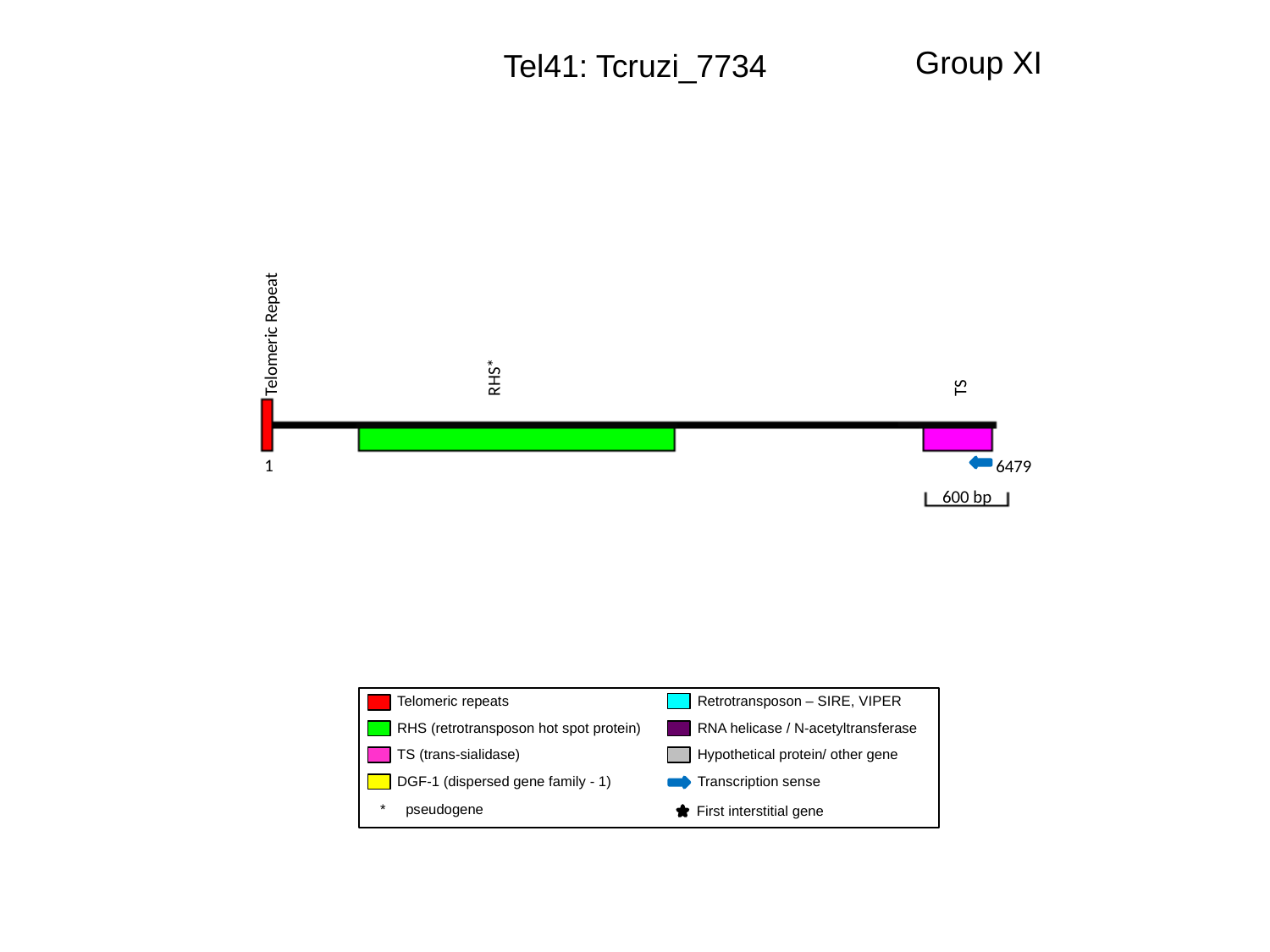

Group XI
Tel41: Tcruzi_7734
Telomeric Repeat
RHS*
TS
1
6479
600 bp
Telomeric repeats
Retrotransposon – SIRE, VIPER
RHS (retrotransposon hot spot protein)
RNA helicase / N-acetyltransferase
TS (trans-sialidase)
Hypothetical protein/ other gene
DGF-1 (dispersed gene family - 1)
Transcription sense
* pseudogene
First interstitial gene

## Slide 42
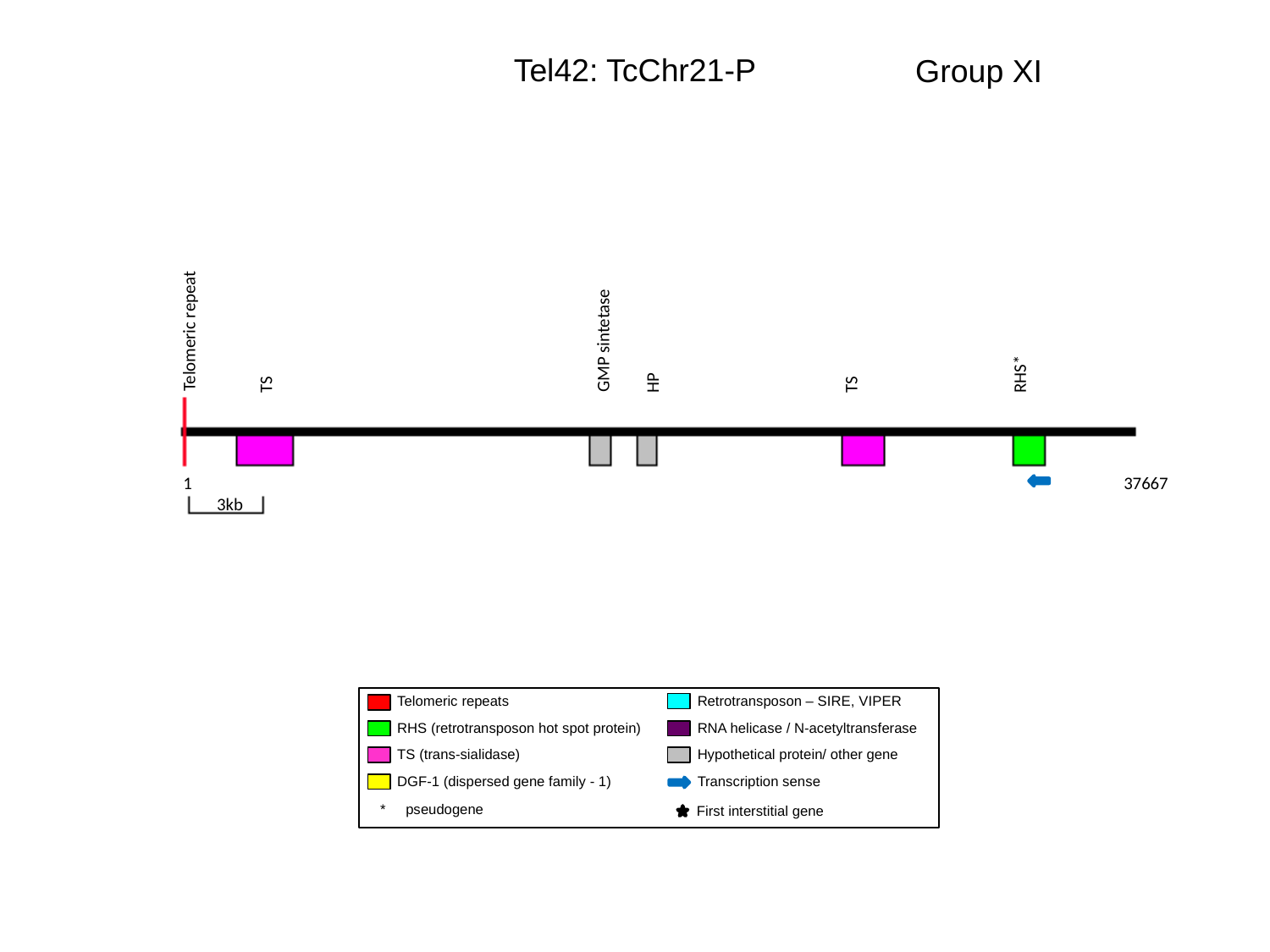

Tel42: TcChr21-P
Group XI
Telomeric repeat
GMP sintetase
RHS*
TS
TS
HP
1
37667
3kb
Telomeric repeats
Retrotransposon – SIRE, VIPER
RHS (retrotransposon hot spot protein)
RNA helicase / N-acetyltransferase
TS (trans-sialidase)
Hypothetical protein/ other gene
DGF-1 (dispersed gene family - 1)
Transcription sense
* pseudogene
First interstitial gene

## Slide 43
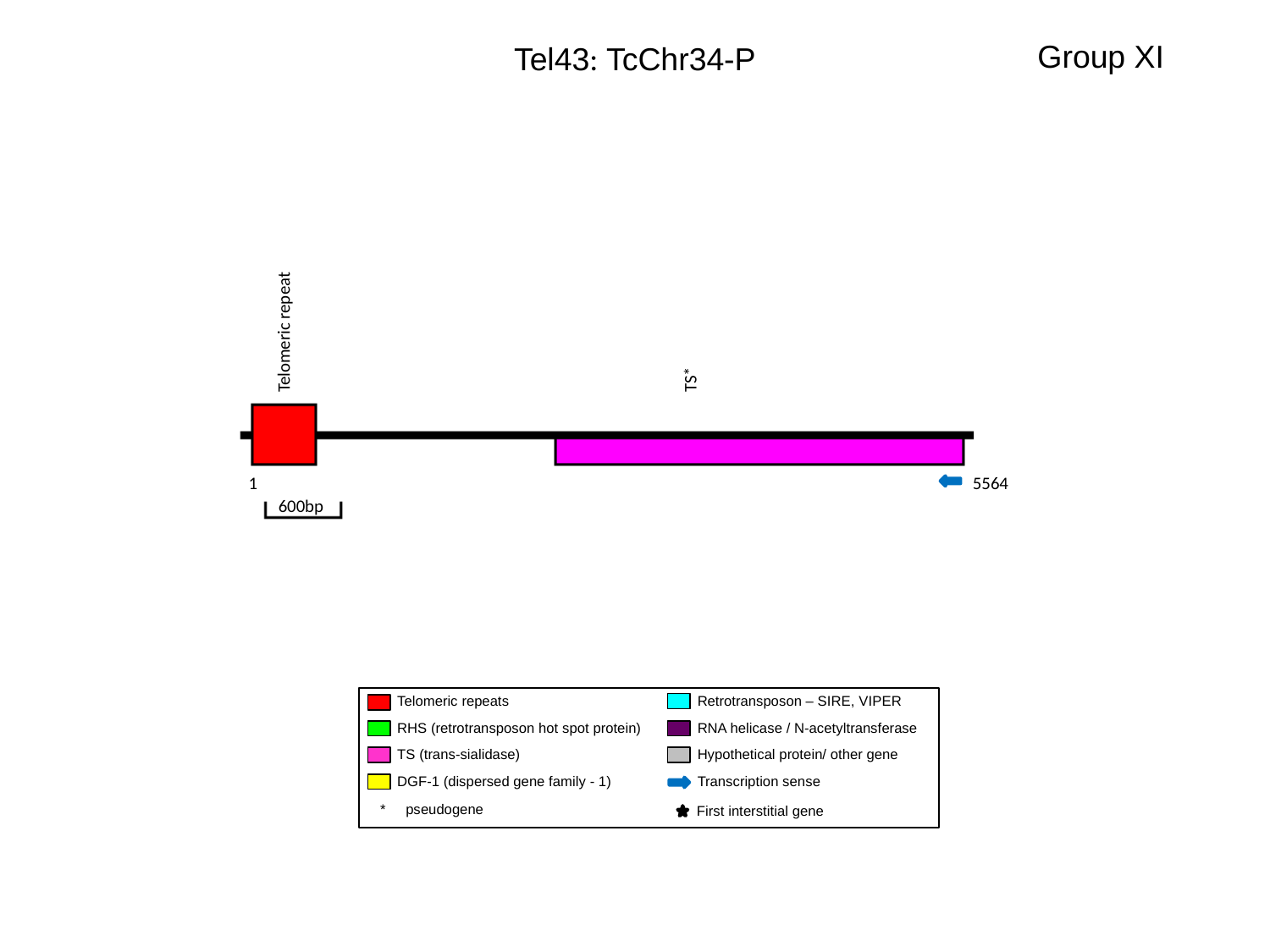

Group XI
Tel43: TcChr34-P
Telomeric repeat
TS*
1
5564
600bp
Telomeric repeats
Retrotransposon – SIRE, VIPER
RHS (retrotransposon hot spot protein)
RNA helicase / N-acetyltransferase
TS (trans-sialidase)
Hypothetical protein/ other gene
DGF-1 (dispersed gene family - 1)
Transcription sense
* pseudogene
First interstitial gene

## Slide 44
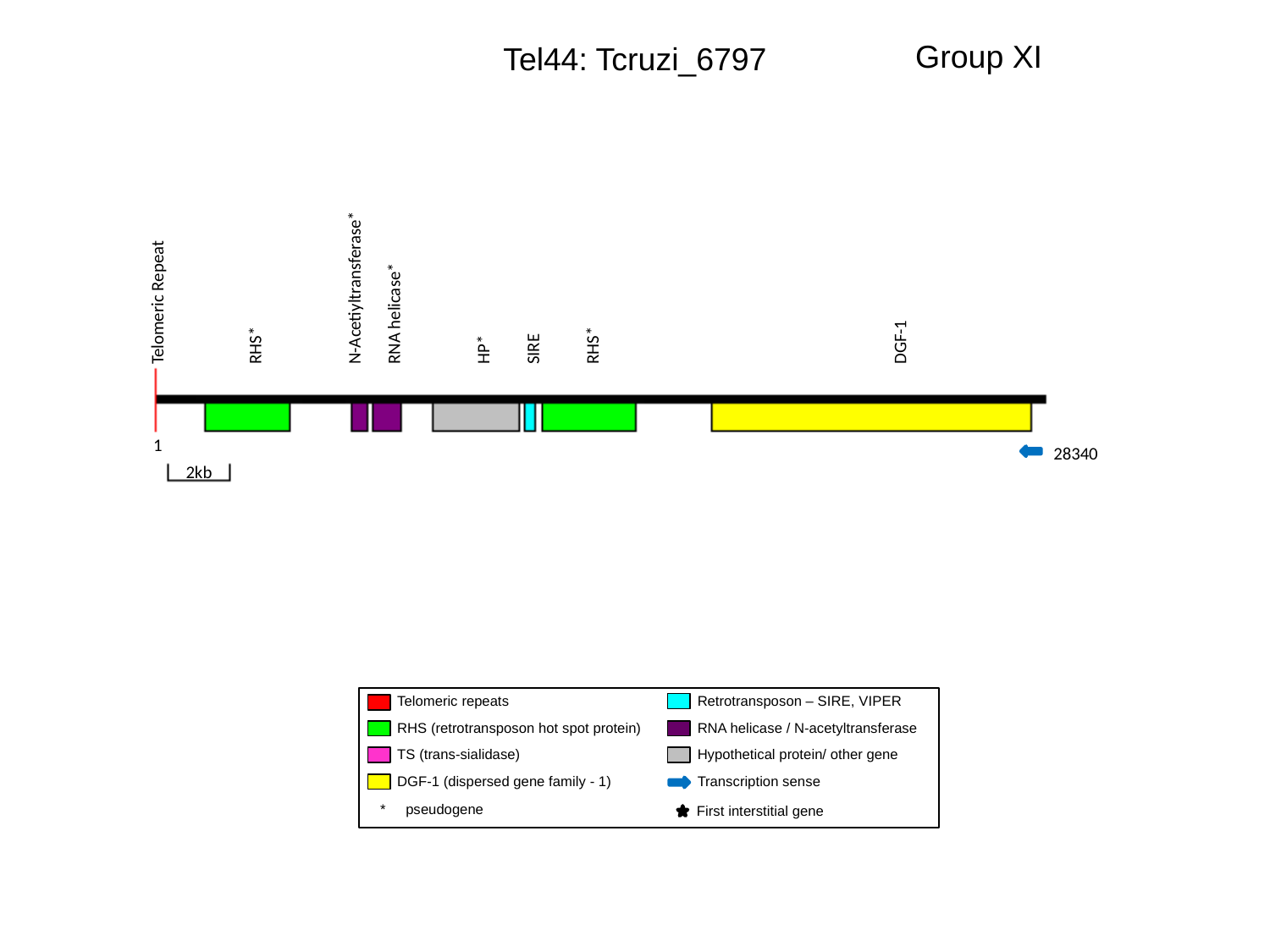

Group XI
Tel44: Tcruzi_6797
Telomeric Repeat
RHS*
N-Acetiyltransferase*
RNA helicase*
HP*
SIRE
RHS*
DGF-1
1
28340
2kb
Telomeric repeats
Retrotransposon – SIRE, VIPER
RHS (retrotransposon hot spot protein)
RNA helicase / N-acetyltransferase
TS (trans-sialidase)
Hypothetical protein/ other gene
DGF-1 (dispersed gene family - 1)
Transcription sense
* pseudogene
First interstitial gene

## Slide 45
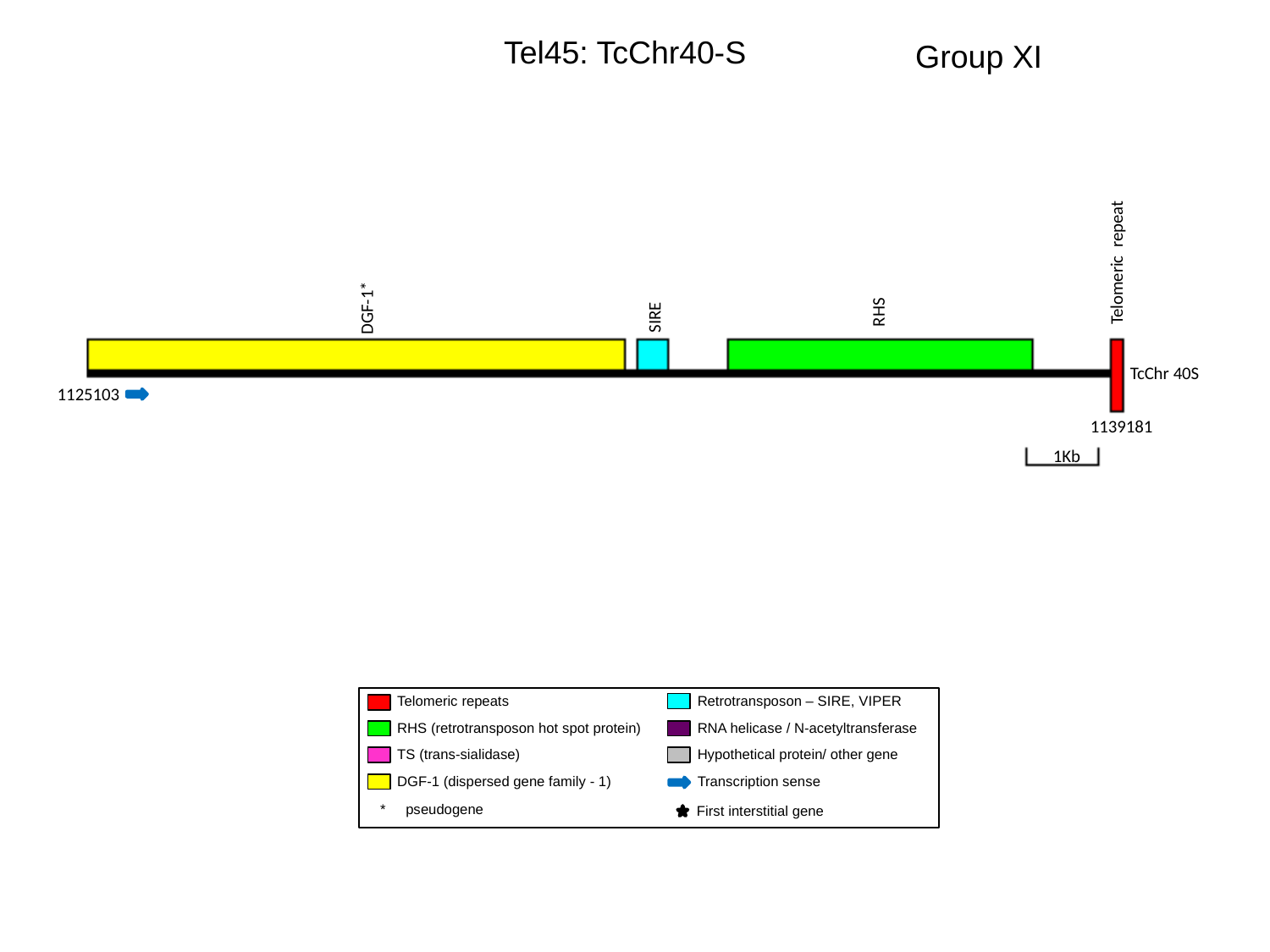

Tel45: TcChr40-S
Group XI
Telomeric repeat
DGF-1*
RHS
SIRE
TcChr 40S
1125103
1139181
1Kb
Telomeric repeats
Retrotransposon – SIRE, VIPER
RHS (retrotransposon hot spot protein)
RNA helicase / N-acetyltransferase
TS (trans-sialidase)
Hypothetical protein/ other gene
DGF-1 (dispersed gene family - 1)
Transcription sense
* pseudogene
First interstitial gene

## Slide 46
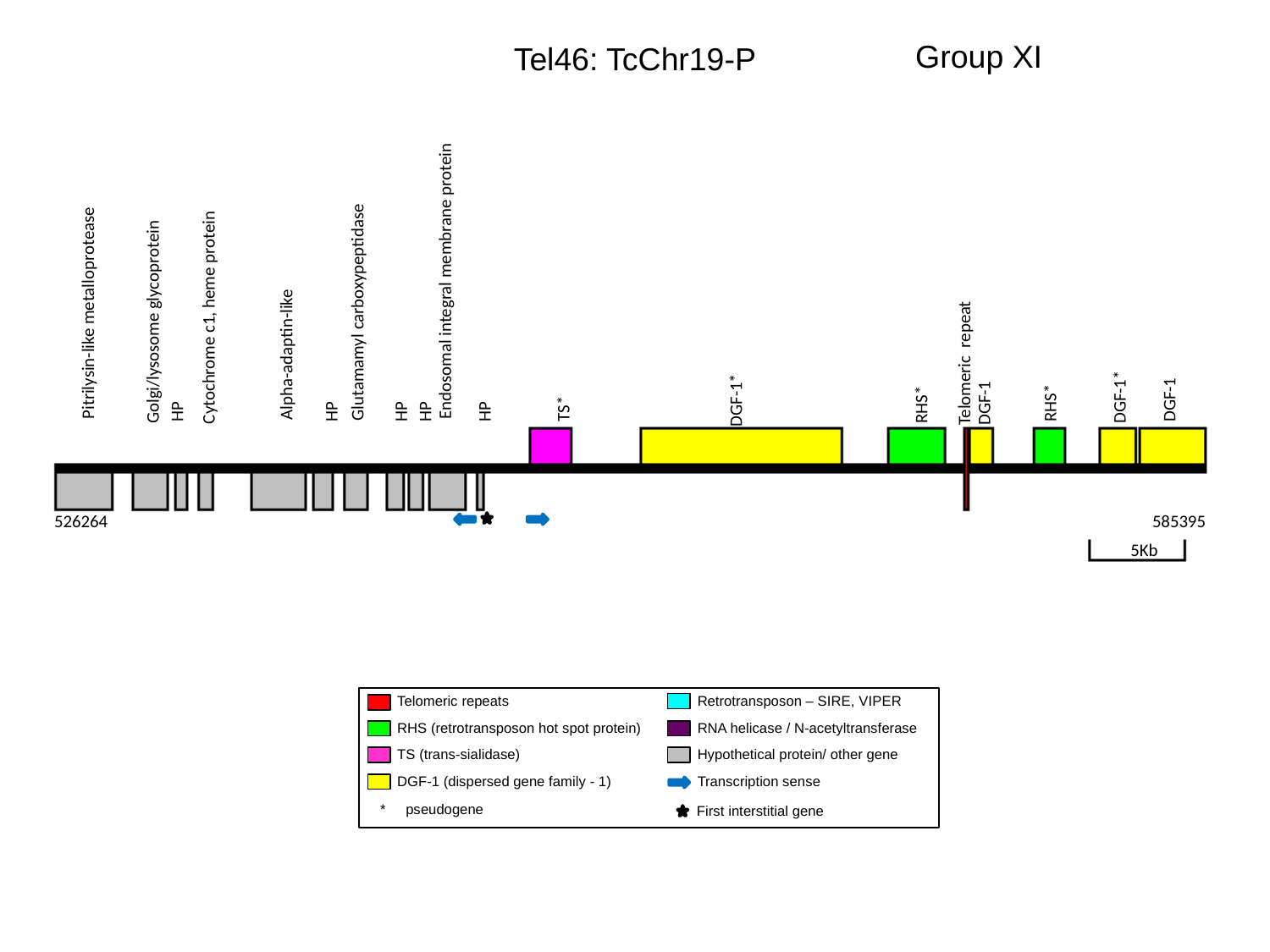

Group XI
Tel46: TcChr19-P
Endosomal integral membrane protein
Glutamamyl carboxypeptidase
Pitrilysin-like metalloprotease
Cytochrome c1, heme protein
Golgi/lysosome glycoprotein
DGF-1
HP
HP
HP
HP
TS*
RHS*
HP
RHS*
DGF-1*
Telomeric repeat
DGF-1
DGF-1*
Alpha-adaptin-like
526264
585395
5Kb
Telomeric repeats
Retrotransposon – SIRE, VIPER
RHS (retrotransposon hot spot protein)
RNA helicase / N-acetyltransferase
TS (trans-sialidase)
Hypothetical protein/ other gene
DGF-1 (dispersed gene family - 1)
Transcription sense
* pseudogene
First interstitial gene

## Slide 47
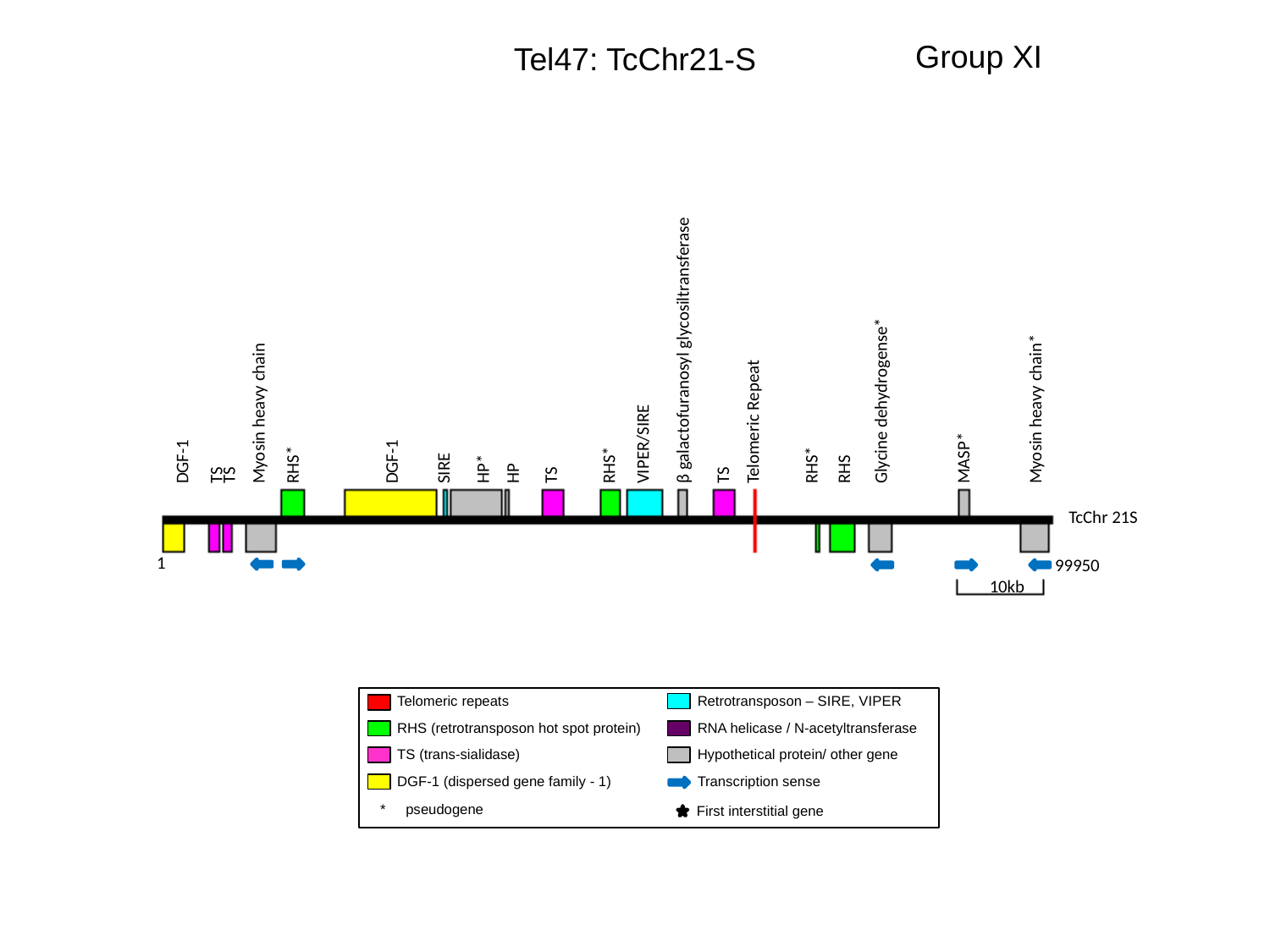

Group XI
Tel47: TcChr21-S
β galactofuranosyl glycosiltransferase
RHS*
SIRE
HP*
HP
VIPER/SIRE
TS
Telomeric Repeat
Myosin heavy chain
Glycine dehydrogense*
MASP*
Myosin heavy chain*
RHS*
RHS*
RHS
DGF-1
DGF-1
TS
TS
TS
TcChr 21S
1
99950
10kb
Telomeric repeats
Retrotransposon – SIRE, VIPER
RHS (retrotransposon hot spot protein)
RNA helicase / N-acetyltransferase
TS (trans-sialidase)
Hypothetical protein/ other gene
DGF-1 (dispersed gene family - 1)
Transcription sense
* pseudogene
First interstitial gene

## Slide 48
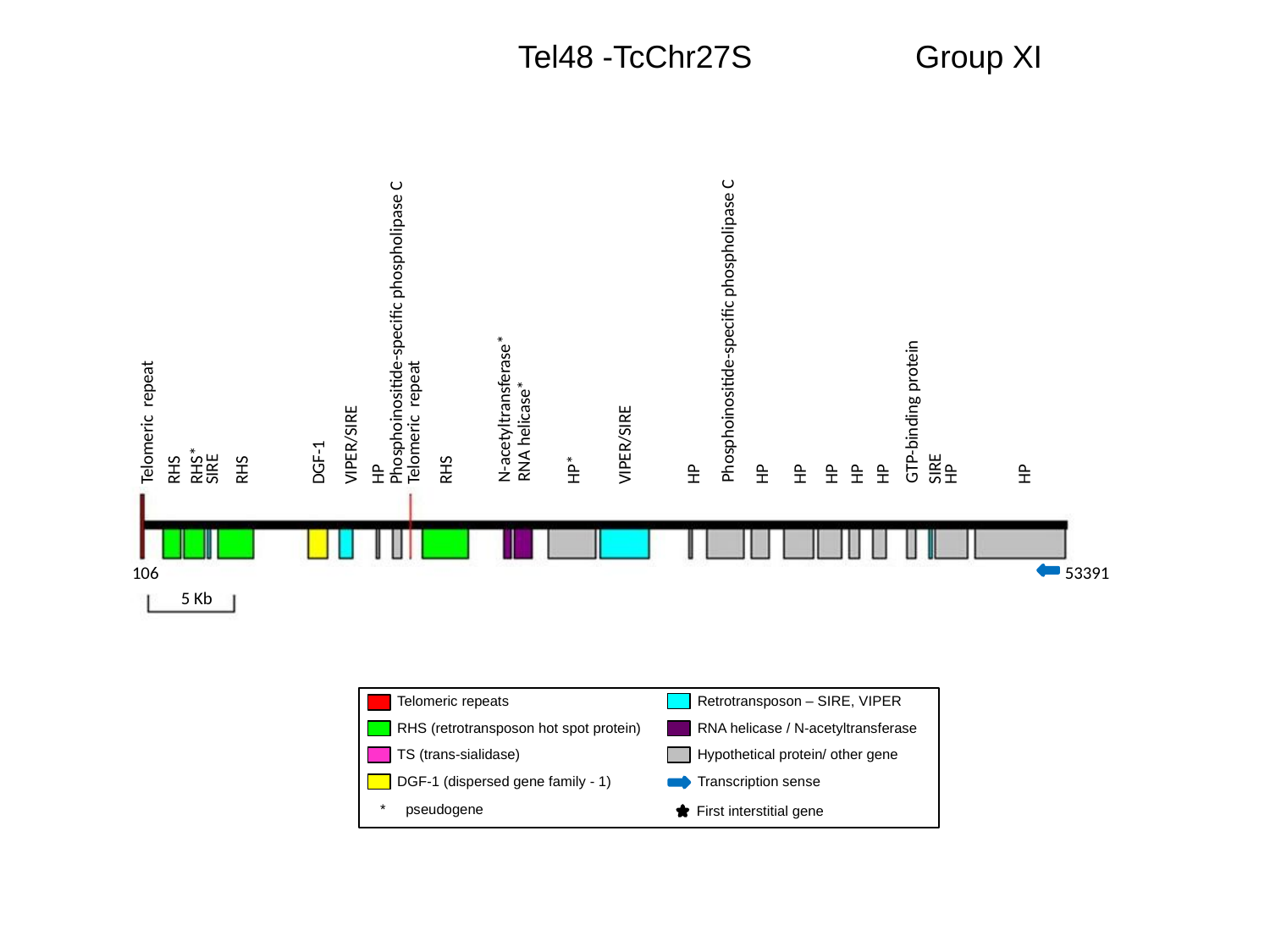

Tel48 -TcChr27S
Group XI
Phosphoinositide-specific phospholipase C
Phosphoinositide-specific phospholipase C
Telomeric repeat
Telomeric repeat
N-acetyltransferase*
GTP-binding protein
RNA helicase*
VIPER/SIRE
VIPER/SIRE
RHS
SIRE
HP
RHS
HP
HP
HP
HP
HP
RHS*
RHS
DGF-1
HP*
HP
HP
HP
SIRE
106
53391
5 Kb
Telomeric repeats
Retrotransposon – SIRE, VIPER
RHS (retrotransposon hot spot protein)
RNA helicase / N-acetyltransferase
TS (trans-sialidase)
Hypothetical protein/ other gene
DGF-1 (dispersed gene family - 1)
Transcription sense
* pseudogene
First interstitial gene

## Slide 49
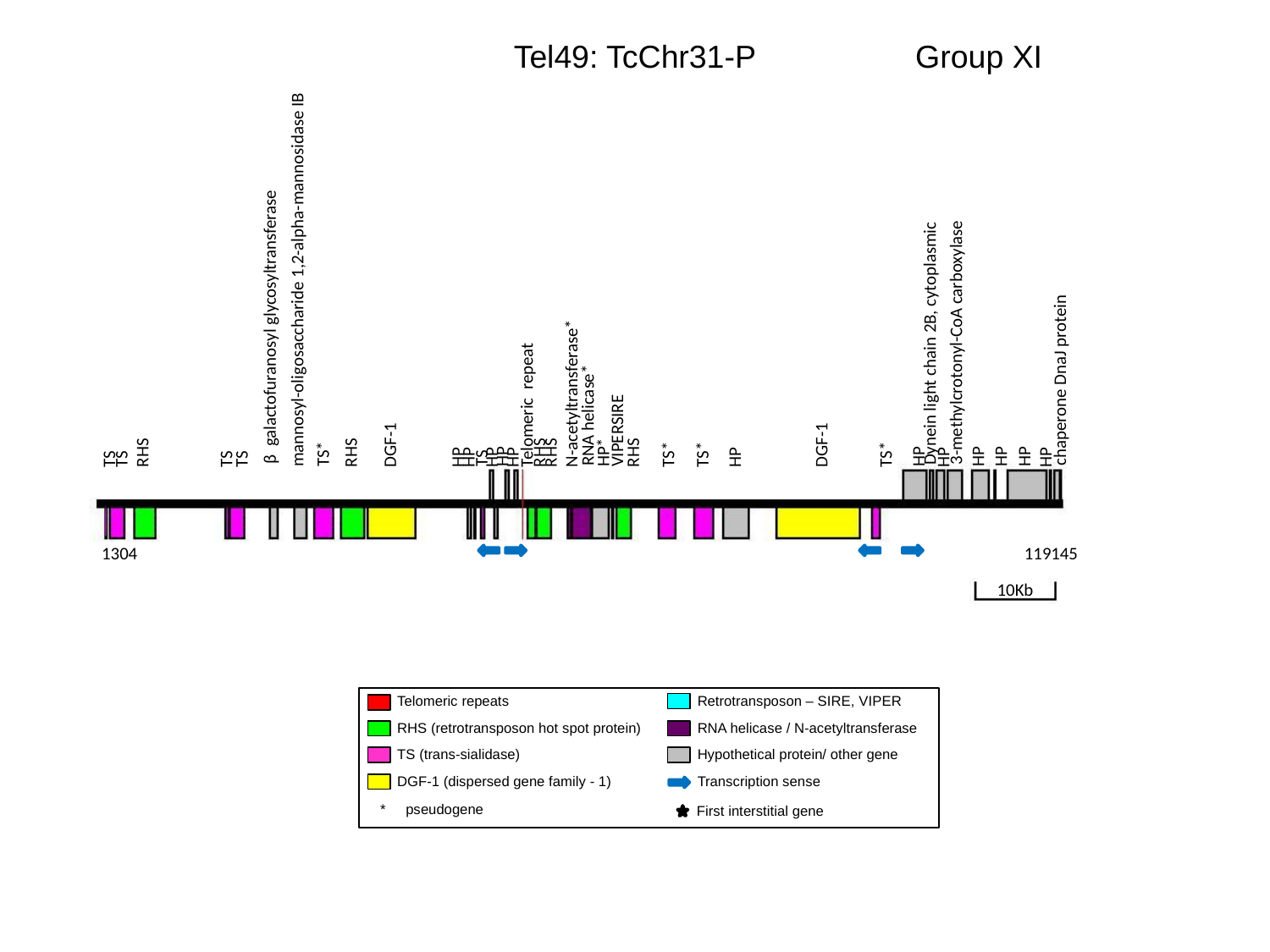

Tel49: TcChr31-P
Group XI
mannosyl-oligosaccharide 1,2-alpha-mannosidase IB
HP
β galactofuranosyl glycosyltransferase
HP
3-methylcrotonyl-CoA carboxylase
Dynein light chain 2B, cytoplasmic
Telomeric repeat
VIPERSIRE
chaperone DnaJ protein
N-acetyltransferase*
HP
HP
RNA helicase*
	DGF-1
HP
HP
TS*
HP
HP
TS
RHS
TS*
TS
RHS
TS
TS
RHS
RHS
RHS
TS*
DGF-1
HP
TS*
HP
TS
HP
HP*
HP
1304
119145
10Kb
Telomeric repeats
Retrotransposon – SIRE, VIPER
RHS (retrotransposon hot spot protein)
RNA helicase / N-acetyltransferase
TS (trans-sialidase)
Hypothetical protein/ other gene
DGF-1 (dispersed gene family - 1)
Transcription sense
* pseudogene
First interstitial gene
