# Supplementary material for: Anatomy and evolution of telomeric and subtelomeric regions in the human protozoan parasite Trypanosoma cruzi
Source: BMC Genomics. 2012 Jun 8;13:229. doi: 10.1186/1471-2164-13-229 (PMC3418195; doi:10.1186/1471-2164-13-229)
Supplement: Additional file 3 — Synteny analysis between homologous chromosome ends ofT. cruzi. Synteny analysis between the homologous chromosome ends listed in Table 3. The red lines represent regions of homology between the contigs. The annotated genes are indicated by colored boxes. [file 1471-2164-13-229-S3.pptx]

## Slide 1
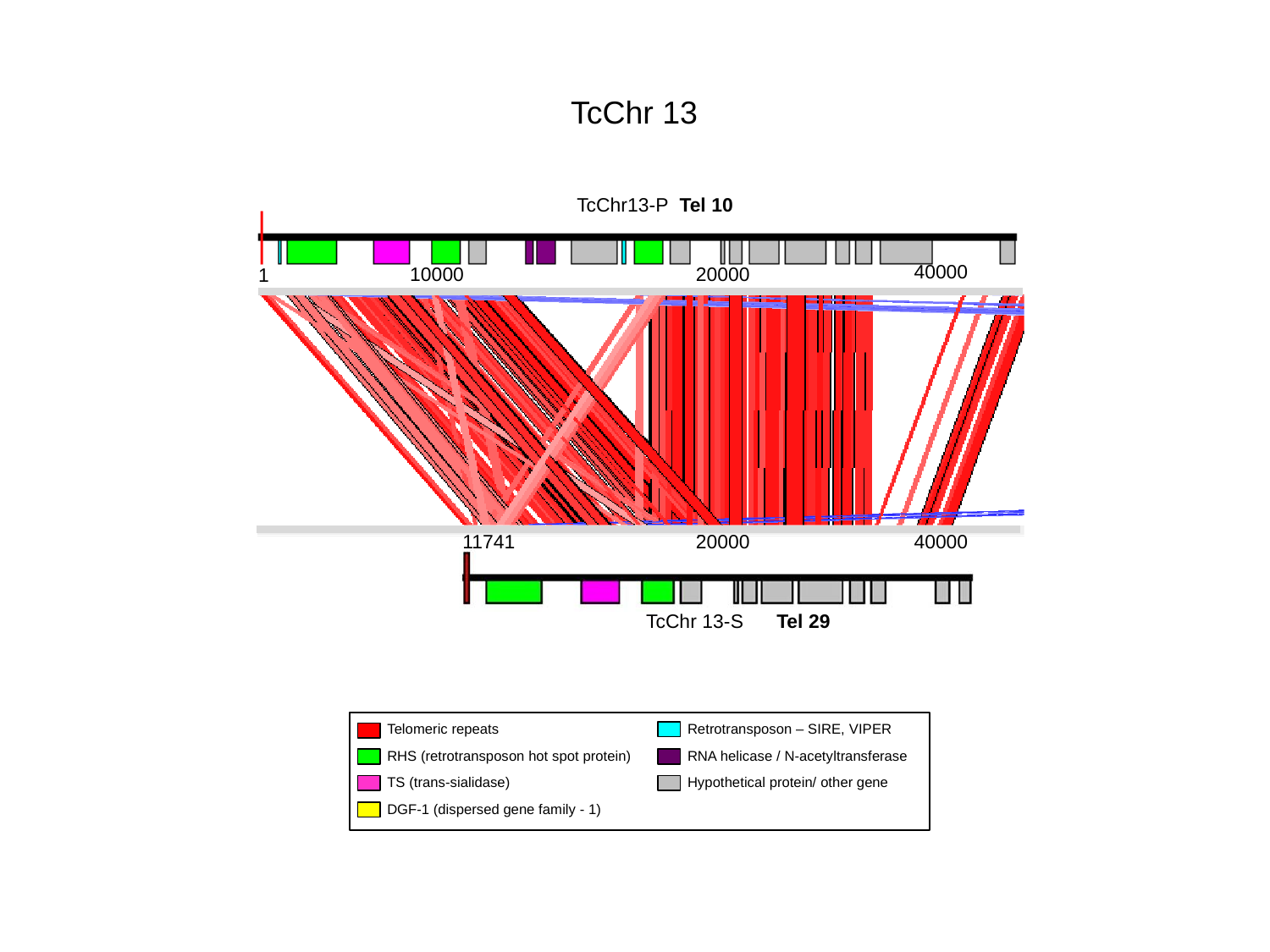

TcChr 13
TcChr13-P Tel 10
40000
10000
20000
1
11741
20000
40000
TcChr 13-S Tel 29
Telomeric repeats
Retrotransposon – SIRE, VIPER
RHS (retrotransposon hot spot protein)
RNA helicase / N-acetyltransferase
TS (trans-sialidase)
Hypothetical protein/ other gene
DGF-1 (dispersed gene family - 1)

## Slide 2
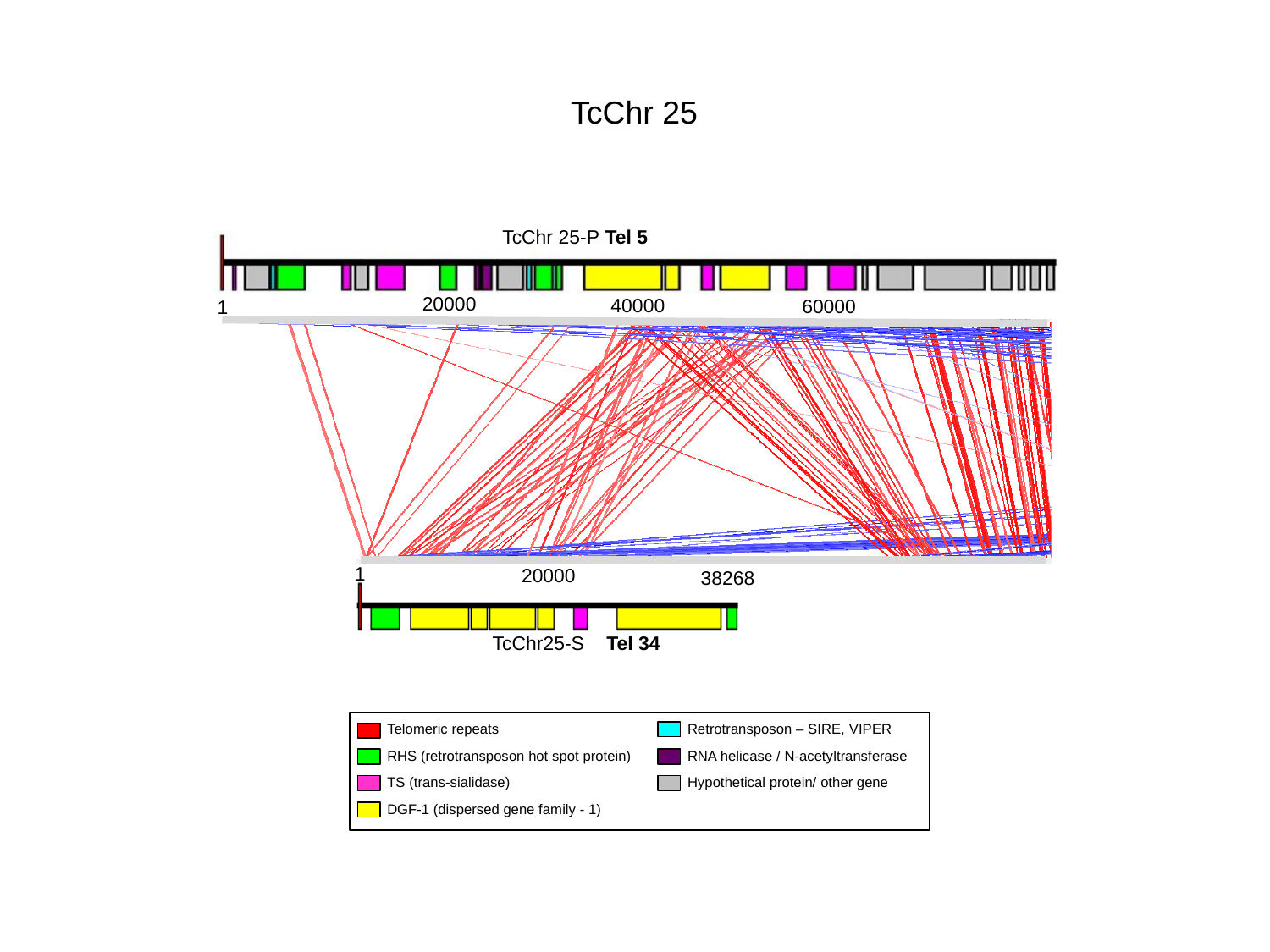

TcChr 25
TcChr 25-P Tel 5
20000
40000
60000
1
1
20000
38268
TcChr25-S Tel 34
Telomeric repeats
Retrotransposon – SIRE, VIPER
RHS (retrotransposon hot spot protein)
RNA helicase / N-acetyltransferase
TS (trans-sialidase)
Hypothetical protein/ other gene
DGF-1 (dispersed gene family - 1)

## Slide 3
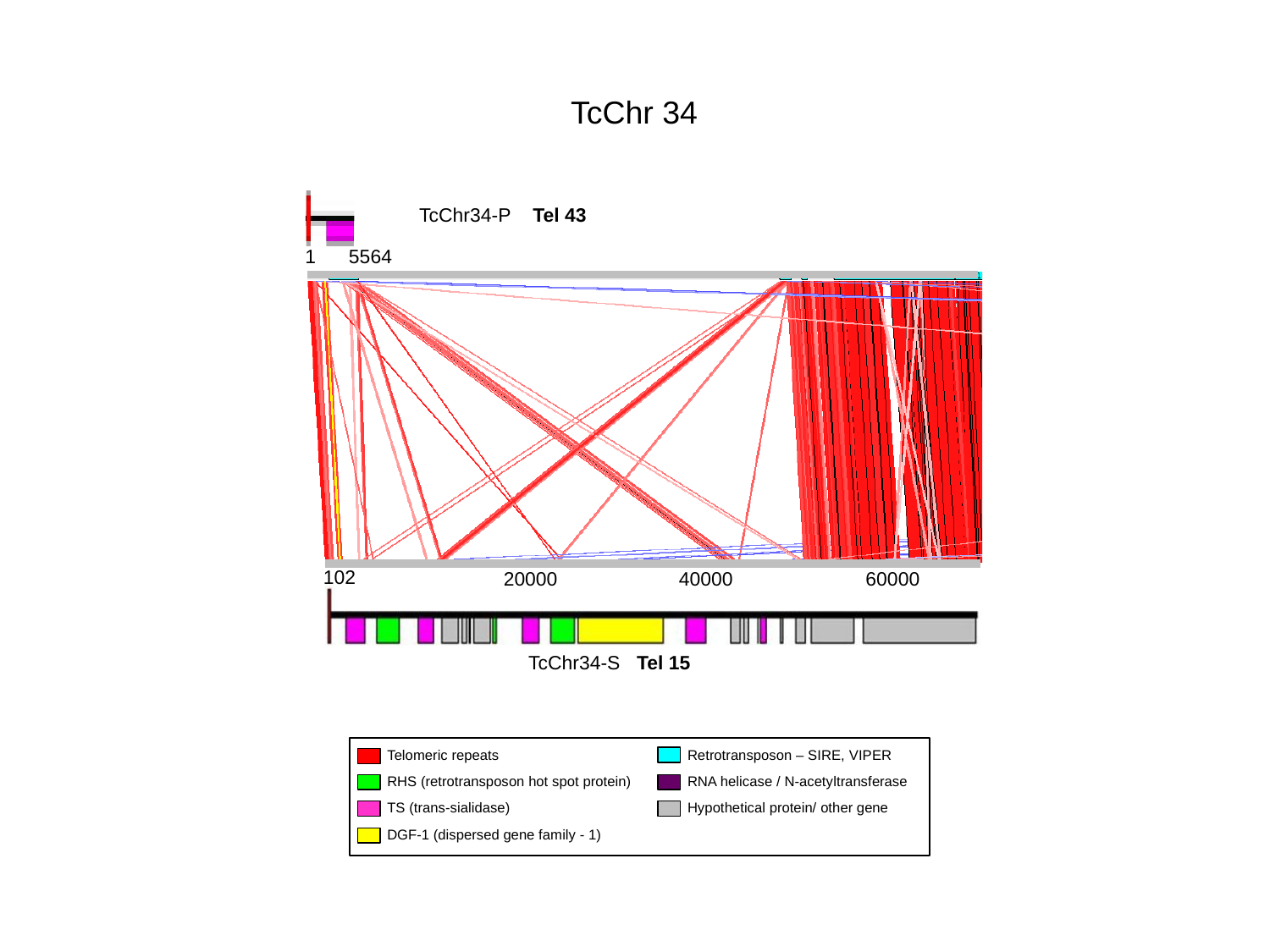

TcChr 34
TcChr34-P Tel 43
1 5564
102
60000
20000
40000
TcChr34-S Tel 15
Telomeric repeats
Retrotransposon – SIRE, VIPER
RHS (retrotransposon hot spot protein)
RNA helicase / N-acetyltransferase
TS (trans-sialidase)
Hypothetical protein/ other gene
DGF-1 (dispersed gene family - 1)

## Slide 4
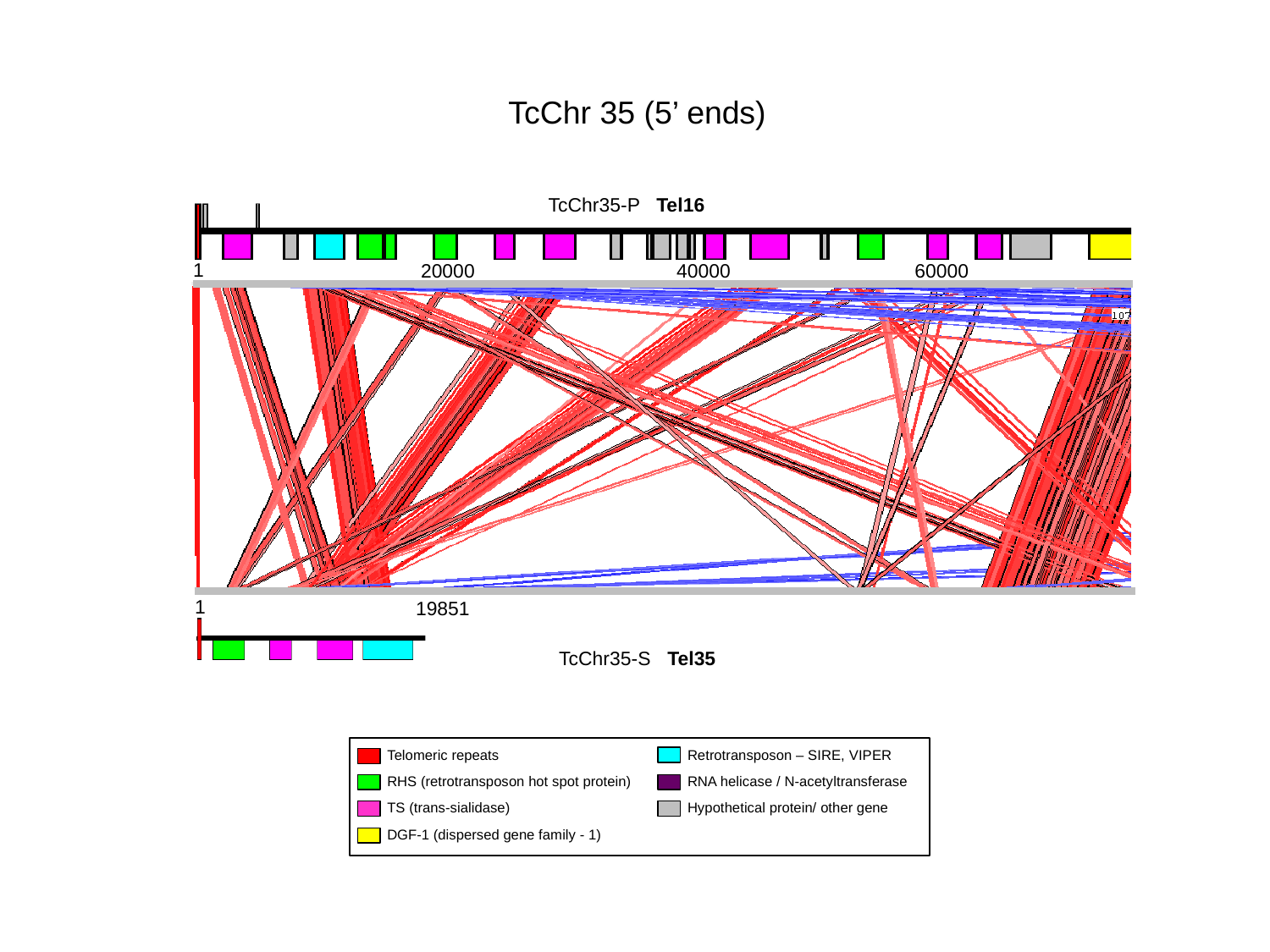

TcChr 35 (5’ ends)
TcChr35-P Tel16
1
20000
40000
60000
1
19851
TcChr35-S Tel35
Telomeric repeats
Retrotransposon – SIRE, VIPER
RHS (retrotransposon hot spot protein)
RNA helicase / N-acetyltransferase
TS (trans-sialidase)
Hypothetical protein/ other gene
DGF-1 (dispersed gene family - 1)

## Slide 5
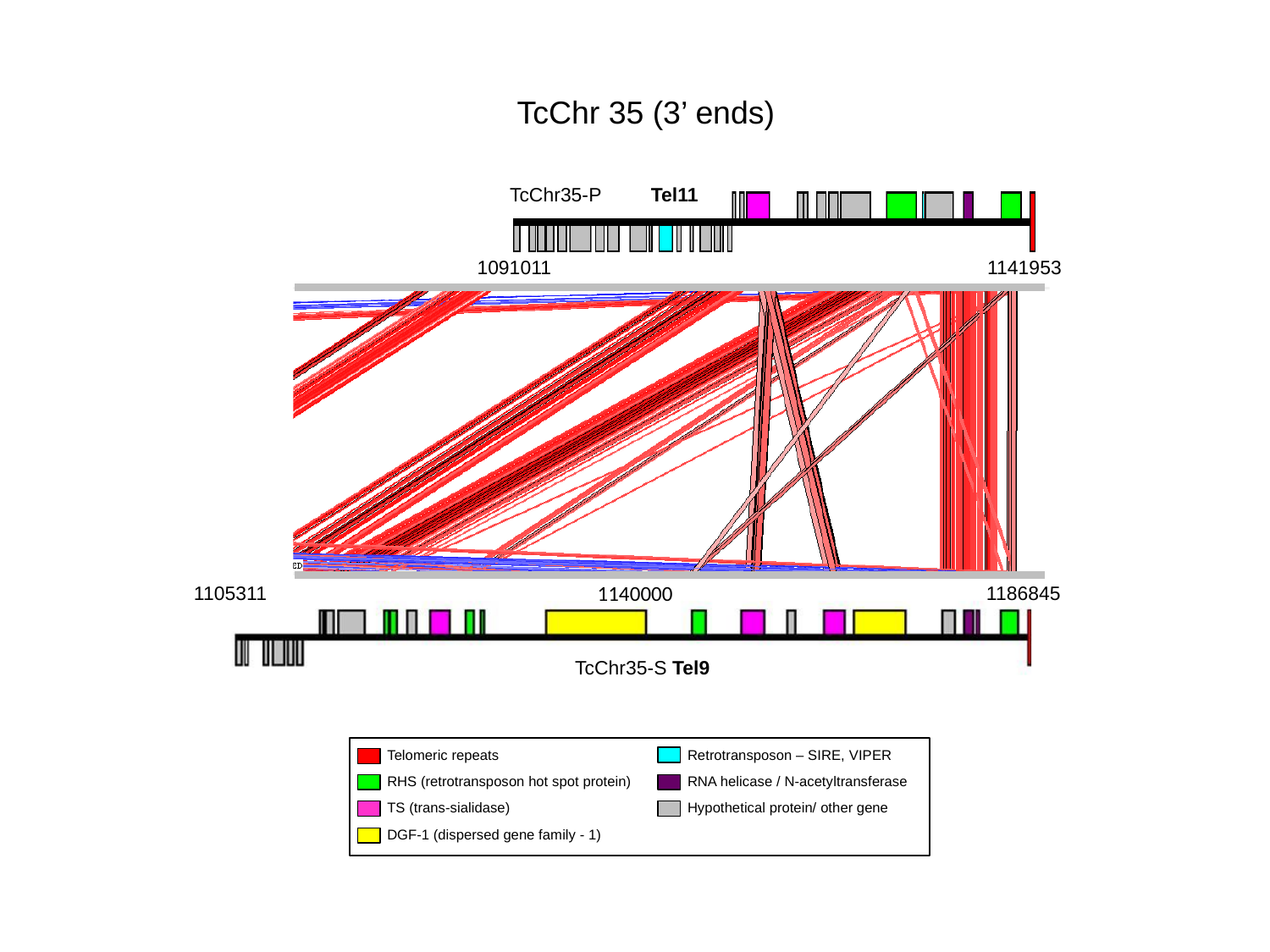

TcChr 35 (3’ ends)
TcChr35-P Tel11
1091011
1141953
1105311
1186845
TcChr35-S Tel9
1140000
Telomeric repeats
Retrotransposon – SIRE, VIPER
RHS (retrotransposon hot spot protein)
RNA helicase / N-acetyltransferase
TS (trans-sialidase)
Hypothetical protein/ other gene
DGF-1 (dispersed gene family - 1)

## Slide 6
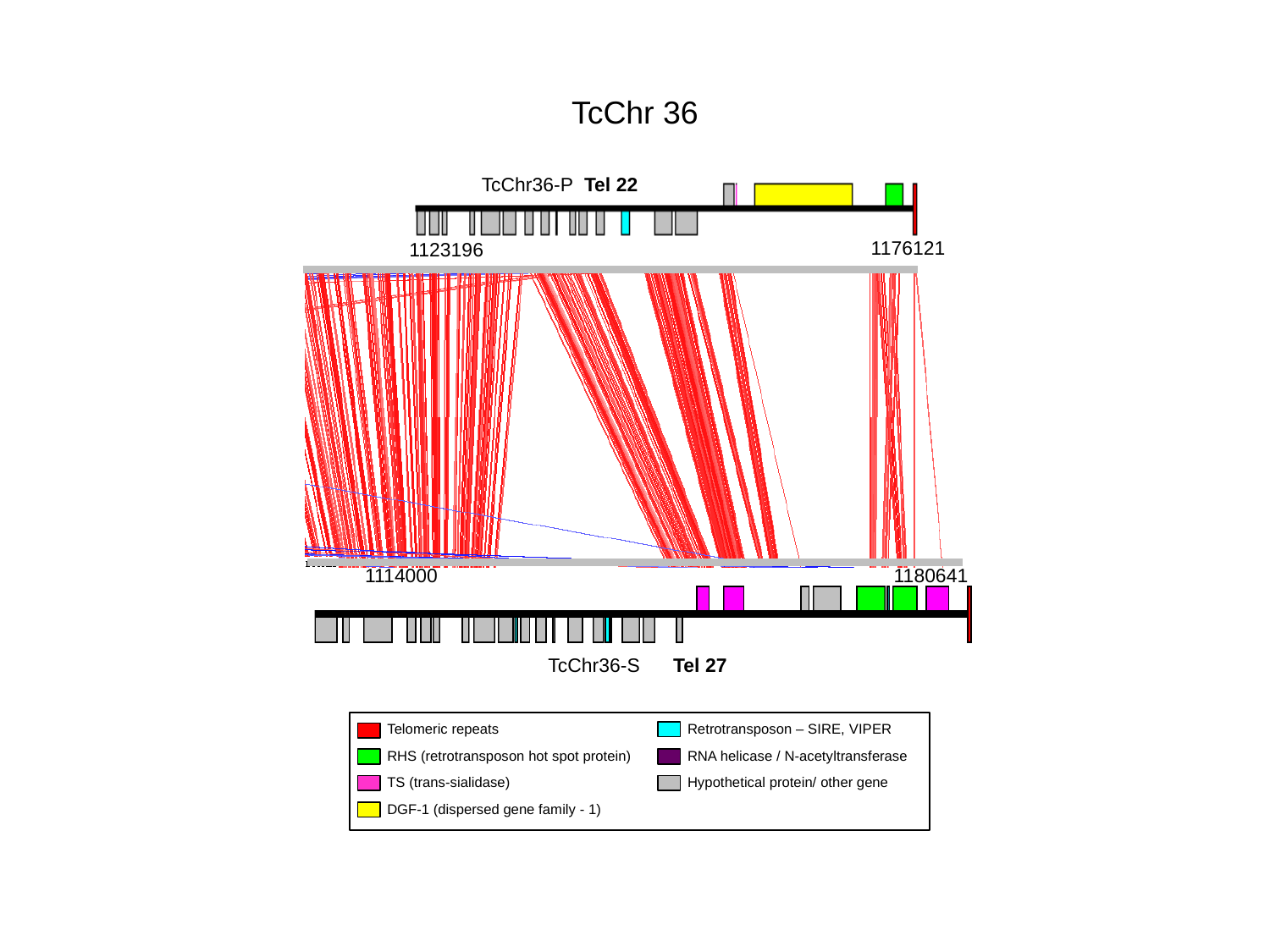

TcChr 36
TcChr36-P Tel 22
1176121
1123196
1114000
1180641
TcChr36-S Tel 27
Telomeric repeats
Retrotransposon – SIRE, VIPER
RHS (retrotransposon hot spot protein)
RNA helicase / N-acetyltransferase
TS (trans-sialidase)
Hypothetical protein/ other gene
DGF-1 (dispersed gene family - 1)

## Slide 7
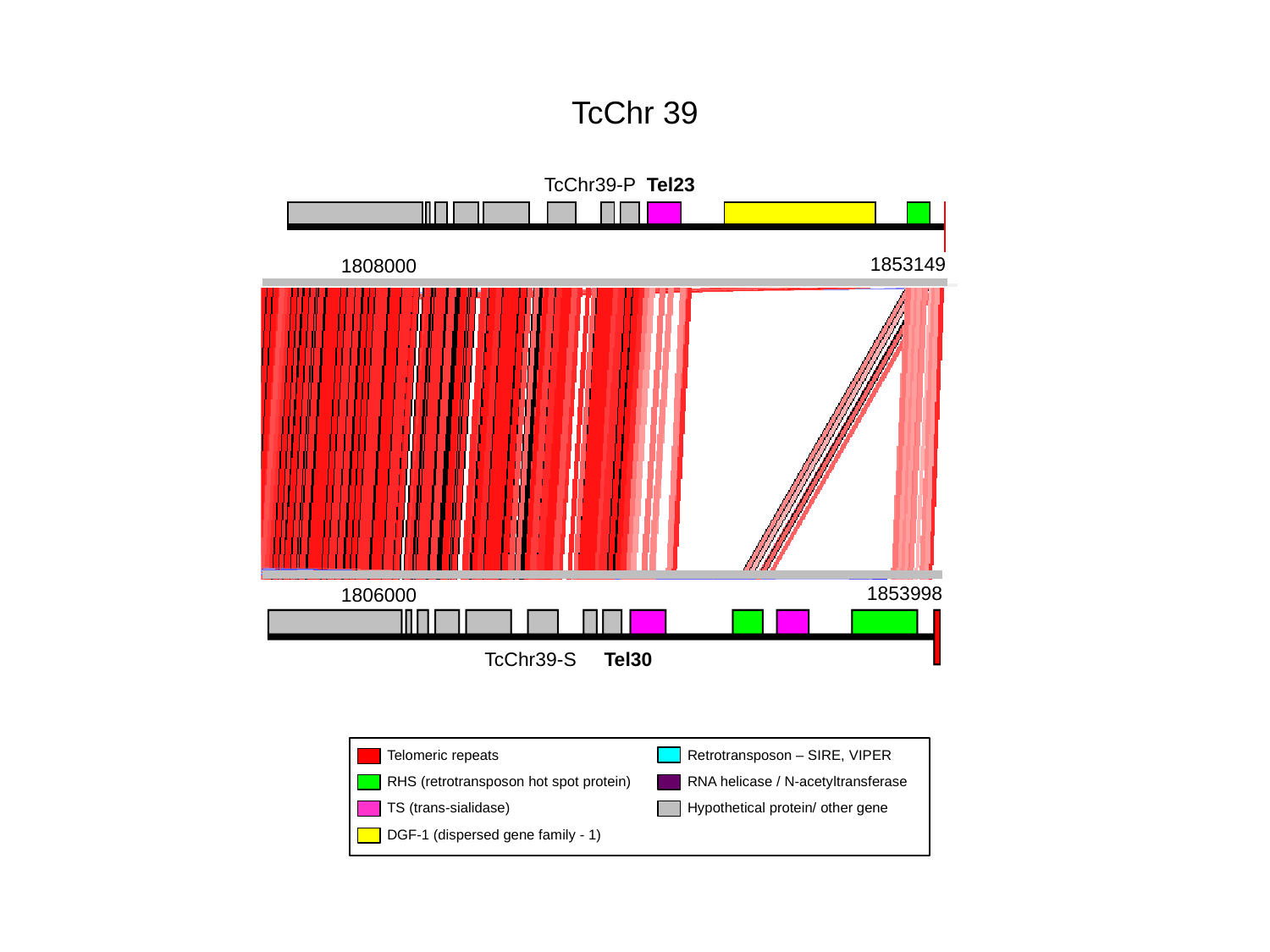

TcChr 39
TcChr39-P Tel23
1853149
1808000
1853998
1806000
TcChr39-S Tel30
Telomeric repeats
Retrotransposon – SIRE, VIPER
RHS (retrotransposon hot spot protein)
RNA helicase / N-acetyltransferase
TS (trans-sialidase)
Hypothetical protein/ other gene
DGF-1 (dispersed gene family - 1)
